# Treatment with *Bifidobacteria* can suppress Aβ accumulation and neuroinflammation in APP/PS1 mice

**DOI:** 10.7717/peerj.10262

**Published:** 2020-10-28

**Authors:** Qiong Wu, Qifa Li, Xuan Zhang, Michael Ntim, Xuefei Wu, Ming Li, Li Wang, Jie Zhao, Shao Li

**Affiliations:** 1 Liaoning Provincial Key Laboratory of Cerebral Diseases in Department of Physiology, Dalian Medical University, Dalian, China; 2 Functional Laboratory, Dalian Medical University, Dalian, China; 3 National-Local Joint Engineering Research Center for Drug-Research and Development (R & D) of Neurodegenerative Diseases, Dalian Medical University, Dalian, China; 4 Department of Microecology, Dalian Medical University, Dalian, China; 5 College of Pharmacy, Dalian Medical University, Dalian, China

**Keywords:** Bifidobacteria, AD, Aβ, Gut microflora

## Abstract

**Background:**

Alzheimer’s disease (AD), being a complex disorder, is affected either by genetic or environmental factors or both. It is observed that there is an excessive accumulation of amyloid β (Aβ) in the extracellular space of the brain. AD is the first neurodegenerative disease in the elderly, and so far there is no effective treatment. In recent years, many studies have reported that Alzheimer’s disease has a relationship with gut microflora, indicating that regulating gut microbiota could offer therapeutic intervention for AD. This study explored the effect *Bifidobacteria* has in averting AD.

**Methods:**

WT and APP/PS1 mice were used for the experiments. The mice were randomly assigned to four groups: WT group, WT + Bi group, AD group (APP/PS1 mouse) and AD + Bi group (*Bifidobacteria-*treated APP/PS1 mouse). Treatment with *Bifidobacteria* lasted for 6 months and mice were prepared for immunohistochemistry, immunofluorescence, Thioflavin S staining, Western blotting, PCR and Elisa quantitative assay.

**Results:**

The results show that after 6 months of treatment with *Bifidobacteria* signiis to be lesficantly reduces Aβ deposition in cortex and hippocampus of AD mice. The level of insoluble Aβ in the hippocampus and cortex of AD+Bi mice was decreased compared with AD mice. Meanwhile, a significant decrease in the level of soluble Aβ in the cortex of AD+Bi mice but not in the hippocampus was observed. The activation of microglia and the release of inflammatory factors were also determined in this study. From the results, *Bifidobacteria* inhibited microglial activation and reduced IL-1β, TNF-α, IL-4, IL-6 and INF-γ release. Altogether, these results implied that *Bifidobacteria* can alleviate the pathological changes of AD through various effects.

## Introduction

Alzheimer’s disease (AD) is a disorder described by progressive cognition and memory impairments (Alzheimer’s Association, 2015). Its pathological features include extracellular amyloid plaque, intracellular nerve fiber bundle tangle and neuron loss, which mainly occur in the elderly with slow memory and cognitive function but progressive loss. In the past few decades, people have conducted extensive research on AD, but its pathogenesis is not clear, and no effective treatment has yet been found. Previous AD studies have focused on brain-related aspects such as the production and degradation of Aβ and abnormal phosphorylation of tau (Wang et al., 2019a; Wang et al., 2019b). In recent years, the gut microbiota has been implicated in the occurrence and progression of AD has become a research hotspot (Vogt et al., 2017).

The gut-brain axis is a multimodal communication system that is connected by both sympathetic and parasympathetic nervous systems, together with hormones that circulate as well as other molecules that regulate the nervous system (Mayer, 2011; Marrone & R, 2019). Recent studies have shown that the function of the gut-brain axis involves intestinal flora and plays a major role in brain and gut communication. It has therefore been reported that the gut-brain axis by extension with microorganism interaction, regulates the immune, gut and central nervous system functions (Cryan & Dinan, 2012). As a result, the microbial communities have become potential diagnostic and therapeutic targets for treating a variety of disorders such as stroke, Parkinson’s disease, Alzheimer’s disease, amyotrophic lateral sclerosis, autism, stroke, depression, and drug addiction (Yarandi et al., 2016; Dong, Xie & Wang, 2019). The enteric nervous system (ENS) and the central nervous system (CNS) even though they are far apart, share many morphological, physiological and pharmacological features. Since bacteria can affect ENS, if they or their messengers can reach the CNS, they will have the same effect on the CNS. A study reported a change in the gut microflora of a bacterial free amyloid β-precursor protein (APP) double transgenic mice model. The amyloid protein deposition in the brains of mice was significantly reduced (Harach et al., 2017). Changes in microbial flora have also been found in other AD mice models and have become more pronounced with increasing age (Shen, Liu & Ji, 2017; Brandscheid et al., 2017). Compared with children/adults and young mice, the composition of gut microbiota in the aged persons and in mice differed. The population of *Bifidobacteria* in the elderly is shown to be less (Lee et al., 2019). Recent studies have also shown that the change of intestinal flora significantly correlates with cognitive behavior. Intestinal flora regulation can affect the cognitive behavior of the host when studied, by using sterile animals, probiotics or antibiotics and fecal flora transplantation (Gareau et al., 2011). Probiotics are active microorganisms that can improve the microecological balance of host and play a beneficial role. *Bifidobacteria* is an anaerobe isolated from the feces of breast-feeding infants by Tissier, a French scholar, in 1899. It has many functions such as antibacterial, anti-aging, immune enhancement, anti-cancer, etc (O’Callaghan & van Sinderen, 2016). It has been reported that the number of *Bifidobacteria* in the gut microbiota is decreased in patients with AD (Cheng et al., 2019).

Based on the above, APP/PS1 double transgenic mice were used as the AD model in this experiment. *Bifidobacteria* were given to mice at 4 months until 10 months of age. The changes in Aβ spot deposition and inflammation in the brain of mice were determined and analyzed. This study provided evidence on the effect of *Bifidobacteria* in AD mice and could provide a new target for the AD treatment.

## Materials & Methods

### Animals

WT and APP/PS1 mice were obtained from Dalian Medical University. The mice were housed in groups (3–5 mice per cage) in polypropylene cages with woodchip bedding, freely had access to food and water and were kept under standard conditions (room temperature of 24 °C, 12 h light/dark cycle and relative humidity of 50–60%). All procedures were subjected to the Institutional Animal Care and Use Committee guidelines and were approved by the Institutional Ethics Committee of Dalian Medical University (L2013011).

### Animal grouping and treatment

The mice were randomly assigned to four groups: WT group, WT + Bi group, AD group (APP/PS1 mouse) and AD + Bi group (*Bifidobacteria-* treated APP/PS1 mouse). .Bifidobacteria (B. longum 1714), was dissolved in water (final concentration: 1 ×10^9^ CFU/mL) right before the treatment and given once a day at 0.2 ml/10 g of body mass (Savignac et al., 2015; Fukuda et al., 2011; Wall et al., 2010). The mice in WT + Bi group and AD + Bi group were given *Bifidobacteria* by gavage. The mice in the control group were given the same volume of water. Samples were taken at 10 months old.

### Euthanization

Mice were deeply anesthesized with pentobarbital (50 mg/kg, i.p) and ensured that it was effective by pinching the tail with tweezers. Any movement in the mice showed that pain could still be felt so enough time was allowed for the anesthesia to fully work before sacrificing the mice. Mice that were not anesthesized were injected with half of the original dose. Mice that survived the study or were excluded were bred for other experiments.

### Immunohistochemistry

Fully anesthesized mice were perfused with cold 4% paraformaldehyde (PFA) in 0.1 M phosphate buffer(PB) with a pH of 7.4. The brains were then removed and post-fixed in 4% PFA overnight at 4 °C. Brains were dehydrated in 30% sucrose in PB, embedded in paraffin, and then sliced into sections of 10-µm thickness. The sections were washed three times with PBS and then incubated with 3% H_2_O_2_ for 10 min to block all endogenous peroxidases. After incubation, sections were washed three times with PBS and blocked with 5% bovine serum albumin for 1 h at room temperature (RT). Slides were then incubated with a primary antibody 6E10 (Covance, SIG39320) overnight at 4 °C. Negative control was included by adding PBS instead of the primary antibody. The sections were again washed and incubated with mouse IgG secondary antibody at room temperature for 1 h, then stained with diaminobiphenylamine (DAB) to develop the color. The reaction of the DAB solution was terminated by submerging the slide in PSB. The slides were then dehydrated in graded ethanol series (50, 75, 90, 100%) for 5 min each and then cleared in xylene for 2 min. Finally, they were mounted with coverslips and imaged with a Leica microscope.

### Immunofluorescence

For the immunofluorescence staining, slices were washed with PBST (0.01 M sodium phosphate buffer of pH 7.4, containing 0.3% (v/v) Triton X-100) and blocked with 5% bovine serum albumin for an hour at room temperature (RT). After that slices were incubated overnight at 4 °C using the following primary antibodies: Iba-1(Wako,019-19741). Negative control was included by adding PBS instead of the primary antibody. Slices were then washed in PBS followed by the addition of DAPI and the respective secondary antibody: goat anti-rabbit Alexa Fluor 488 (1:500). Slices were incubated in the dark for 2 h at RT and then washed with PBS. Images were collected using a Leica Microscope.

### Thioflavin S staining

After the sections were washed three times with PBS, they were immersed in the ThT solution (1 g of Th S in 100 mL distilled water) for 20 min in the dark. Images were collected using a Leica Microscope.

### Western blot analysis

Protein was extracted with a RIPA lysis solution and the protein concentration was determined by BCA protein assay(KeyGEN BioTECH, Jiangsu, China). Protein separation was done on 10% SDS-PAGE gel and transblotted onto PVDF membranes (Millipore, Billerica, MA, USA). The membranes were then blocked in 5% nonfat dried milk for 1 h at RT and were incubated with primary antibodies overnight at 4 °C: Iba-1(Wako,016-20001), β-actin (Abcam,ab179467). Membranes were washed 3 times with TBST and then incubated with the HRP-conjugated secondary antibodies for 1 h at room temperature. Protein bands were detected using enhanced chemiluminescence (ECL, Biotool). Semiquantitative analysis was conducted using ImageJ software.

### PCR

Total RNA was extracted with TRIzol (Invitrogen; Thermo Fisher Scientific, Inc.). The RNA extracts were converted to cDNA using the One-Step RT-PCR System with the qualified total RNA as the template. The primers used were as follows:

IL-1β Forward 5′-CAGGACAGGTATAGATTCTTTCCTTT-3′

IL-1β Reverse 5′-ATGGCAACTGTTCCTGAACTCAACT-3′

TNF-α Forward 5′-CGTCAGCCGATTTGCTATCT-3′

TNF-α Reverse 5′-CGGACTCCGCAAAGTCTAAG-3′

IFN-γ Forward 5′-AGCGGCTGACTGAACTCAGATTGT-3′

IFN-γ Reverse 5′-GTCACAGTTTTCAGCTGTATAGGG-3′

IL-4 Forward 5′-ACAGGAGAAGGGACGCCAT-3′

IL-4 Reverse 5′-GAAGCCCTACAGACGAGCTCA-3′

IL-6 Forward 5′-TAGTCCTTCCTACCCCAATTTCC-3′

IL-6 Reverse 5′-TTGGTCCTTAGCCACTCCTTC-3′

GAPDHForward 5′-TCACCACCATGGAGAAGGC-3′

GAPDH Reverse 5′-GCTAAGCAGTTGGTGGTGCA-3′

Amplification proceeded as follows:

IL-1β 94 °C.45′, 60 °C.45′, 70 °C.60′, 33 Cycles;

TNF-α 95 °C.30′, 58 ° C.40′, 72 °C.40′, 37 Cycles;

IFN-γ 95 °C.20′, 60 ° C.30′, 72 °C.60′, 30 Cycles;

IL-4 95 °C.20′, 55 ° C.30′, 72 °C.60′, 35 Cycles;

IL-6 95 °C.20′, 60 ° C.30′, 72 °C.60′, 35Cycles;

GAPDH 94 °C.45′, 60 ° C.45′, 70 °C. 60′, 24 Cycles

### ELISA quantitative assays for Aβ42

Aβ1-42 in brain tissue was quantitatively estimated using the ELISA kit. The procedure was done in accordance with the protocol provided by the manufacturer (KHB3441, Invitrogen).

### Statiscal analysis

Data are presented as mean ±SEM. Differences between groups were determined by student *t*-test or a two-way analysis of variance (ANOVA), significant effects were evaluated with least signifificant difference (LSD) post hoc test. p-Value <0.05 was considered as the significance level for all analyses (* *p* < 0.05, ** *p* < 0.01;# *p* < 0.05, ## *p* < 0.01;### *p* < 0.001)

## Results

### *Bifidobacteria* reduces Aβ deposition in the brain of AD mice

**Figure 1:**
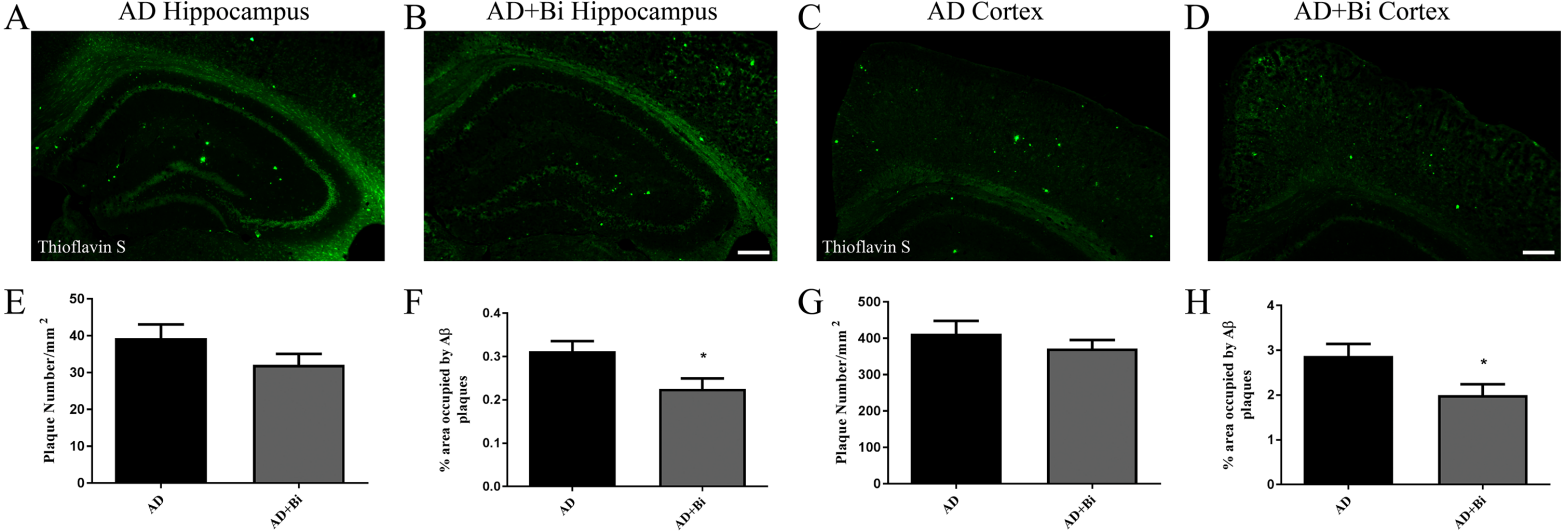
Bifidobacteria reduces insoluble Aβ plaques in the brains of AD mice. Representative images of the hippocampal (A–B) and cortical (C–D) regions of the brains showing stained insoluble Aβ in Bifidobacteria-treated AD mice and AD mice. Scale bar = 100 µm. Quantification and analysis of insoluble Aβ plaques in the hippocampal (E–F) and cortical (G–H) regions of indicated groups. ^∗^*P* < 0.05; *n* = 6.

The deposition of β-amyloid (Aβ) in the brain has always been a key factor in the study of the pathological mechanism of AD (Honjo, Black & Verhoeff, 2012). As the disease progresses, Aβ aggregates into insoluble clusters or plaques in the AD brain. The extent of plaque formation in the cortical and hippocampus has a direct relation with learning and memory disorders in AD (Jahn, 2013). To examine how *Bifidobacteria* affect the formation of plaque in the brain, the levels of Aβ plaques were observed in mice. Brain sections were stained with Thioflavin S (ThS) and the insoluble β-sheet-rich Aβ plaques were observed. As there was no Aβ deposition in the brain of WT and WT+Bi mice, the size and number of Aβ plaques in AD and AD+Bi mice were quantified by stereological analyses in the cortex (Figs. 1C–1D) and hippocampus (Figs. 1A–1B). The results of immunohistochemistry showed that the percentage of Thioflavin-S positive Aβ deposition in the hippocampus (*P* = 0.0270) and cortex (*P* = 0.0360) was significantly reduced in AD+Bi mice compared with the AD mice (Figs. 1H, 1F). Interestingly, there was no significant change in the number of Aβ in the hippocampus (*P* = 0.1638) and cortex (*P* = 0.3557) in AD+Bi mice compared with AD mice (Figs. 1E, 1G). This trend is consistent in the cortex and hippocampus, which means that the administration of *Bifidobacteria* will affect the formation of plaque in different brain regions.

**Figure 2:**
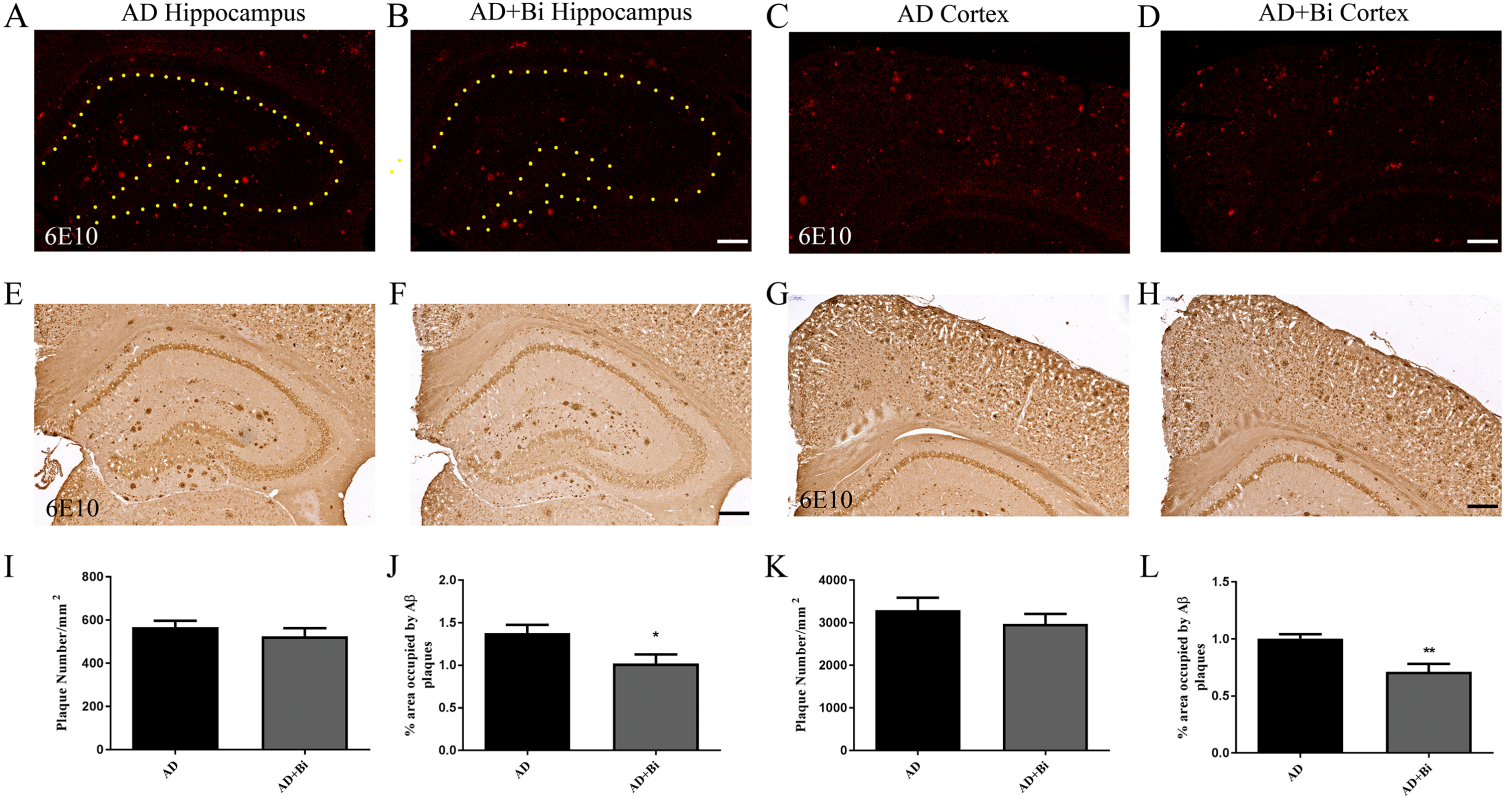
Bifidobacteria reduces total Aβ plaques in the brains of AD mice. Coronal sections of brains showing 6E10-positive Aβ plaques in the hippocampus (A–B; E–F) and cortex (C–D; G–H) of each group. Scale bar = 200 µm.Quantified 6E10-positive Aβ plaques in the hippocampus (I–J) and cortex (K–L) of AD mice. ^∗^*P* < 0.05; *n* = 6.

Aβ plaques occur either as dense-core plaques or diffuse plaques. There is a difference between the dense-core plaques and the diffuse plaques, in that ThS is only able to stain dense-core plaques, and this is as a result of their aggregated β-sheet structure (Rak et al., 2007; Yang et al., 2017). Previous clinical studies have shown that an early pathological sign of AD is depicted by ThS-negatively diffused A β plaques(Price & Morris, 1999). 6E10 was used to detect Aβ (both dense core and diffuse plaques) in the cortex and hippocampus (Figs. 2C–2D; 2G–2H and Figs. 2A–2B; 2E–2F). The results of immunohistochemistry showed that compared with AD mice, the percentage of 6E10 positive Aβ deposition was significantly reduced in the hippocampus (*P* = 0.0326) and cortex (*P* = 0.006) of AD+Bi mice (Figs. 2J, 2L). Similarly, the number of Aβ did not change significantly in the hippocampus (*P* = 0.4044) and cortex (*P* = 0.4145) in AD+Bi mice compared with AD mice (Figs. 2I, 2K).

**Figure 3:**
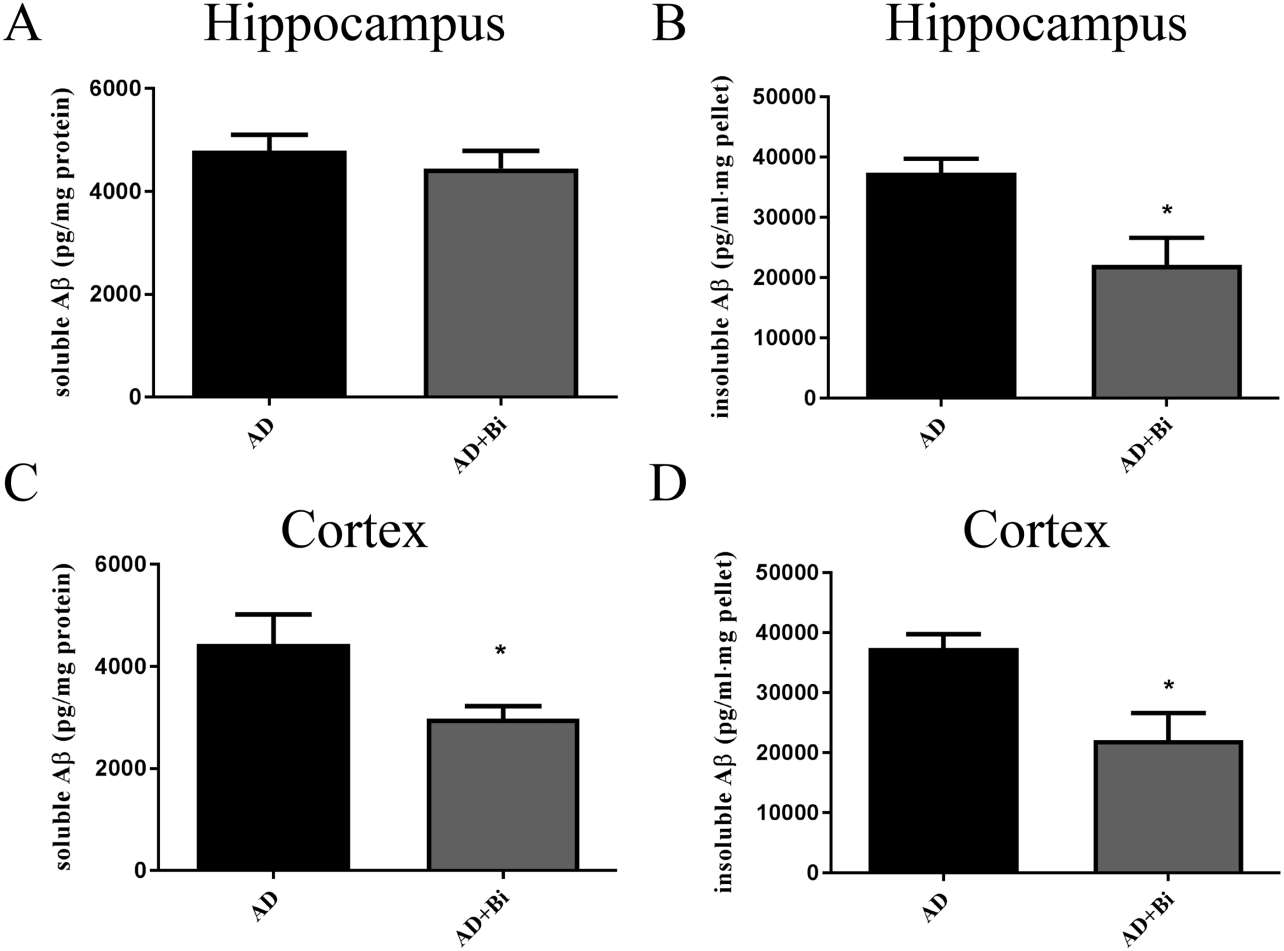
Bifidobacteria decrease insoluble and soluble Aβ in the brains of AD mice. Sandwich ELISA of the soluble Aβ and insoluble Aβ fractions from the hippocampal (A–B) and cortical (C–D) lysates of AD and Bifidobacteria-treated AD mice. Six brain lysates per group were analyzed. ^∗^*P* < 0.05.

Levels of insoluble and soluble Aβ in the cortex and hippocampus were analyzed separately. The use of sandwich enzyme-linked immunosorbent assay (ELISA) was employed to measure Aβ levels. Brain tissues from the cortex and hippocampus were dissolved in RIPA (soluble fraction) and guanidine (insoluble fraction) lysis buffers. As we can see in the results, we found that the level of insoluble Aβ in the hippocampus (*P* = 0.0238) and cortex (*P* = 0.0128) of AD+Bi mice decreased compared with AD mice which are similar observations in the ThS-staining results (Fig. 3 B and D). Meanwhile, a significant decrease was observed in the level of soluble Aβ in the cortex (*P* = 0.0414) of AD+Bi mice (Fig. 3C). On the contrary, the level of soluble Aβ in the hippocampus (*P* = 0.4718) of AD+Bi mice did not decrease compared with AD mice (Fig. 3A).

**Figure 4:**
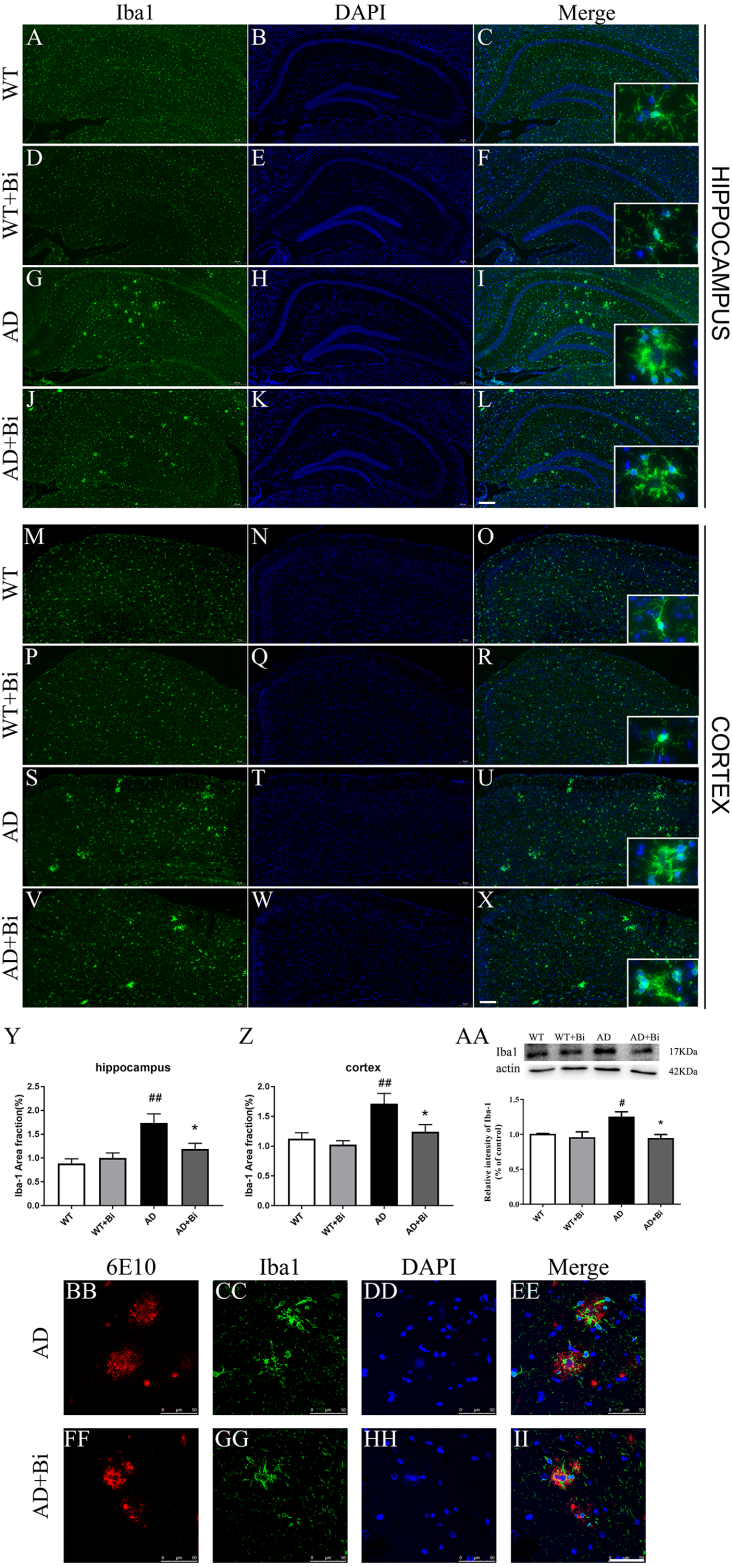
Bifidobacteria inhibit the activation of microglia in AD mice. Coronal sections of brains showing Iba1-positive immuno-stained microglia in the hippocampus (A–L) and cortex (M–X). Scale bar = 200 µm. (Y–Z) Positive area % of Iba-1 immunostaining detected by immunofluorescence. (AA) Representative immunoblot and densitometric analysis showing Iba1 protein expression level. Here, the bands were normalized to β-actin. (BB–II) Bifidobacteria treatment alleviated the activations of microglia around Aβ plaque. Scale bar = 50 µm. *compared with AD group, ^∗^*P* < 0.05; # compared with WT group; *n* = 6.

### *Bifidobacteria* inhibit the activation of microglia in AD mice

Microglia plays an important role in the neuroinflammatory pathogenesis of AD(Lee et al., 2014). When microglia are activated they can induce and maintain chronic inflammatory states, and lead to neuronal damage as well as neurodegeneration (Zhang et al., 2017). Therefore, we sought to measure whether the *Bifidobacteria* can inhibit the activation of microglia. The expression of Iba1 (one of the markers for microglia) was determined. As represented in Figs. 4A–4Z, treatment with Bifidobacteria reduced the percentage of the area immunostained with Iba-1 in the hippocampus (*P* = 0.0026; Fig. 4Y) and cortex (*P* = 0.0016; Fig. 4Z) of AD mice. To further confirm the IF findings, the protein expression level of Iba1 in the hippocampus was determined with WB. Consistent with the IF results, the protein level of Iba1 decreased in *Bifidobacteria* treated AD mice (*P* = 0.0144; Fig. 4AA). Immunostaining also revealed that Bifidobacteria treatment alleviate the activation of microglia around Aβ plaques (Figs. 4BB–4II). It is suggested that inhibition of microglial activation may be one of the protective mechanisms of *Bifidobacteria* on memory impairment in AD mice.

### *Bifidobacteria* inhibit neuroinflammation in AD mice

**Figure 5:**
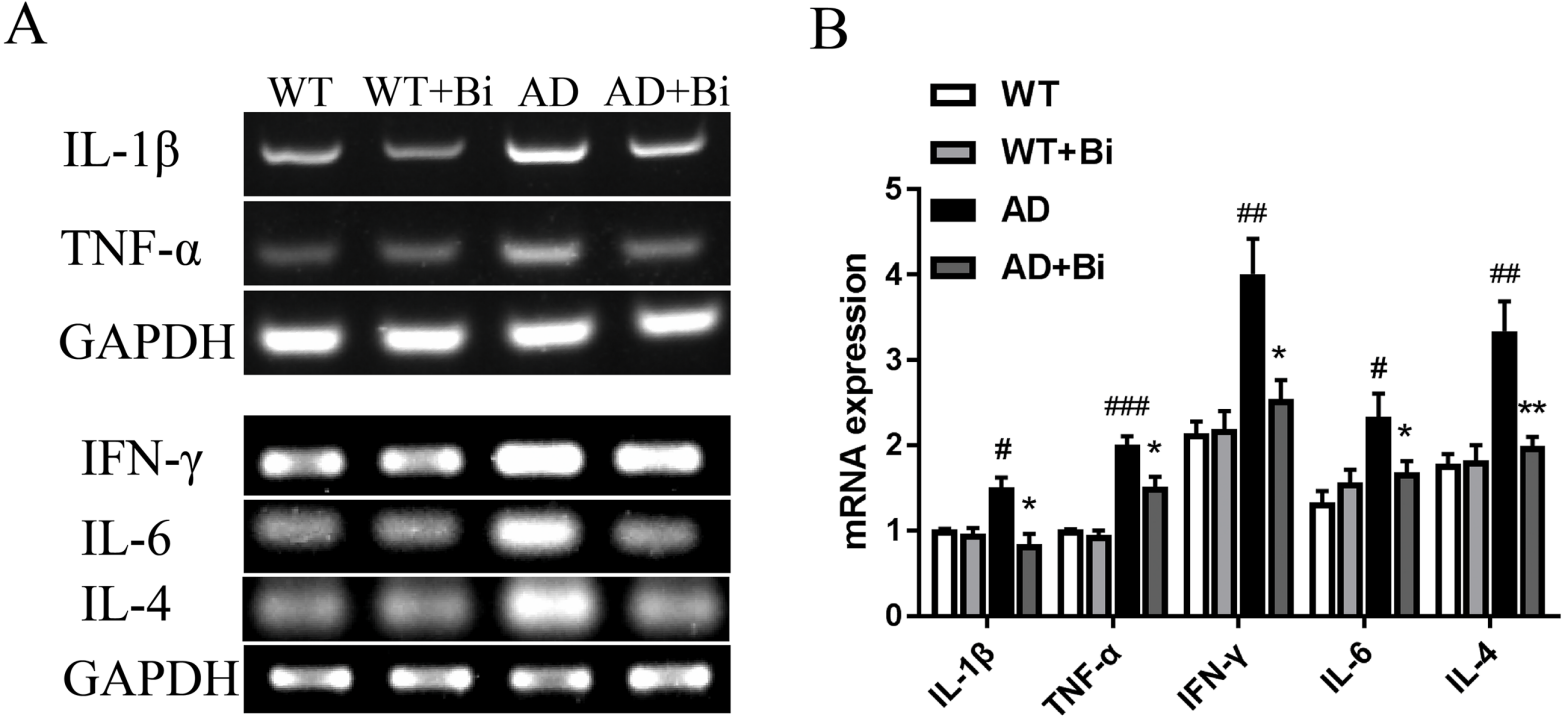
Bifidobacteria inhibit neuroinflammation in AD mice. Representative micrographs (A) and densitometric analysis (B) of IL-1β, TNF-α, IFN-γ , IL-4 and IL-6 mRNA expression levels. Here, bands were normalized to GAPDH. *compared with AD group, ^∗^*P* < 0.05, ^∗∗^*P* < 0.01; ^#^ compared with WT group, ^#^*P* < 0.05, ^##^*P* < 0.01, ^###^*P* < 0.001; *n* = 6.

Activated microglia can mediate neuroinflammation in various neurological disorders via producing proinflammatory cytokines such as tumor necrosis factor-α (TNF-α), interleukin-1β (IL-1β), interleukin-6 (IL-6), interleukin-4 (IL-4) and interferon-γ (IFN-γ) (Yan et al., 2019; Na, Jung & Kim, 2012). Therefore, the mRNA expression levels of the pro-inflammatory factors (TNF-α, IL-1β, IL-4, IL-6, IFN-γ) in the hippocampus were detected in each group by PCR. The mRNA levels of these pro-inflammatory factors were increased significantly in AD mice compared to the WT mice. On the other hand, the mRNA levels were reduced in Bifidobacteria-treated AD mice compared with the AD mice (IL-1 β, *P* = 0.0395; TNF-α, *P* = 0.0498; IL-4, *P* = 0.0099; IL-6, *P* = 0.0353; IFN-γ, *P* = 0.0212; Fig. 5). These findings suggested that Bifidobacteria may potentially have an anti-inflammatory effect in the AD condition.

## Discussion

A large number of bacteria colonize the human colon. The most popular among them are the sclerenchyma and Bacteroides, which account for 51% and 48% of the total microbial population respectively (Westfall et al., 2017; Qin et al., 2010). Gut microflora is very important for health. Originally, it was thought that all microbes in the gut were only restricted to colon-specific activities, such as fermentation of carbohydrates, synthesis of vitamins (such as vitamin B and K), and prevent other disease-causing bacteria from attacking the gastrointestinal tract (Salminen et al., 1998; Simon & Gorbach, 1986). Recently, probiotics have shown great potential in preventing and treating many diseases, and their role in diseases of central nervous system diseases have been gradually discovered. The gut-brain axis offers such a dynamic bidirectional neuroendocrine system composed of uninterrupted neural contacts, endocrine indicators as well as immune factors (Burokas et al., 2015; Forsythe, Bienenstock & Kunze, 2014; Bauer, Huus & Finlay, 2016). Many linked hormones and biochemical pathways exist that link the condition of gut microflora to some brain functions, giving probiotics a great potential to be used as therapeutic agents for neurodegenerative disorders. It has recently been reported that changes in gut microbial content can modify normal brain function, resulting in conditions such as anxiety, depression, and cognitive impairment (Zhu et al., 2017a; Zhu et al., 2017b; Foster & McVey Neufeld, 2013). In addition, there are reports showing that the make-up of gut microflora is significantly associated with aging. Hence, the diversity of gut microflora will be reduced in an aging intestinal tract (Biagi et al., 2010; Cheng et al., 2013; Claesson et al., 2011).

AD is the most common form of dementia in the elderly, found in about 60%–80% of all patients with dementia (Beckman et al., 2019). Some epidemiological and preclinical pieces of evidence show that changes in intestinal microflora are linked to AD pathogenesis and development. For instance, the composition of microbial colonies in AD patients was different from that in the control group (Vogt et al., 2017). A study reported that bacteria phyla or strains in feces of 5xFAD Alzheimer’s mice can be distinguished from that of wild-type littermate (Brandscheid et al., 2017). Several gut bacteria, such as *E. coli*, *Salmonella* and *Citrobacter*, produce Aβ (O’Toole, Kaplan & Kolter, 2000; Zhou et al., 2012). Exposure to microbial amyloid may cause misfolding of amyloid in the brain (Cerovic, Forloni & Balducci, 2019). Gv-971, a new anti-AD drug recently launched in China, can remodel the balance of gut microflora, normalize disordered metabolites to reduce Aβ deposition, and ultimately improve cognitive function (Wang et al., 2019a; Wang et al., 2019b). In view of this, probiotics and prebiotics may effectively fight against dementia, which is considered as an important progress in the field of AD.

Alzheimer’s disease has been categorized by four distinct hallmarks. These include Aβ plaque, tangled nerve fibers, loss of nerve and synapse, and cognitive impairment. At present, the treatment of AD has been the emphasis of much research. Some of these studies are Anti-Amyloid Treatment in Asymptomatic Alzheimer’s (A4) and Dominantly Inherited Alzheimer Network (DIAN), all of which had the aim to prevent or delay the AD onset and progression and other associated outcomes (Yang et al., 2017). In this present study, we hypothesize that *Bifidobacteria* ingestion can regulate a variety of pathological factors, that are critical in AD development. Aβ deposition in the brain is one of the pathological hallmarks well-known in AD and is strongly linked with synaptic plasticity impairment in the hippocampus, leading to behavioral abnormalities (Meng, Wang & Li, 2019). Our results confirmed the effect of *Bifidobacteria* on the Aβ burden in APP/PS1 double transgenic mice. After 6 months of treatment with *Bifidobacteria*, the pathological brains of *Bifidobacteria* treated APP/PS1 mice were evaluated. A significant reduction in Aβ plaque burden was observed in the hippocampus and cortex (Figs. 1 and 2). Also, the Aβ lowering properties of *Bifidobacteria* were assessed by measuring soluble and insoluble Aβ species in the *Bifidobacteria*-treated and -untreated APP/PS1 mice. Aβ (a 4 kDa peptide with 40- and 42-amino acid residue peptides as the predominant species) is produced via sequential amyloid precursor protein (APP) cleavage by the enzymes β-and γ-secretase (Katayama, 2019; Ferreira et al., 2015). Biochemical studies have shown that Aβ1-42 is more likely to aggregate into amyloid fibers and other combinations than Aβ1-40 (Jarrett & Lansbury, 1993). Therefore, using ELISA, soluble Aβ1-42 and insoluble Aβ1-42 were detected in the brain. As reported earlier, treatment with *Bifidobacteria* in APP/PS1 mice decreased the levels of insoluble and soluble A β1-42 in the cortex, while *Bifidobacteria* showed a significant reduction in the insoluble Aβ1-42 in the hippocampus and no effect on soluble Aβ1-42 (Fig. 3).

Taken together, we can see that *Bifidobacteria* reduce significantly, the pathological deposition of Aβ in APP/PS1 mice. Although we did not conduct behavioral tests, we have reason to believe that *Bifidobacteria* could recover learning and memory impairments in APP/PS1 mice, given the important role of Aβ in AD. *Bifidobacteria* breve A1, when orally administered to AD model mice (intracerebroventricularly administered Aβ25-35) daily by gavaging 1 ×10^9^ organisms in 0.2ml can repress the expression of inflammation-related and immune-reactive genes (Kobayashi et al., 2017) and improved the cognitive function of participants with mild cognitive impairment (MCI) (Kobayashi et al., 2018). In a clinical trial of 52 subjects in Iran, the subjects took 200 ml/day of probiotic milk containing *Lactobacilli* and *Bifidobacteria* (2 ×10^9^ CFU/g for each) every day for 12 consecutive weeks. Their results showed that consuming the probiotics for 12 weeks had a positive effect on some metabolic states of AD patients as well as improved their cognitive function. These results conform to our hypothesis (Akbari et al., 2016).

Inflammation in the brain is one of the hallmarks of AD and is initiated in the central nervous system (Zeng et al., 2018). Numerous studies have reported that Aβ can trigger oxidative stress in the brain, and also activate microglia leading to neuroinflammation and cognitive dysfunction (Kobayashi et al., 2017). Microglia are the resident immune cells in the central nervous system (CNS) (Wang et al., 2016) and are considered to be the important modulators in brain immunity and homeostasis. They play an important role in various nervous system diseases including AD. Microglia activation exerts effects that could be toxic or beneficial depending on their phenotype in the progression of AD. Activated microglia can internalize and degrade Aβ, suggesting that the initial response of microglia may contribute to the elimination of Aβ. However, as the disease progresses, their ability to clear Aβ is considered to be much limited or even reduced (Zhu et al., 2017a; Zhu et al., 2017b). At the same time, the continuous uncontrolled activation of glial cells induced by Aβ plaques still maintains its ability to produce a variety of cytokines and chemokines which lead to neurodegeneration, and thus even hasten the progression of AD (Zhao et al., 2018; Kiyota et al., 2018). The present findings showed that *Bifidobacteria* treatment inhibits the activation of microglial (Fig. 4). Also, examining the expression of IL-1 β, TNF-α, IL-4, IL-6 and IFN-γ to see whether *Bifidobacteria* treatment reduces the release of proinflammatory cytokines, the results obtained a significant reduction in the mRNA expression levels of these pro-inflammatory cytokines in *Bifidobacteria* treated APP/PS1 mice compared with the control (Fig. 5).

## Conclusions

In conclusion, this study showed the therapeutic potential in *Bifidobacteria.* Treatment with *Bifidobacteria* can suppress Aβ accumulation and neuroinflammation in APP/PS1 mice. Further studies are therefore needed to elucidate the effect of *Bifidobacteria* on learning and memory and the possible mechanisms involved. However, with *Bifidobacteria* being an inherent bacterium in the intestine, it can be used as a safe and long-term means to fight AD.

##  Supplemental Information

10.7717/peerj.10262/supp-1 Supplemental Information 1 Raw data for Figs. 1–3 and Fig. 5

10.7717/peerj.10262/supp-2 Supplemental Information 2 Raw data for Fig. 4

10.7717/peerj.10262/supp-3 Supplemental Information 3 Raw data for Fig. 4 cortex

10.7717/peerj.10262/supp-4 Supplemental Information 4 Supplemental Figures

## Additional Information and Declarations

### Data Availability

The following information was supplied regarding data availability: The raw measurements are available in the Supplemental Files.
