# Supplementary figures and images for: Treatment with Bifidobacteria can suppress Aβ accumulation and neuroinflammation in APP/PS1 mice
Source: PeerJ. 2020 Oct 28;8:e10262. doi: 10.7717/peerj.10262 (PMC7602682; doi:10.7717/peerj.10262)

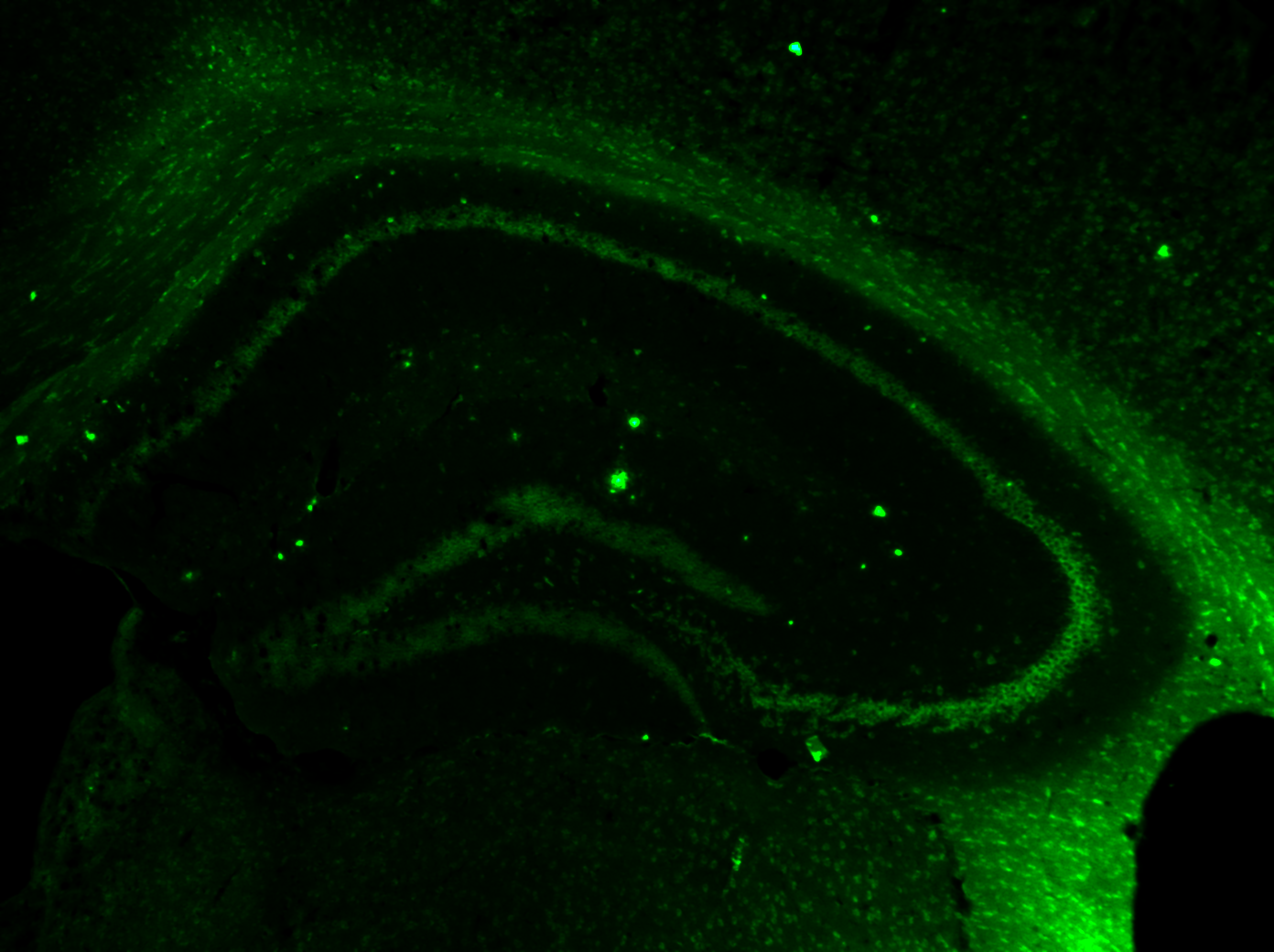

Supplement: Supplemental Information 1 [file peerj-08-10262-s001.zip › raw data1/FIG1/AD hippocampus.png]

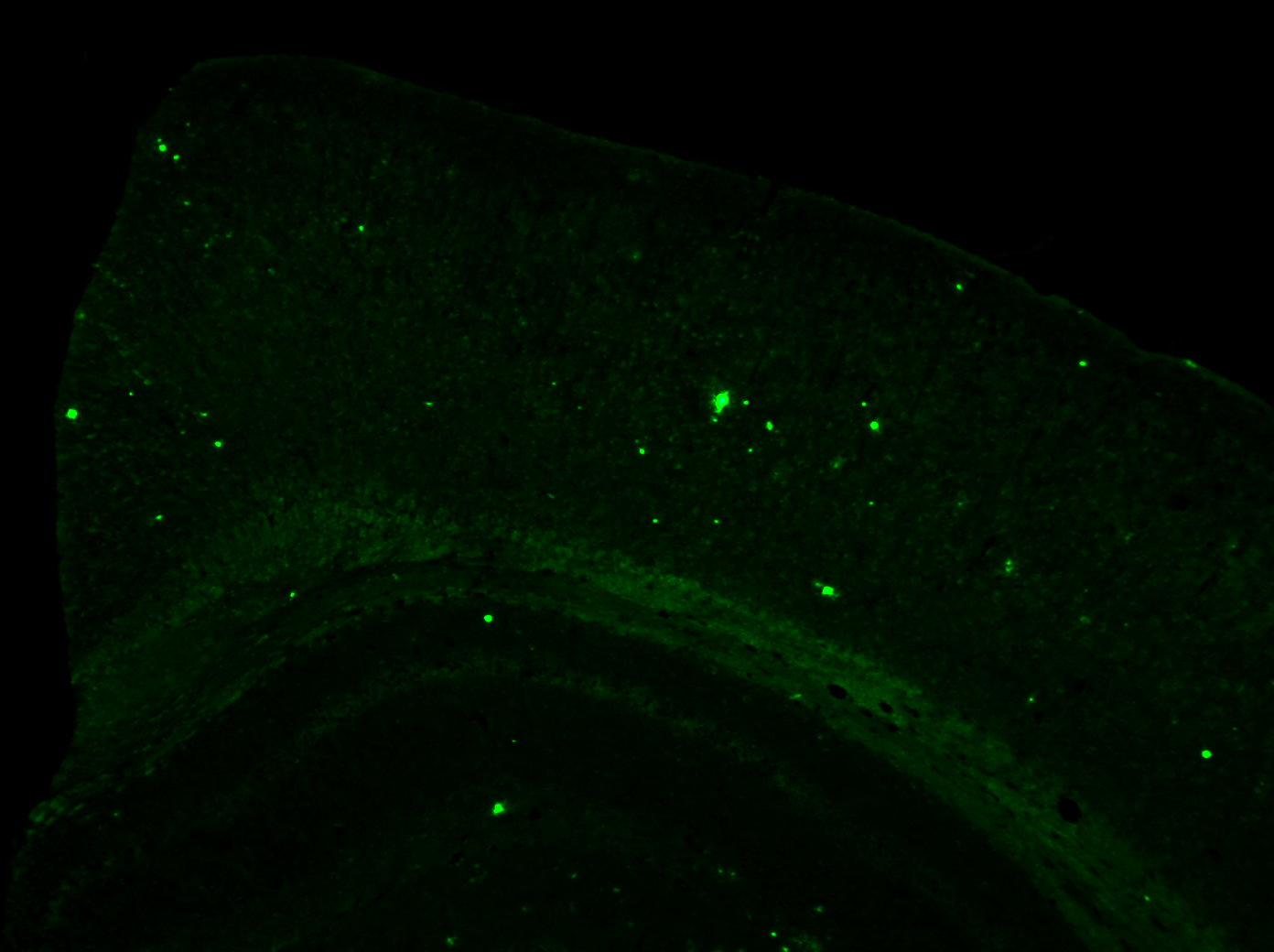

Supplement: Supplemental Information 1 [file peerj-08-10262-s001.zip › raw data1/FIG1/AD cortex .png]

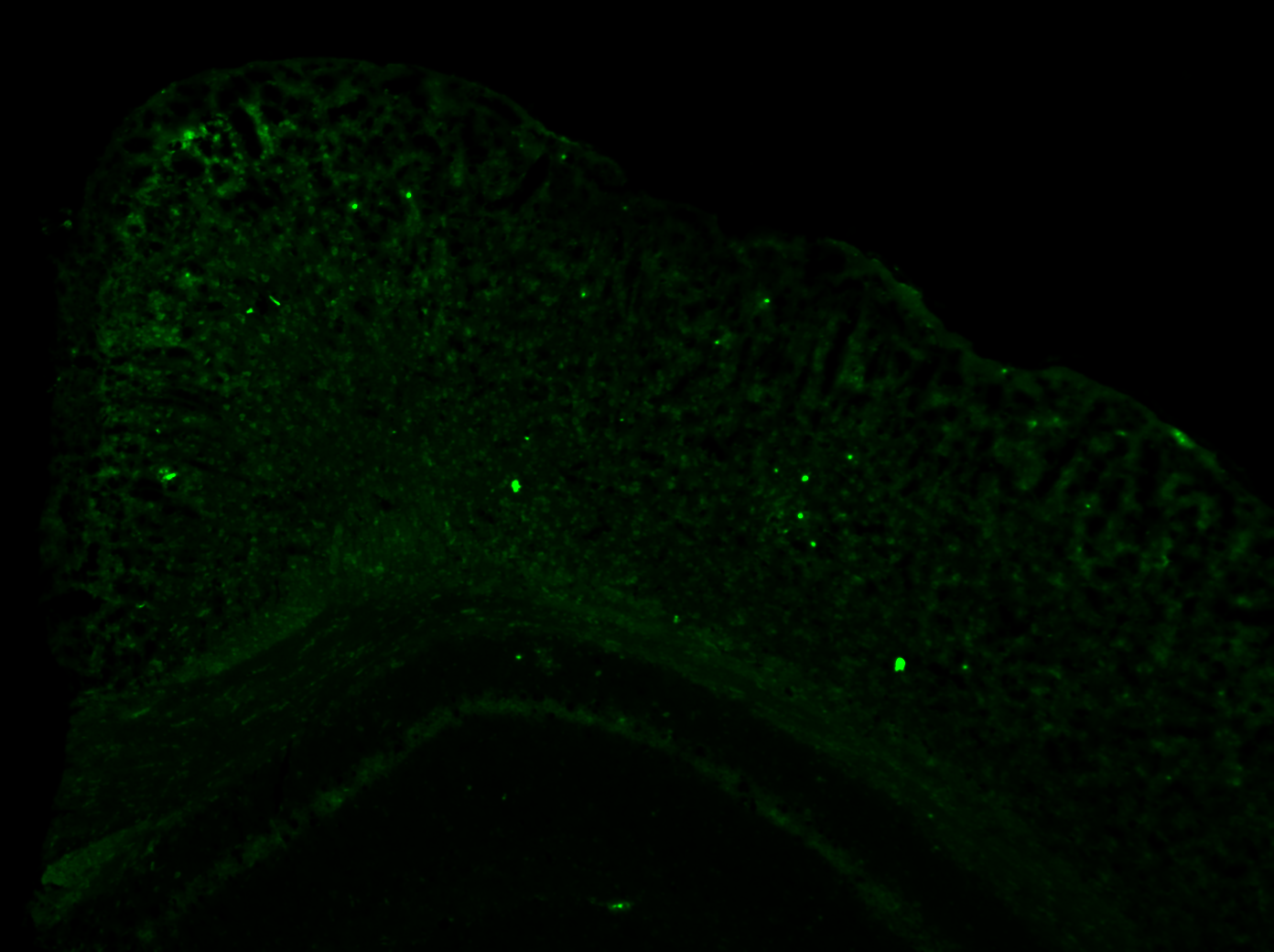

Supplement: Supplemental Information 1 [file peerj-08-10262-s001.zip › raw data1/FIG1/AD+Bi cortex .png]

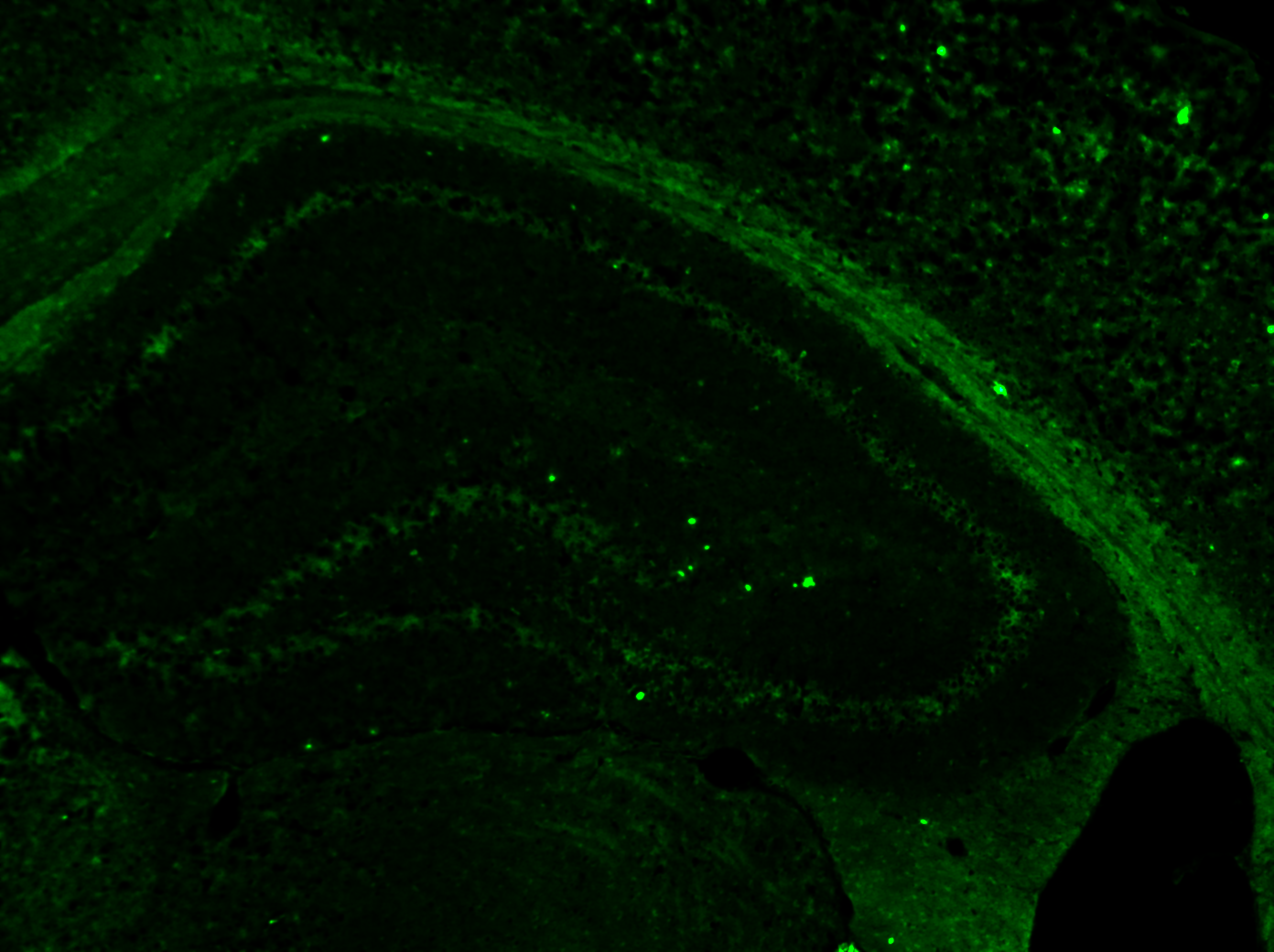

Supplement: Supplemental Information 1 [file peerj-08-10262-s001.zip › raw data1/FIG1/AD+Bi hippocampus.png]

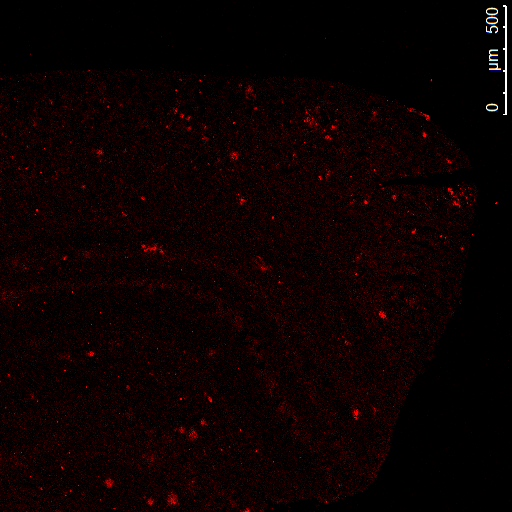

Supplement: Supplemental Information 1 [file peerj-08-10262-s001.zip › raw data1/FIG2/IF/AD+Bi 5X cortex.png]

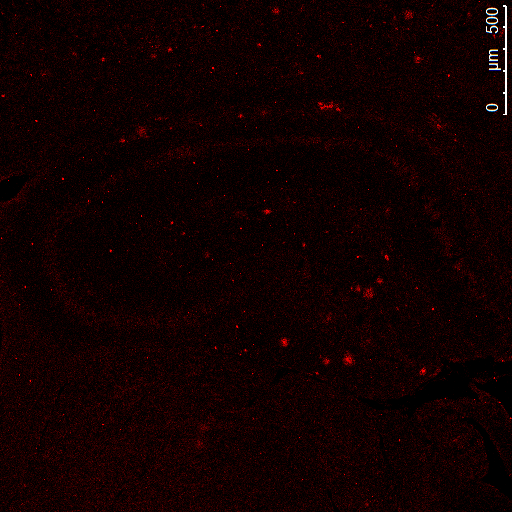

Supplement: Supplemental Information 1 [file peerj-08-10262-s001.zip › raw data1/FIG2/IF/AD+Bi 5X hippocampus.png]

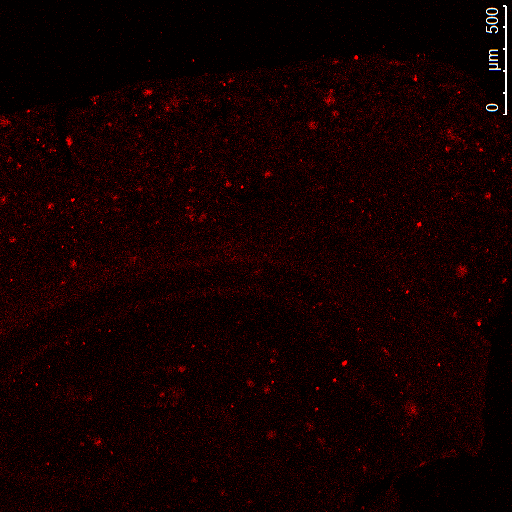

Supplement: Supplemental Information 1 [file peerj-08-10262-s001.zip › raw data1/FIG2/IF/AD-1 5X Cortex.png]

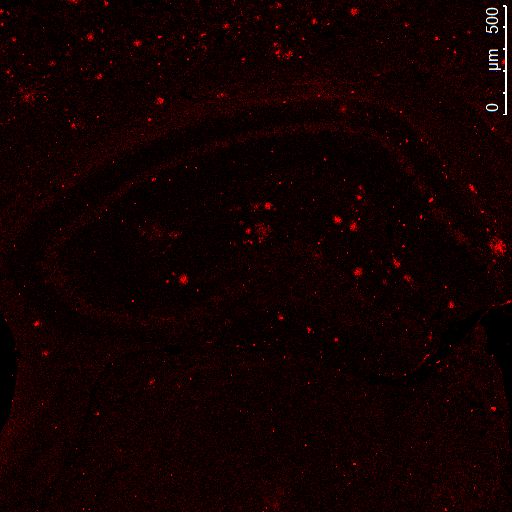

Supplement: Supplemental Information 1 [file peerj-08-10262-s001.zip › raw data1/FIG2/IF/AD-1 5X hippocampus.png]

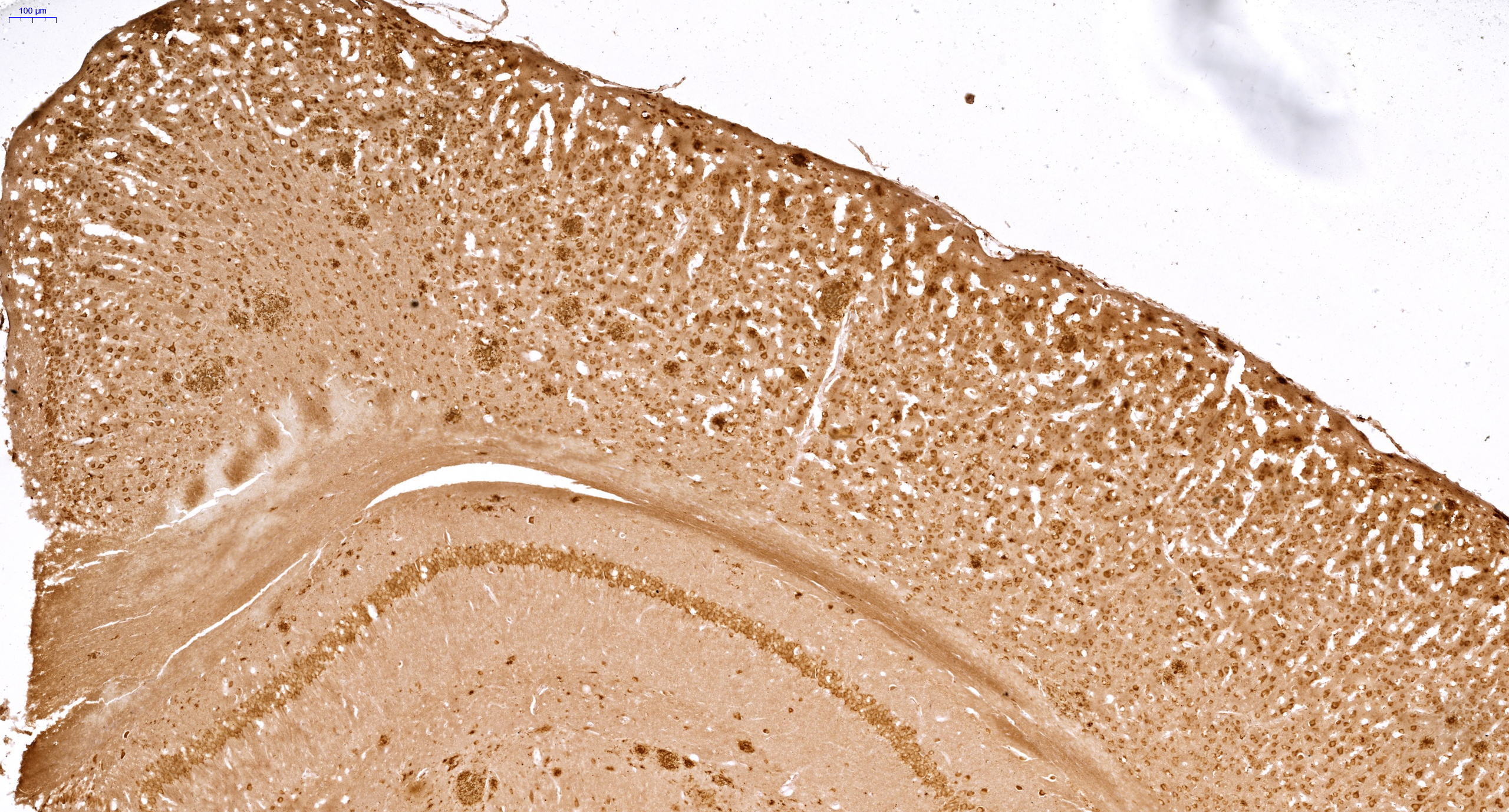

Supplement: Supplemental Information 1 [file peerj-08-10262-s001.zip › raw data1/FIG2/IH/AD cortex10.0x .png]

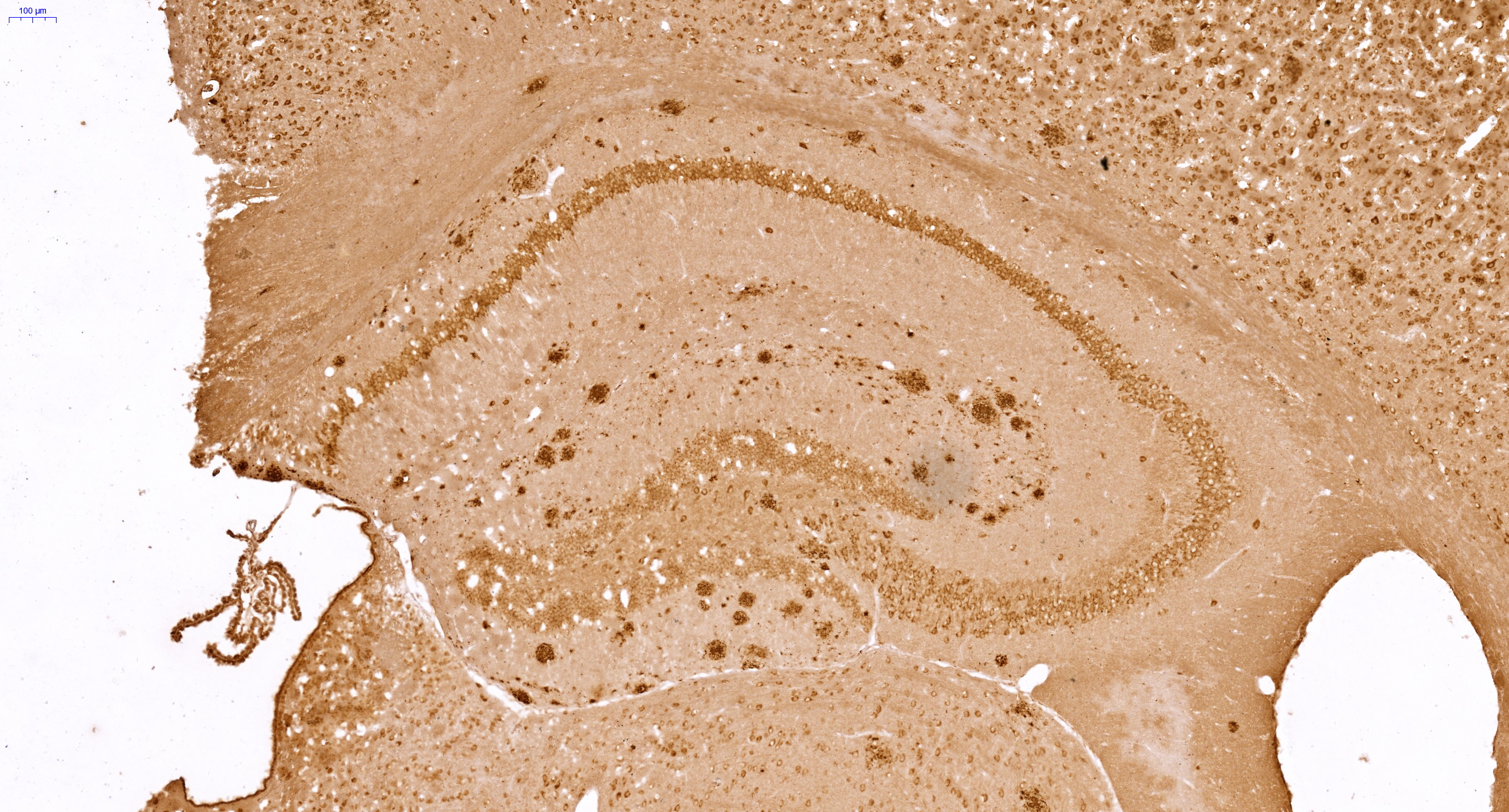

Supplement: Supplemental Information 1 [file peerj-08-10262-s001.zip › raw data1/FIG2/IH/AD hippocampus10.0x.png]

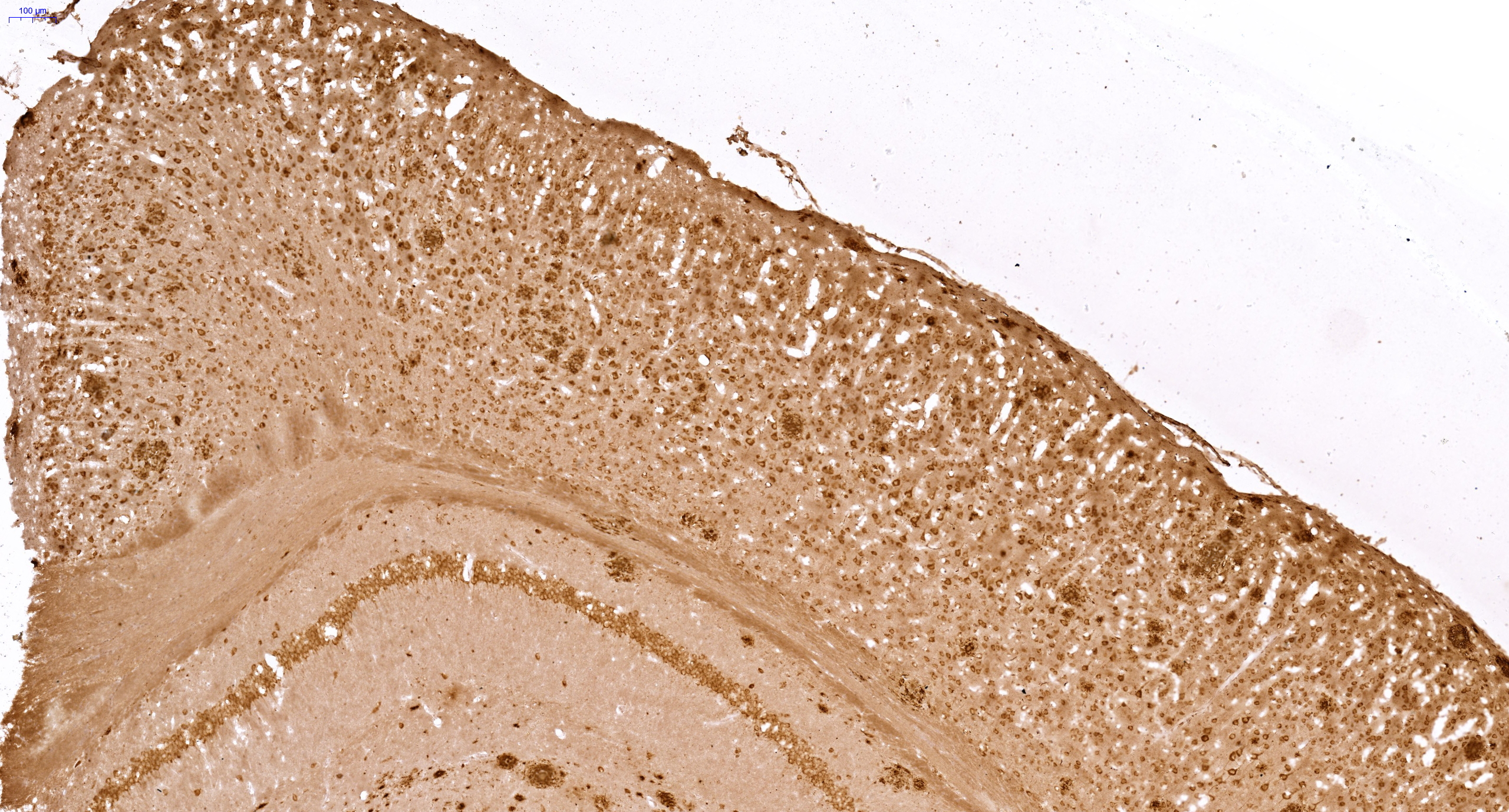

Supplement: Supplemental Information 1 [file peerj-08-10262-s001.zip › raw data1/FIG2/IH/AD+Bi cortex 10.0x .png]

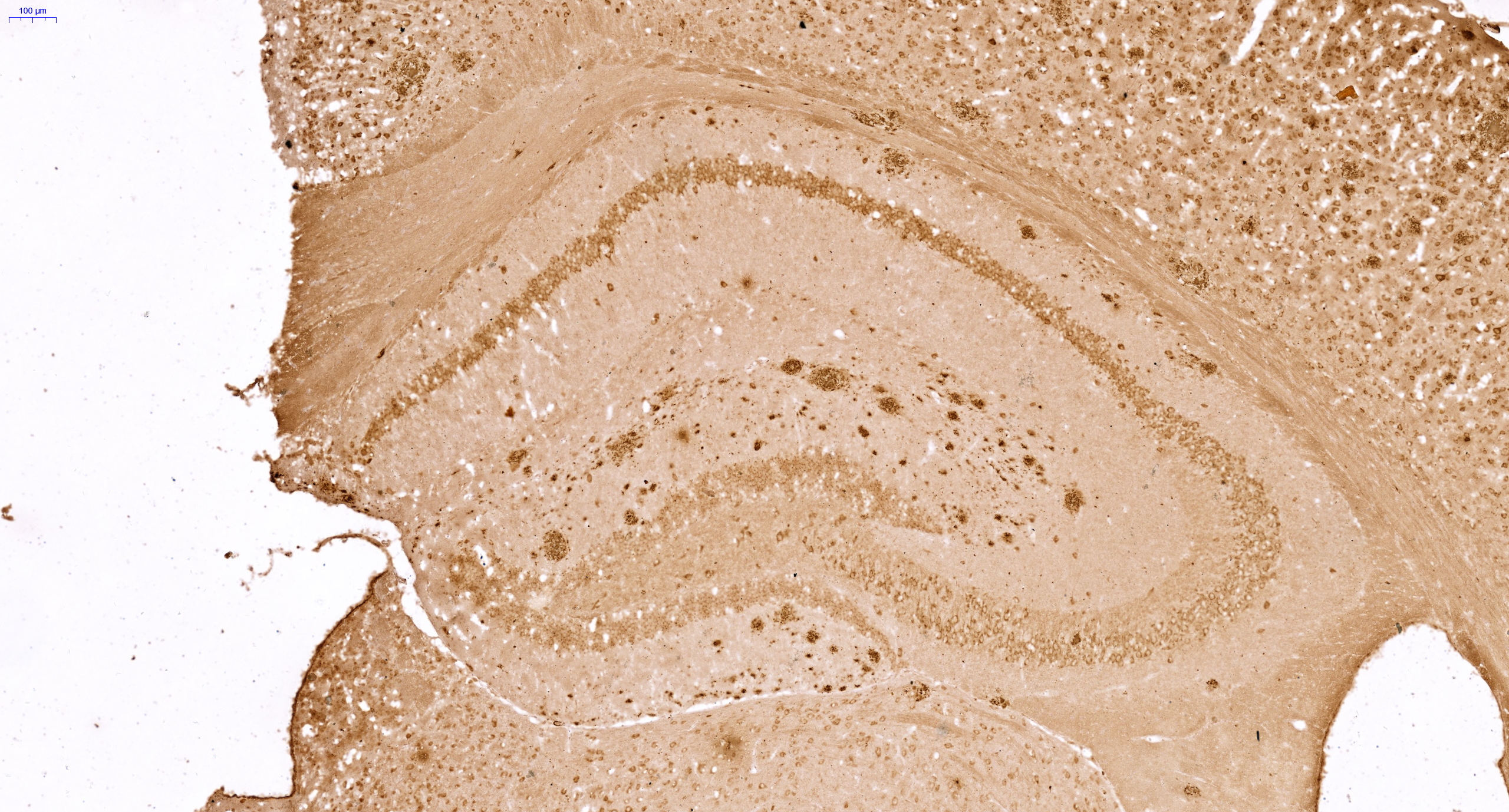

Supplement: Supplemental Information 1 [file peerj-08-10262-s001.zip › raw data1/FIG2/IH/AD+Bi hippocampus 10.0x.png]

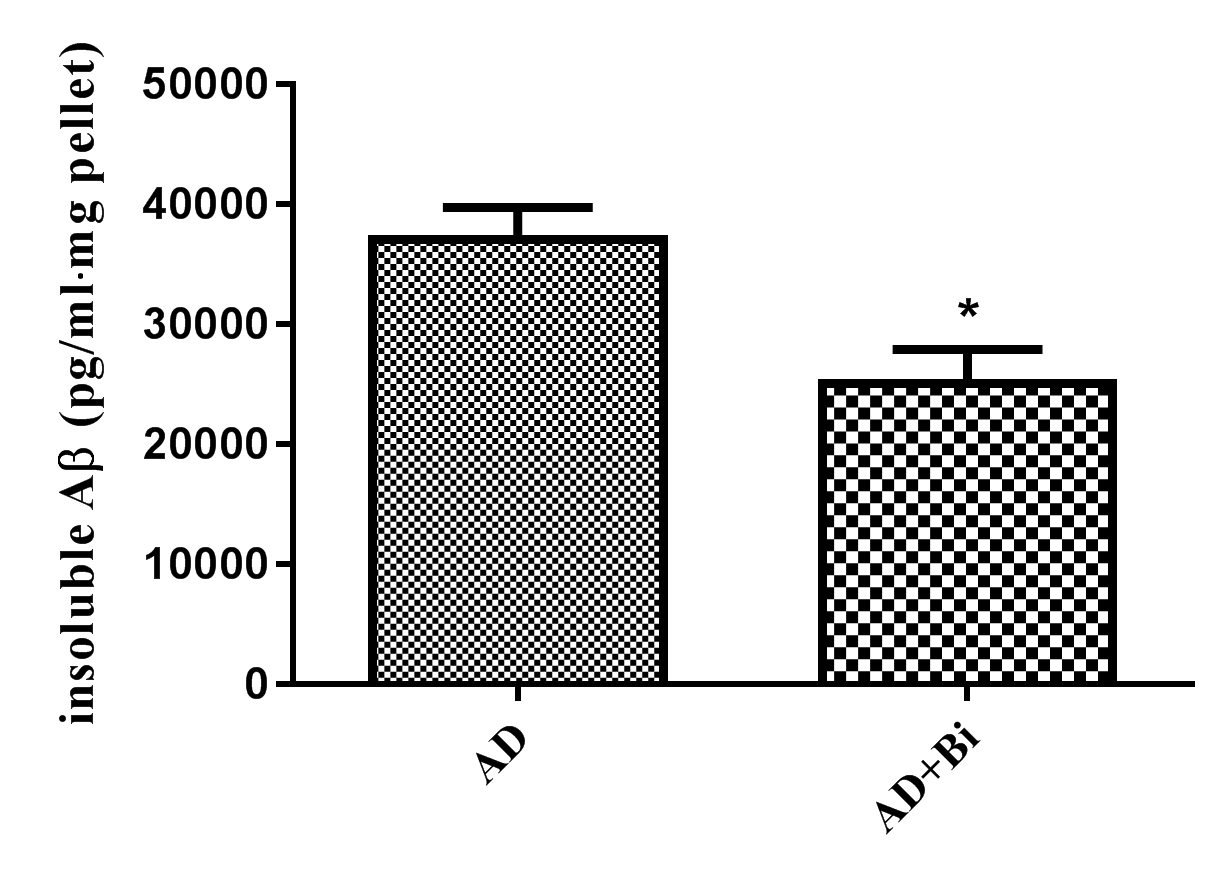

Supplement: Supplemental Information 1 [file peerj-08-10262-s001.zip › raw data1/FIG3/cortex insoluble.png]

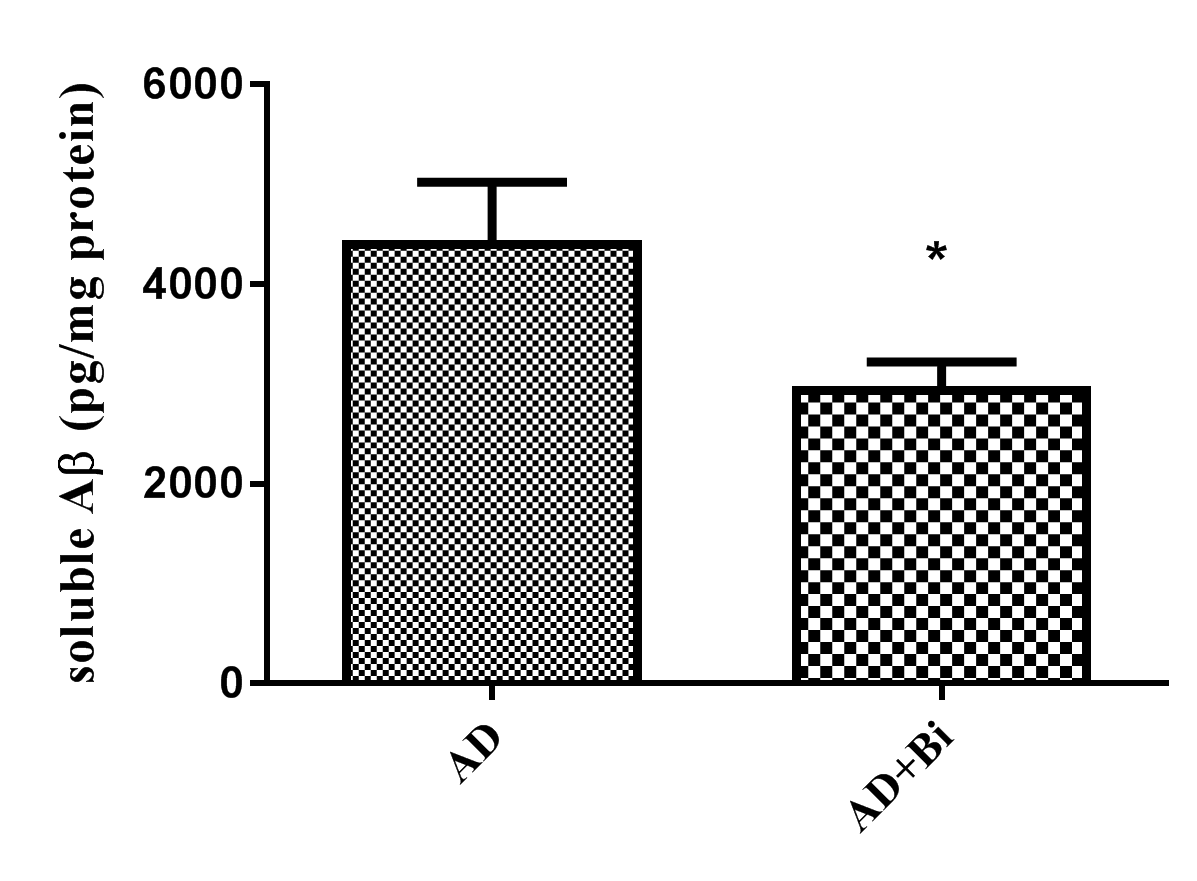

Supplement: Supplemental Information 1 [file peerj-08-10262-s001.zip › raw data1/FIG3/cortex soluble.png]

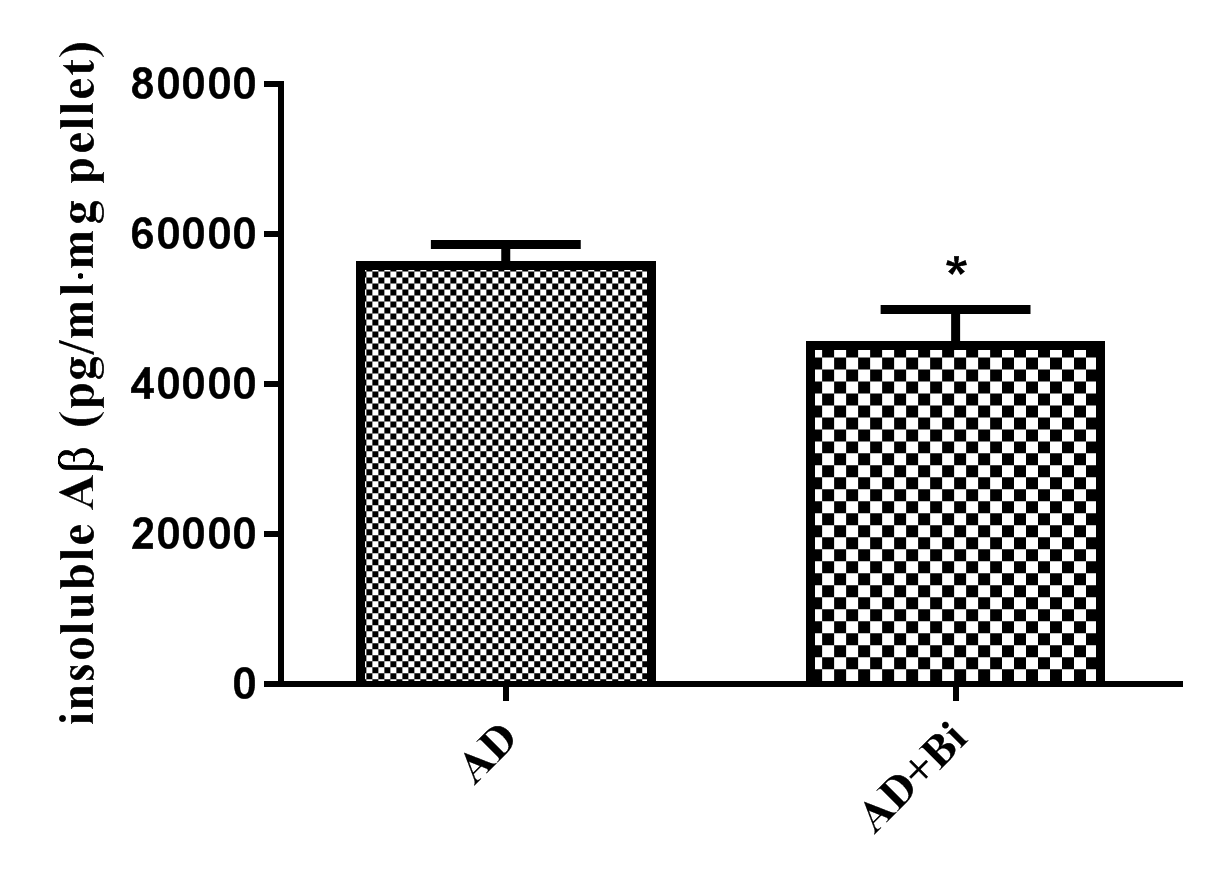

Supplement: Supplemental Information 1 [file peerj-08-10262-s001.zip › raw data1/FIG3/hippocampus insoluble.png]

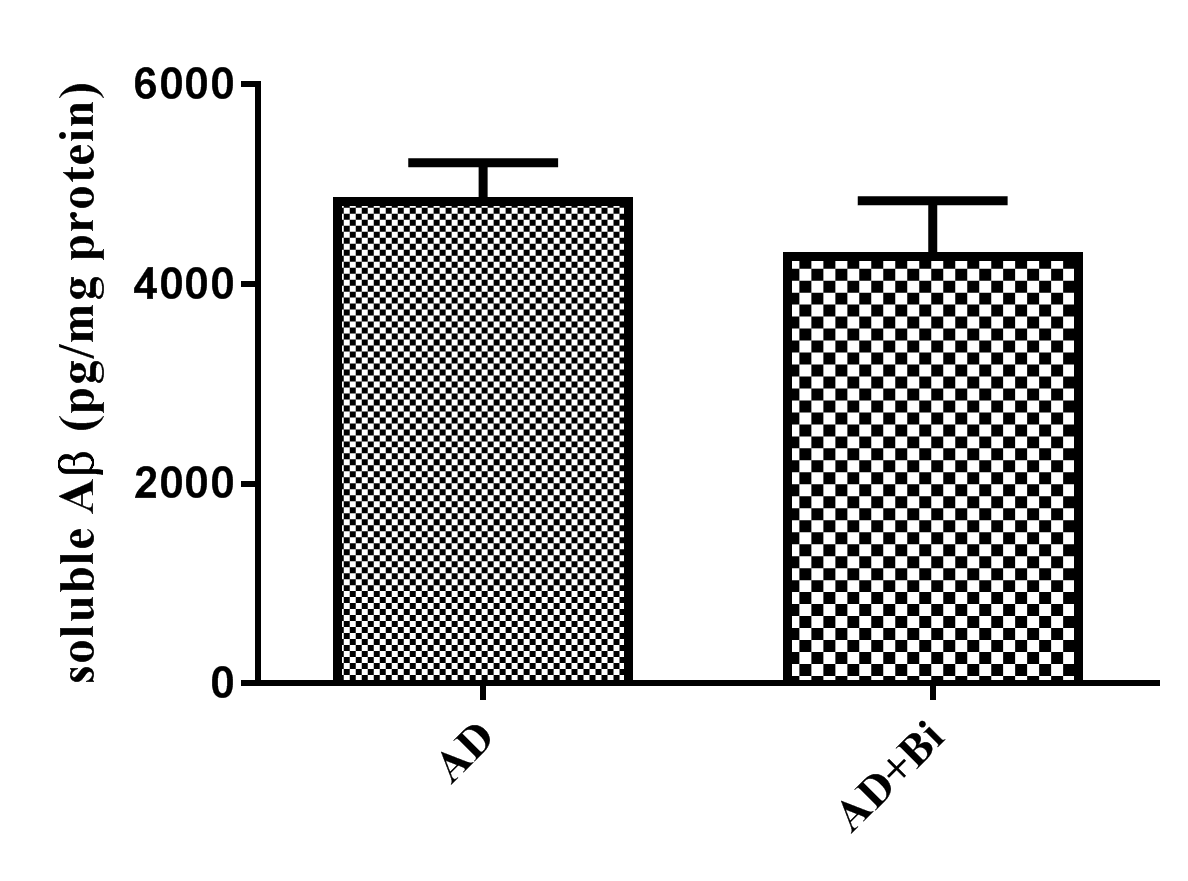

Supplement: Supplemental Information 1 [file peerj-08-10262-s001.zip › raw data1/FIG3/hippocampus soluble.png]

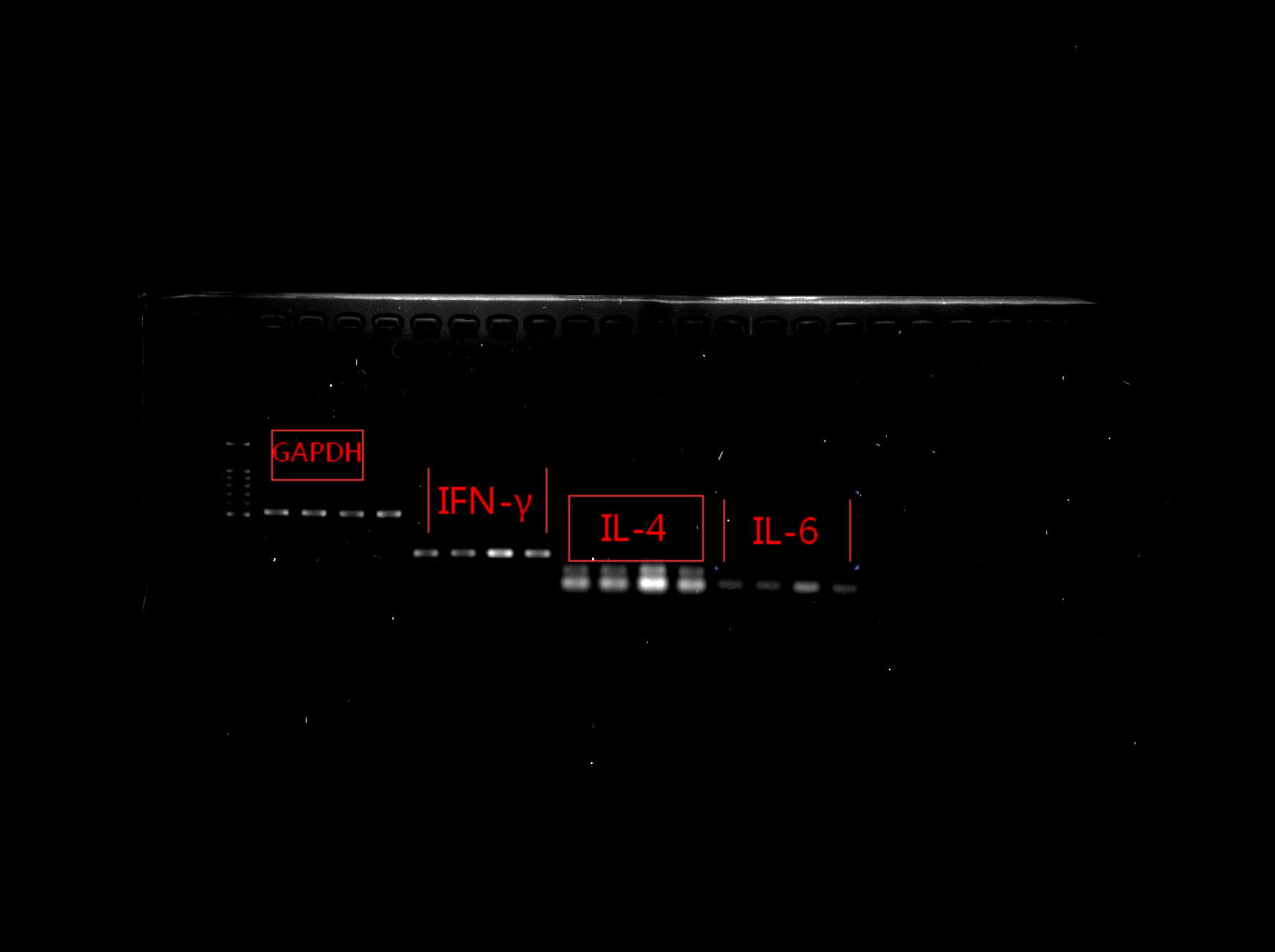

Supplement: Supplemental Information 1 [file peerj-08-10262-s001.zip › raw data1/FIG5/PCR bands.png]

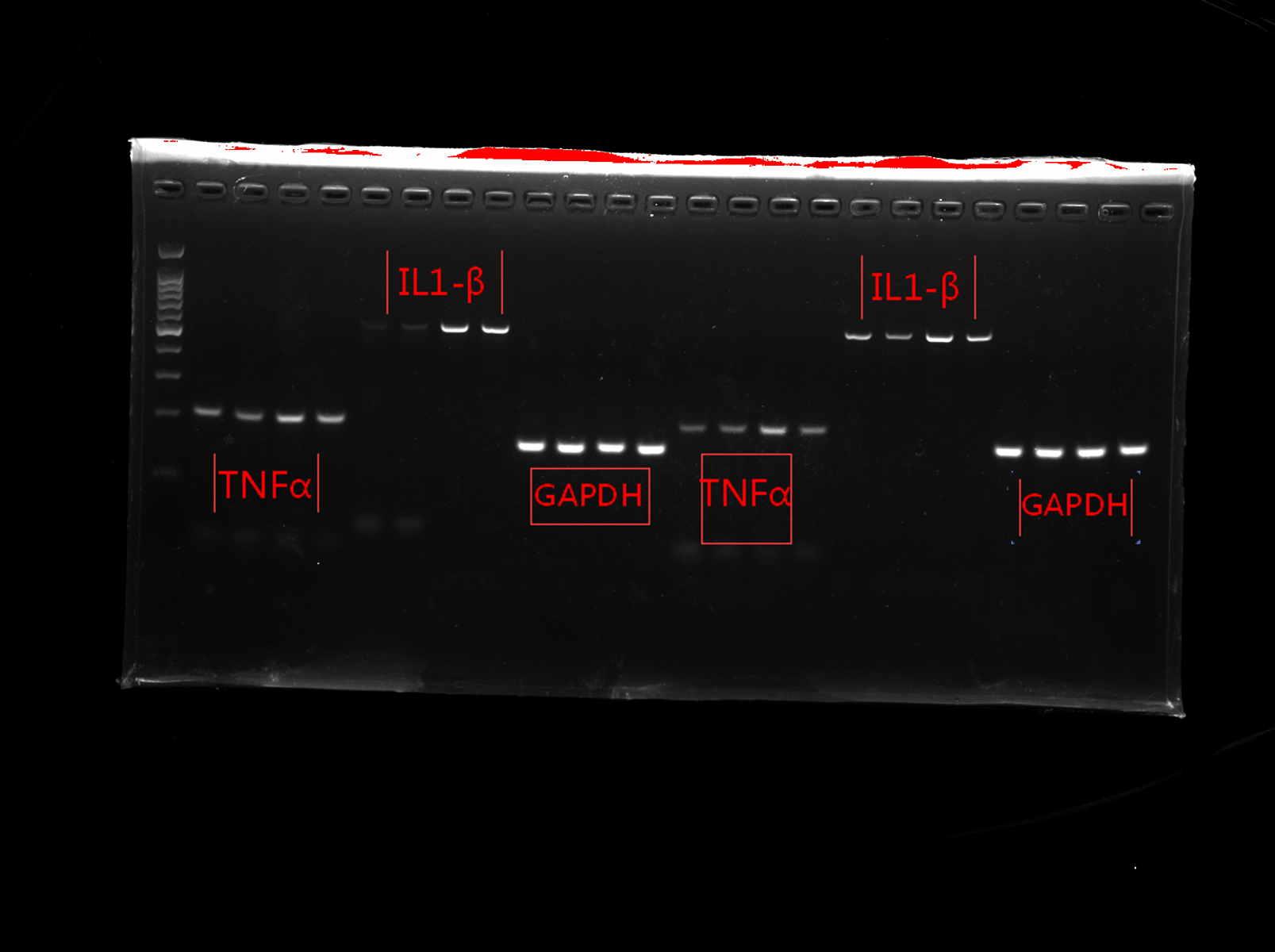

Supplement: Supplemental Information 1 [file peerj-08-10262-s001.zip › raw data1/FIG5/TNFa┴ IL1a┬ GAPDH .png]

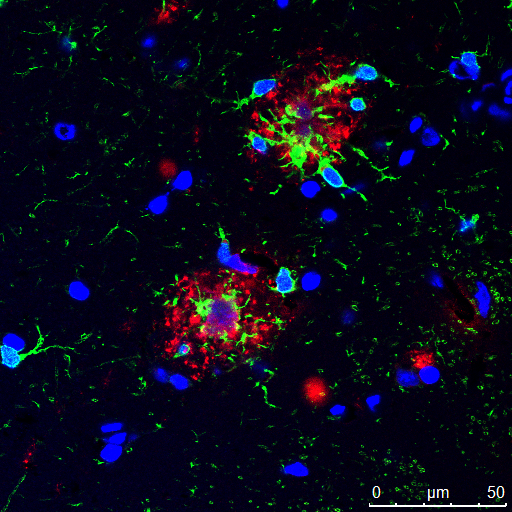

Supplement: Supplemental Information 2 [file peerj-08-10262-s002.zip › raw data2-1/FIG4/AD+Bi Aa┬ Iba-1 confocal/AD/Experiment_2TAO AD 63X -3_z0.png]

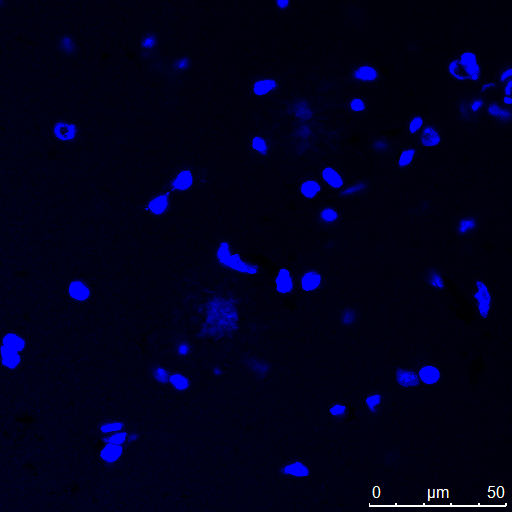

Supplement: Supplemental Information 2 [file peerj-08-10262-s002.zip › raw data2-1/FIG4/AD+Bi Aa┬ Iba-1 confocal/AD/Experiment_2TAO AD 63X -3_z0_ch00.png]

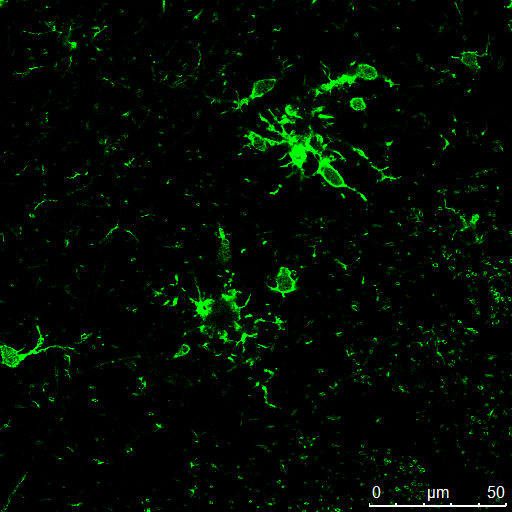

Supplement: Supplemental Information 2 [file peerj-08-10262-s002.zip › raw data2-1/FIG4/AD+Bi Aa┬ Iba-1 confocal/AD/Experiment_2TAO AD 63X -3_z0_ch01.png]

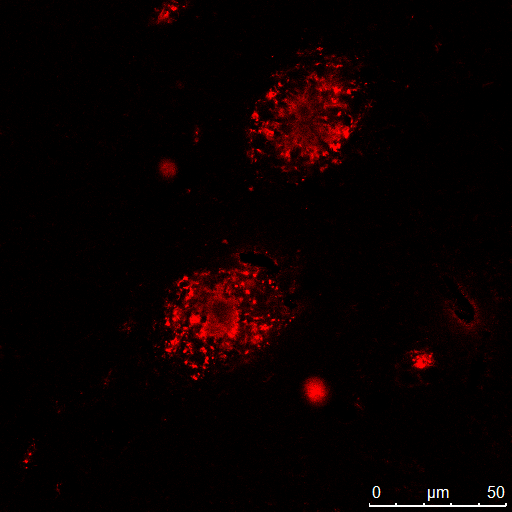

Supplement: Supplemental Information 2 [file peerj-08-10262-s002.zip › raw data2-1/FIG4/AD+Bi Aa┬ Iba-1 confocal/AD/Experiment_2TAO AD 63X -3_z0_ch02.png]

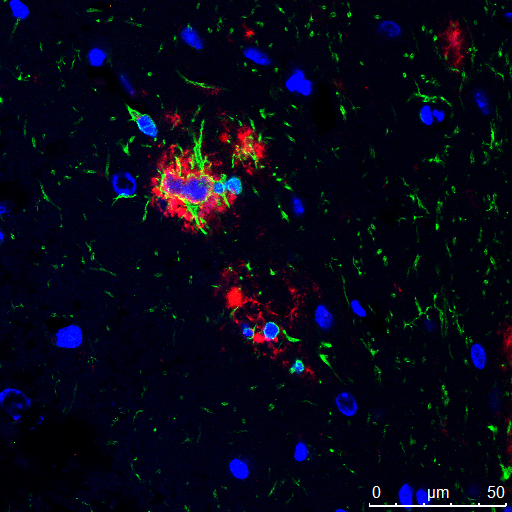

Supplement: Supplemental Information 2 [file peerj-08-10262-s002.zip › raw data2-1/FIG4/AD+Bi Aa┬ Iba-1 confocal/ADú1⁄2BI/Experiment_3TAO AD+Bi 63X -2_z0.png]

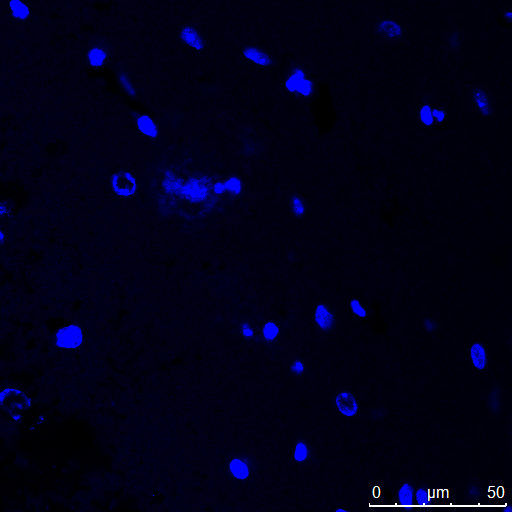

Supplement: Supplemental Information 2 [file peerj-08-10262-s002.zip › raw data2-1/FIG4/AD+Bi Aa┬ Iba-1 confocal/ADú1⁄2BI/Experiment_3TAO AD+Bi 63X -2_z0_ch00.png]

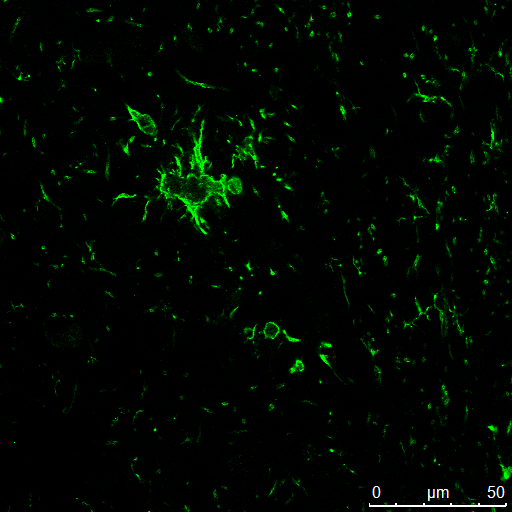

Supplement: Supplemental Information 2 [file peerj-08-10262-s002.zip › raw data2-1/FIG4/AD+Bi Aa┬ Iba-1 confocal/ADú1⁄2BI/Experiment_3TAO AD+Bi 63X -2_z0_ch01.png]

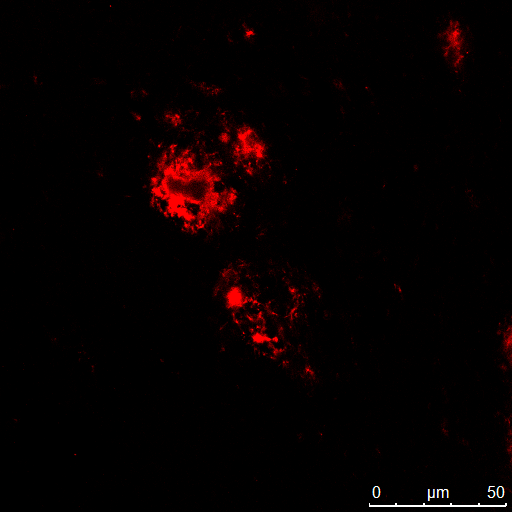

Supplement: Supplemental Information 2 [file peerj-08-10262-s002.zip › raw data2-1/FIG4/AD+Bi Aa┬ Iba-1 confocal/ADú1⁄2BI/Experiment_3TAO AD+Bi 63X -2_z0_ch02.png]

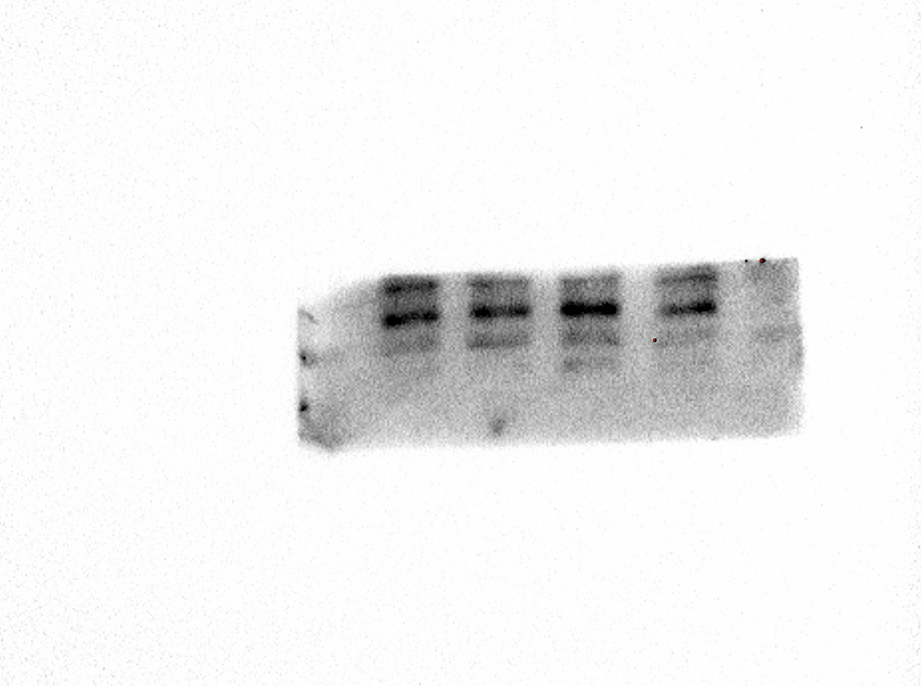

Supplement: Supplemental Information 2 [file peerj-08-10262-s002.zip › raw data2-1/FIG4/Iba1.png]

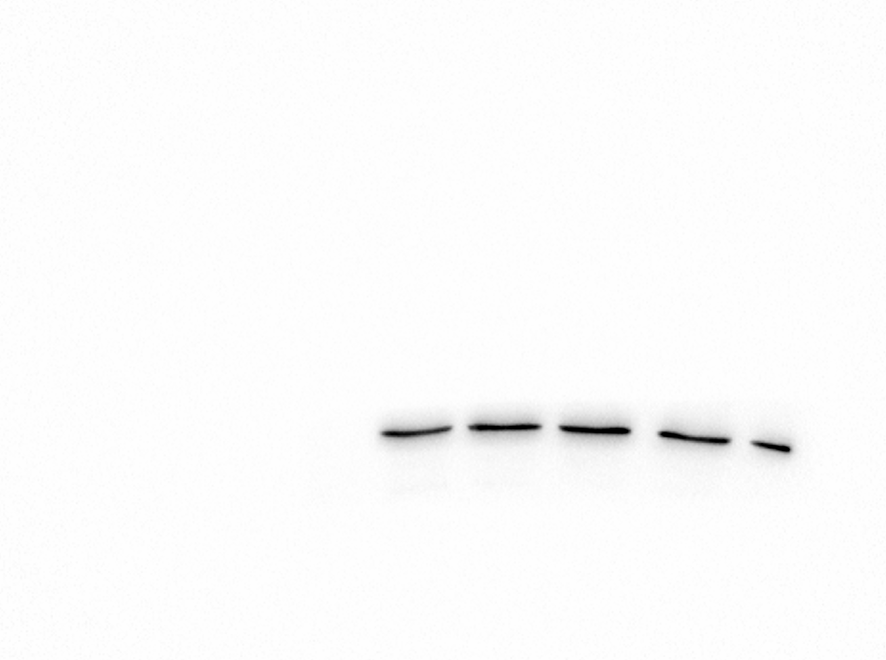

Supplement: Supplemental Information 2 [file peerj-08-10262-s002.zip › raw data2-1/FIG4/actin.png]

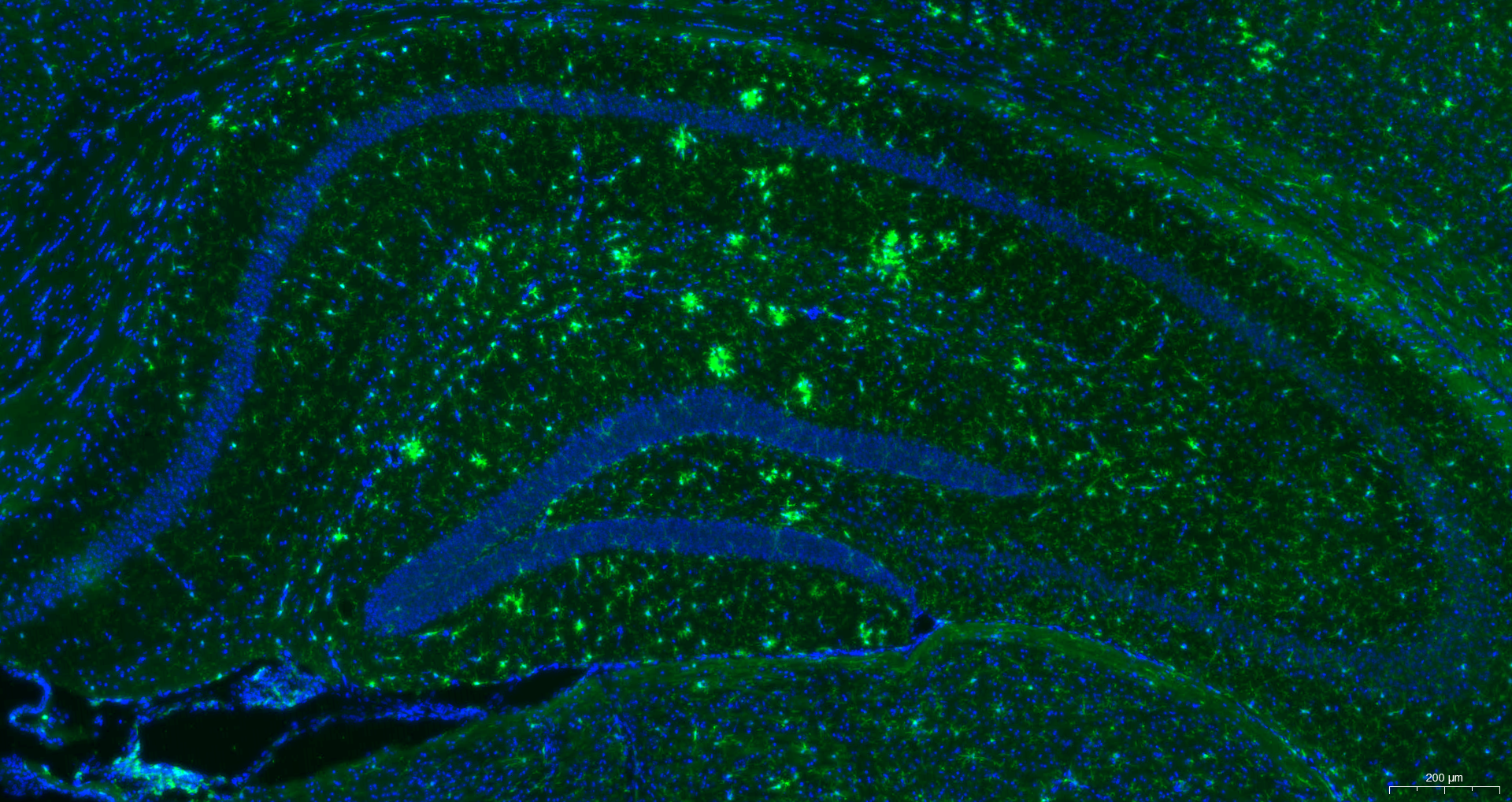

Supplement: Supplemental Information 2 [file peerj-08-10262-s002.zip › raw data2-1/FIG4/║ú┬φ/AD/4-L13_7.0x.png]

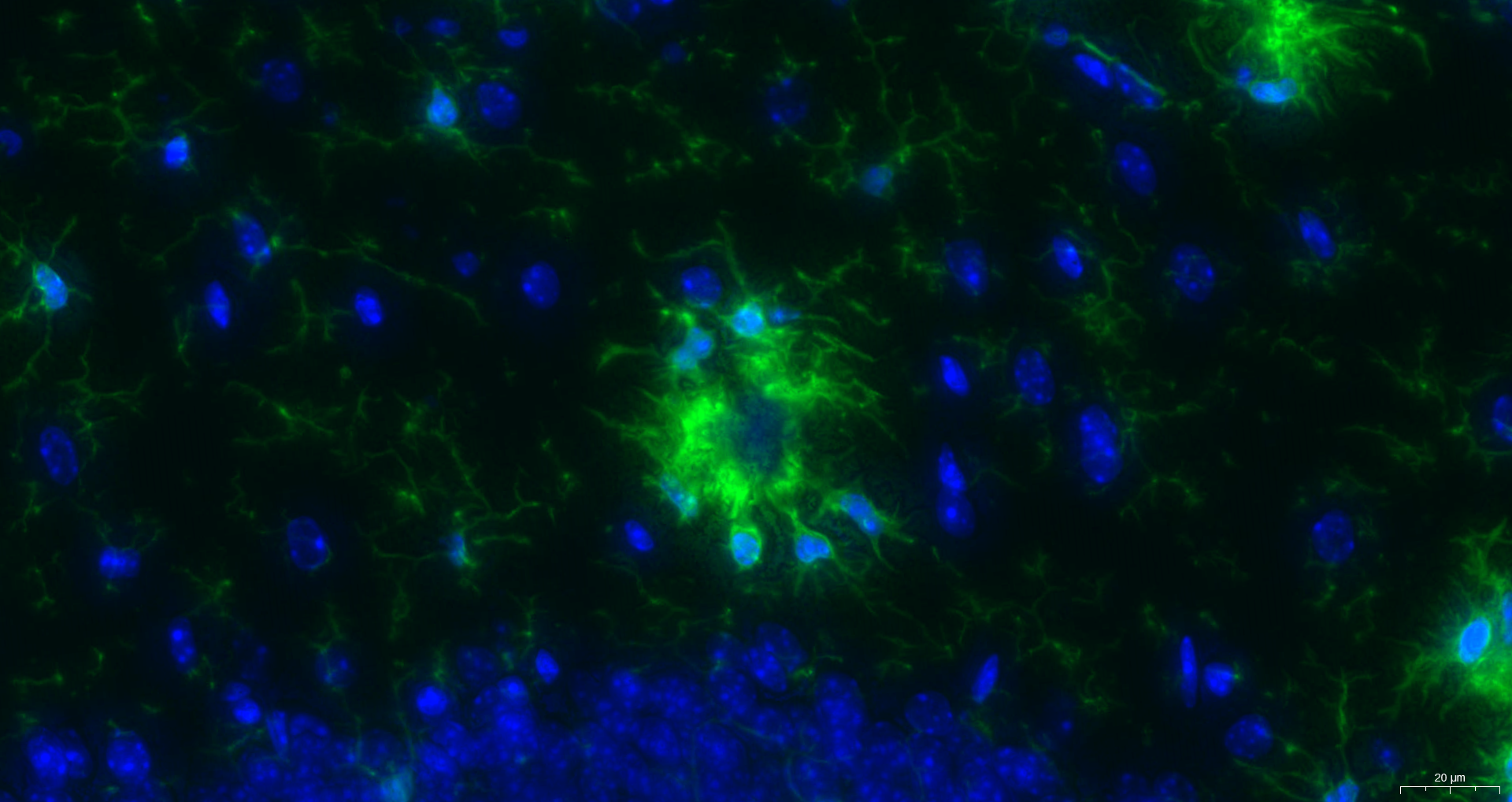

Supplement: Supplemental Information 2 [file peerj-08-10262-s002.zip › raw data2-1/FIG4/║ú┬φ/AD/4-L1_63.0x.png]

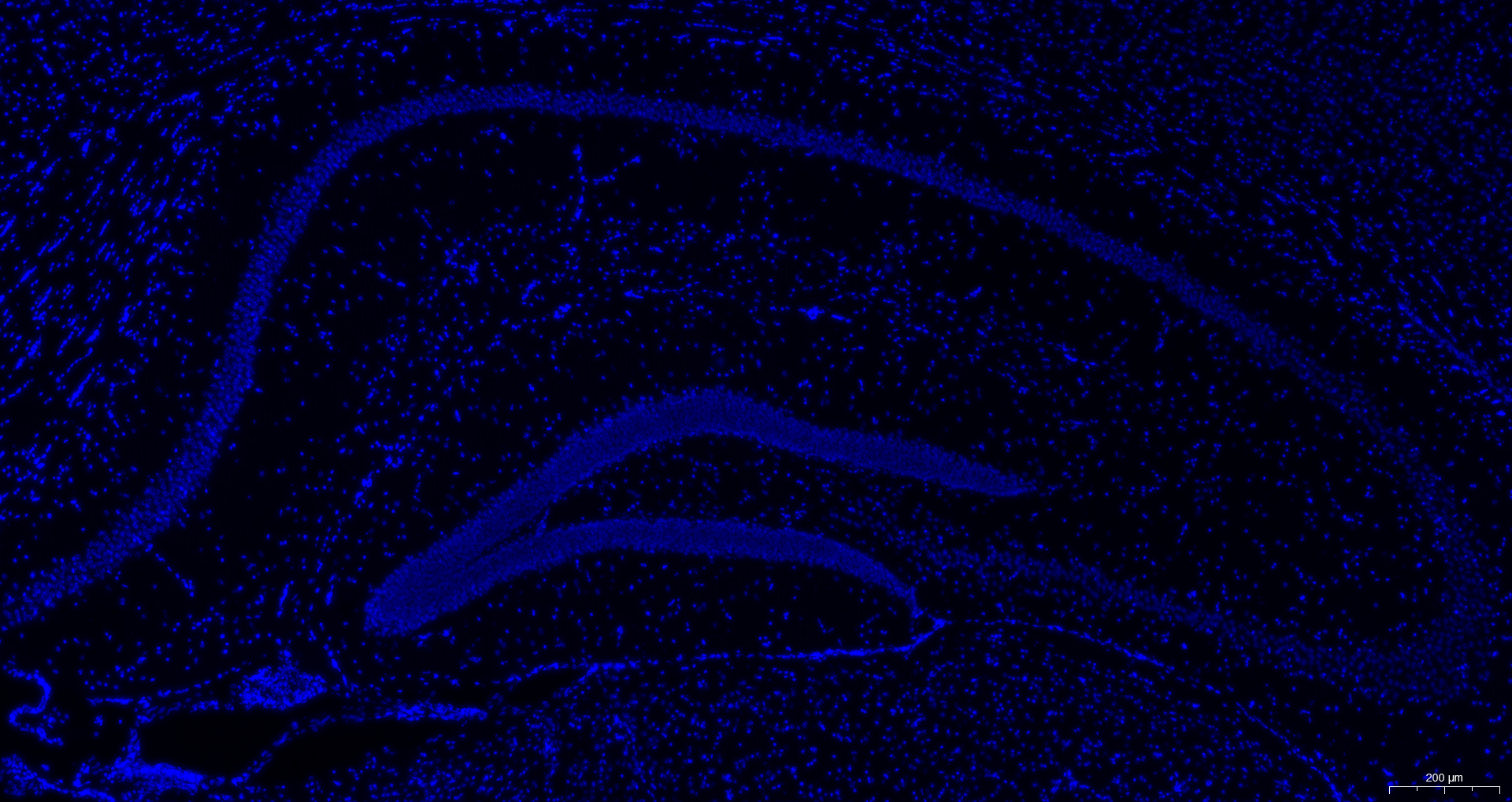

Supplement: Supplemental Information 2 [file peerj-08-10262-s002.zip › raw data2-1/FIG4/║ú┬φ/AD/4-L1_7.0x.png]

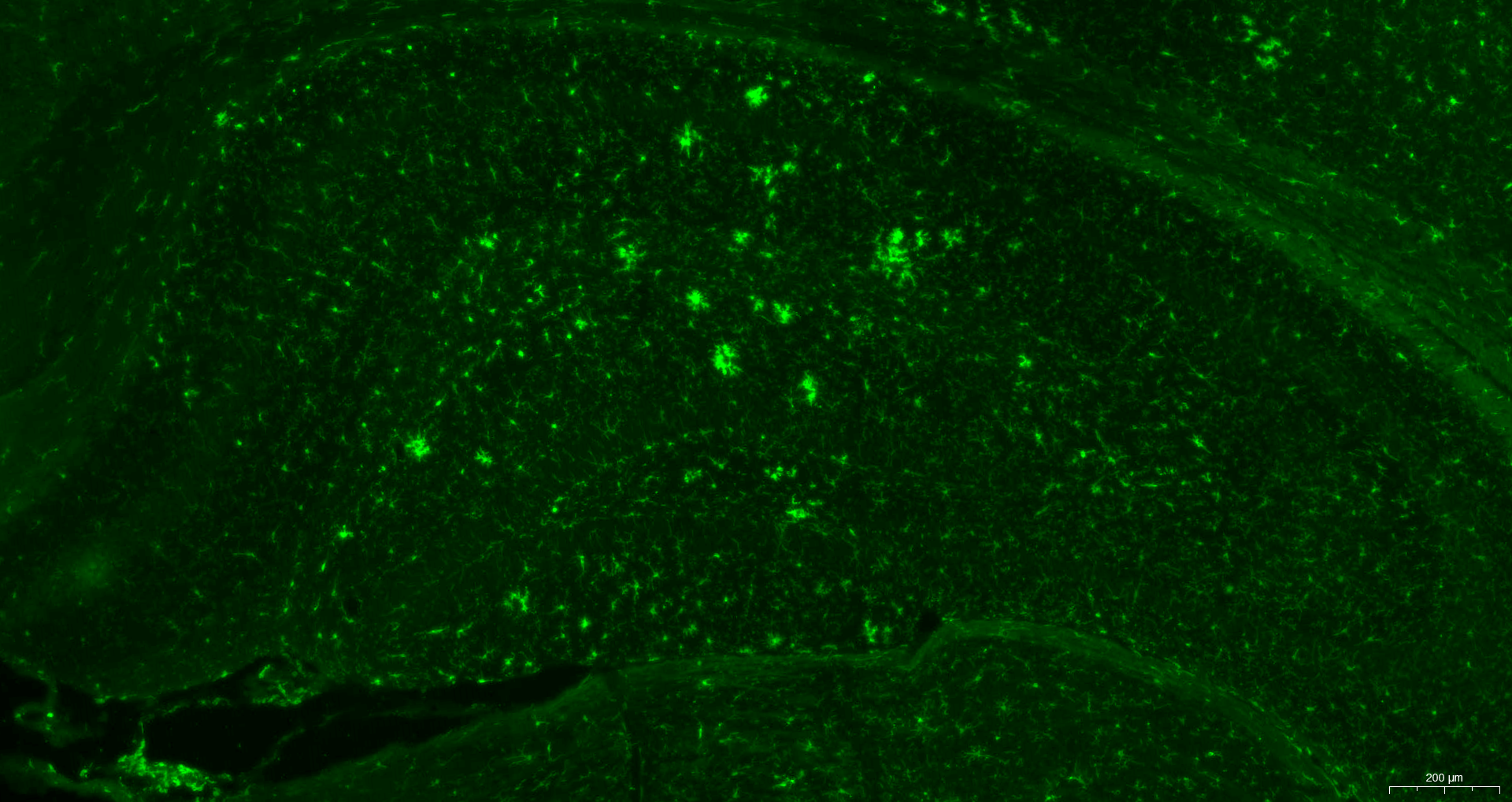

Supplement: Supplemental Information 2 [file peerj-08-10262-s002.zip › raw data2-1/FIG4/║ú┬φ/AD/4-L2_7.0x.png]

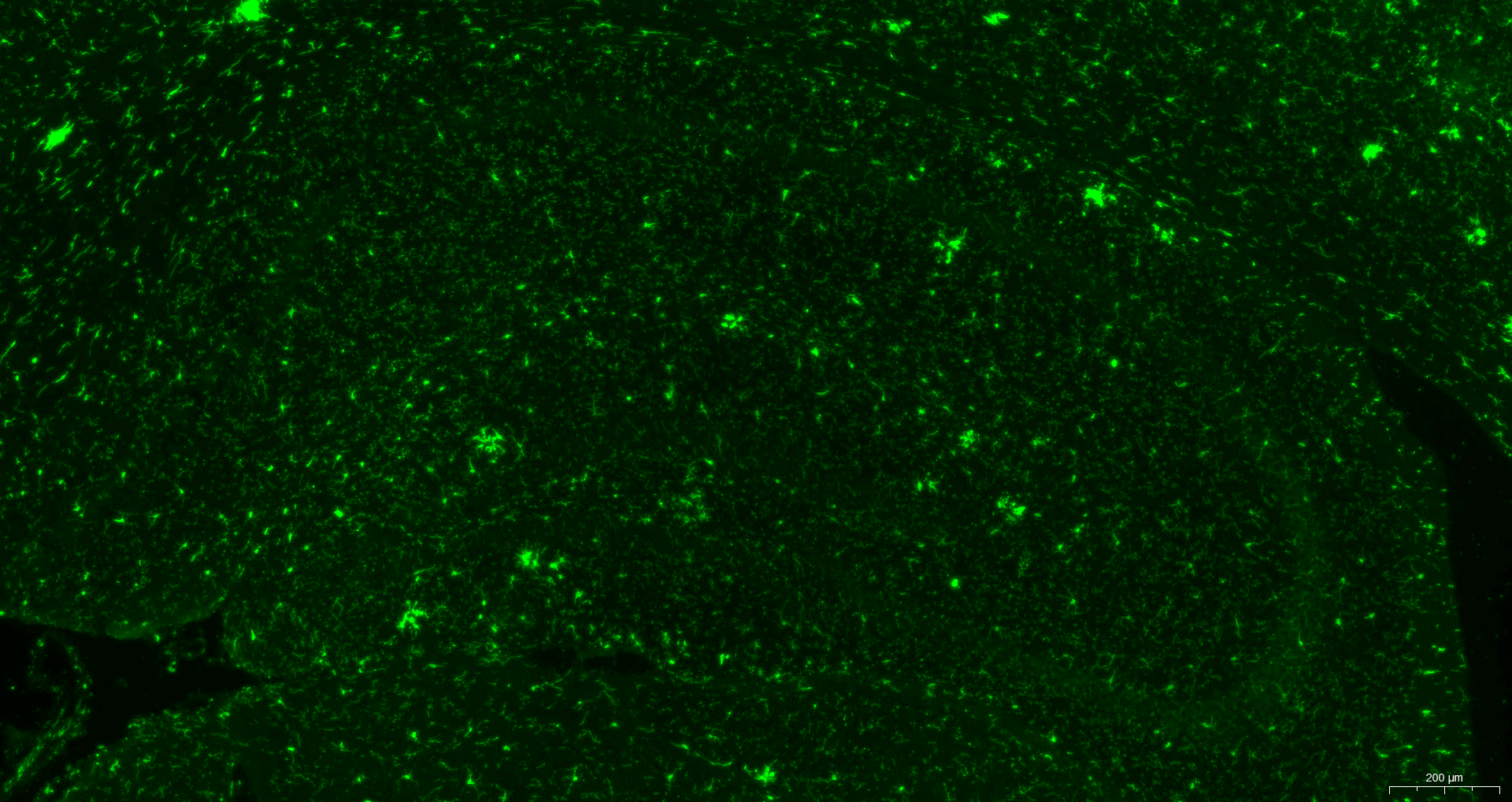

Supplement: Supplemental Information 2 [file peerj-08-10262-s002.zip › raw data2-1/FIG4/║ú┬φ/AD+BI/4-M12_7.0x.png]

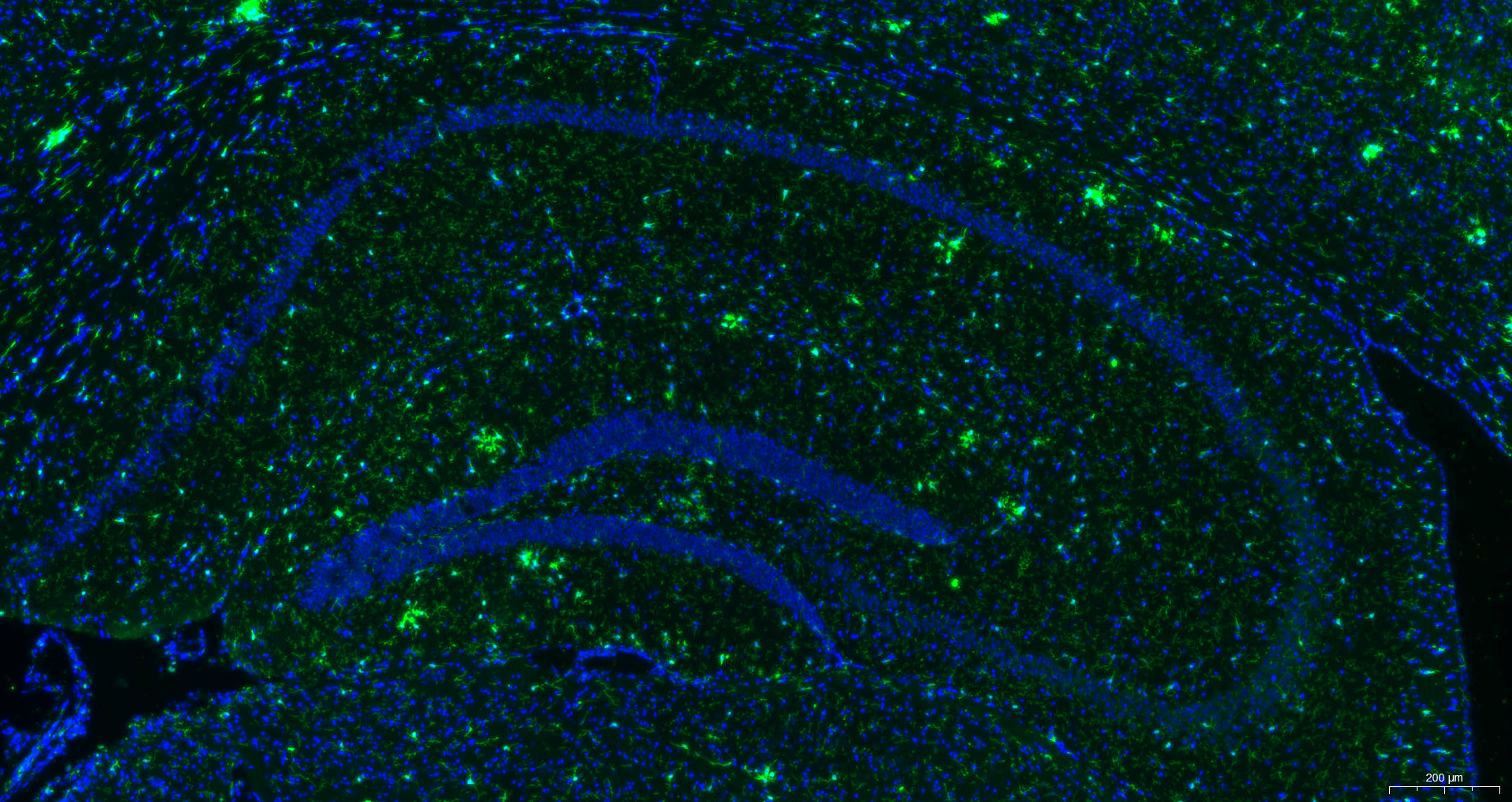

Supplement: Supplemental Information 2 [file peerj-08-10262-s002.zip › raw data2-1/FIG4/║ú┬φ/AD+BI/4-M13_7.0x.png]

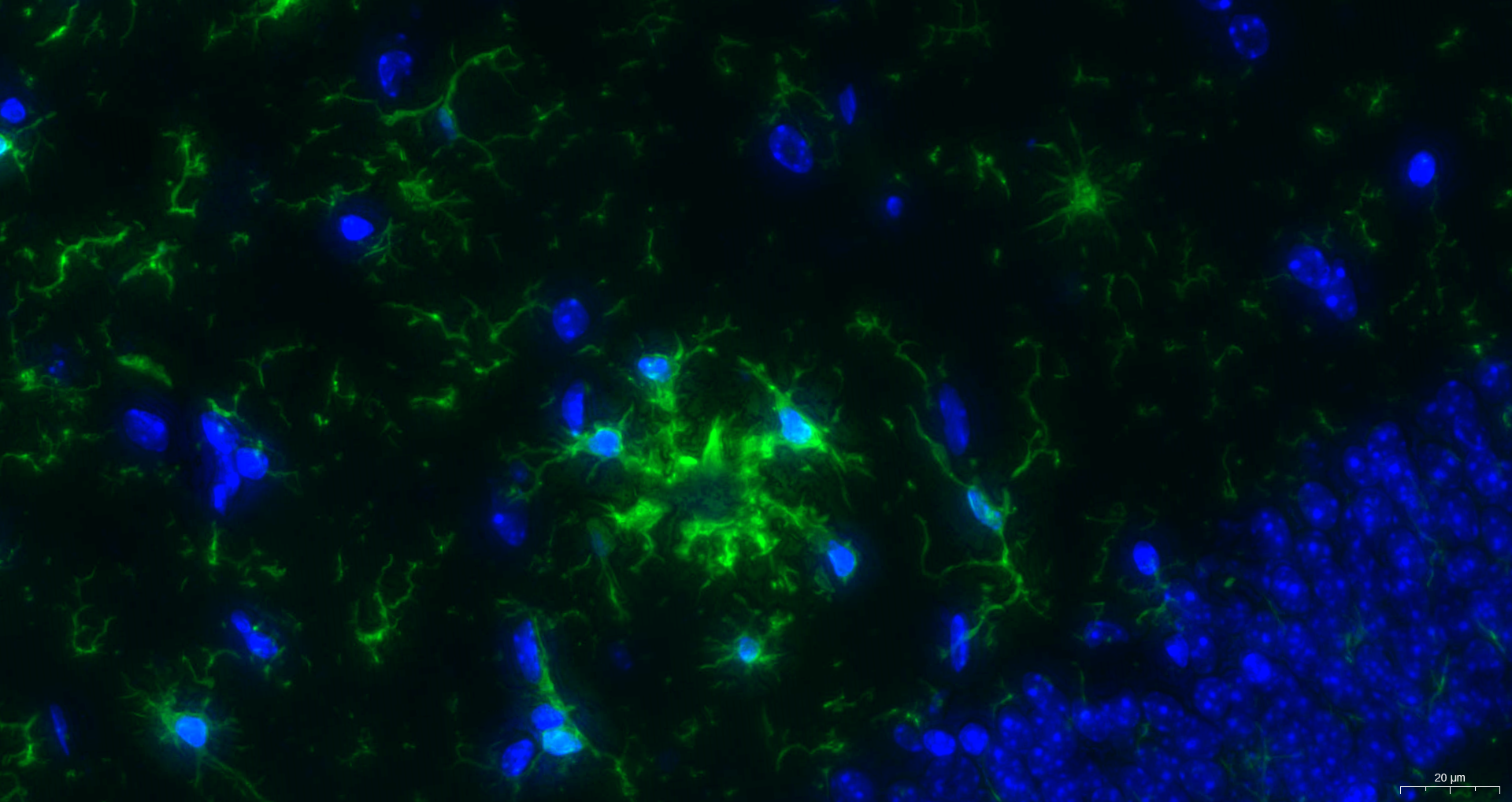

Supplement: Supplemental Information 2 [file peerj-08-10262-s002.zip › raw data2-1/FIG4/║ú┬φ/AD+BI/4-M1_63.0x.png]

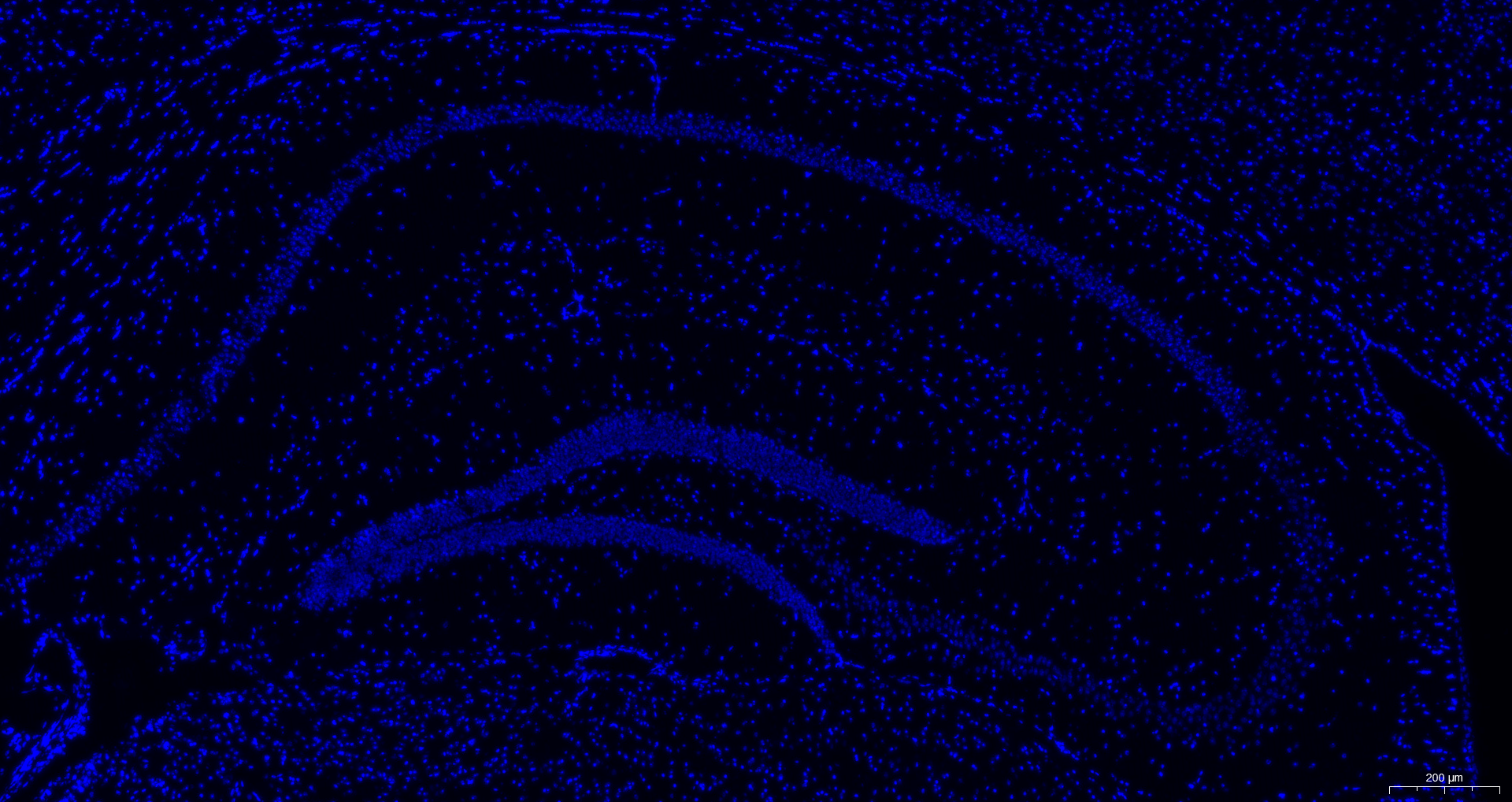

Supplement: Supplemental Information 2 [file peerj-08-10262-s002.zip › raw data2-1/FIG4/║ú┬φ/AD+BI/4-M1_7.0x.png]

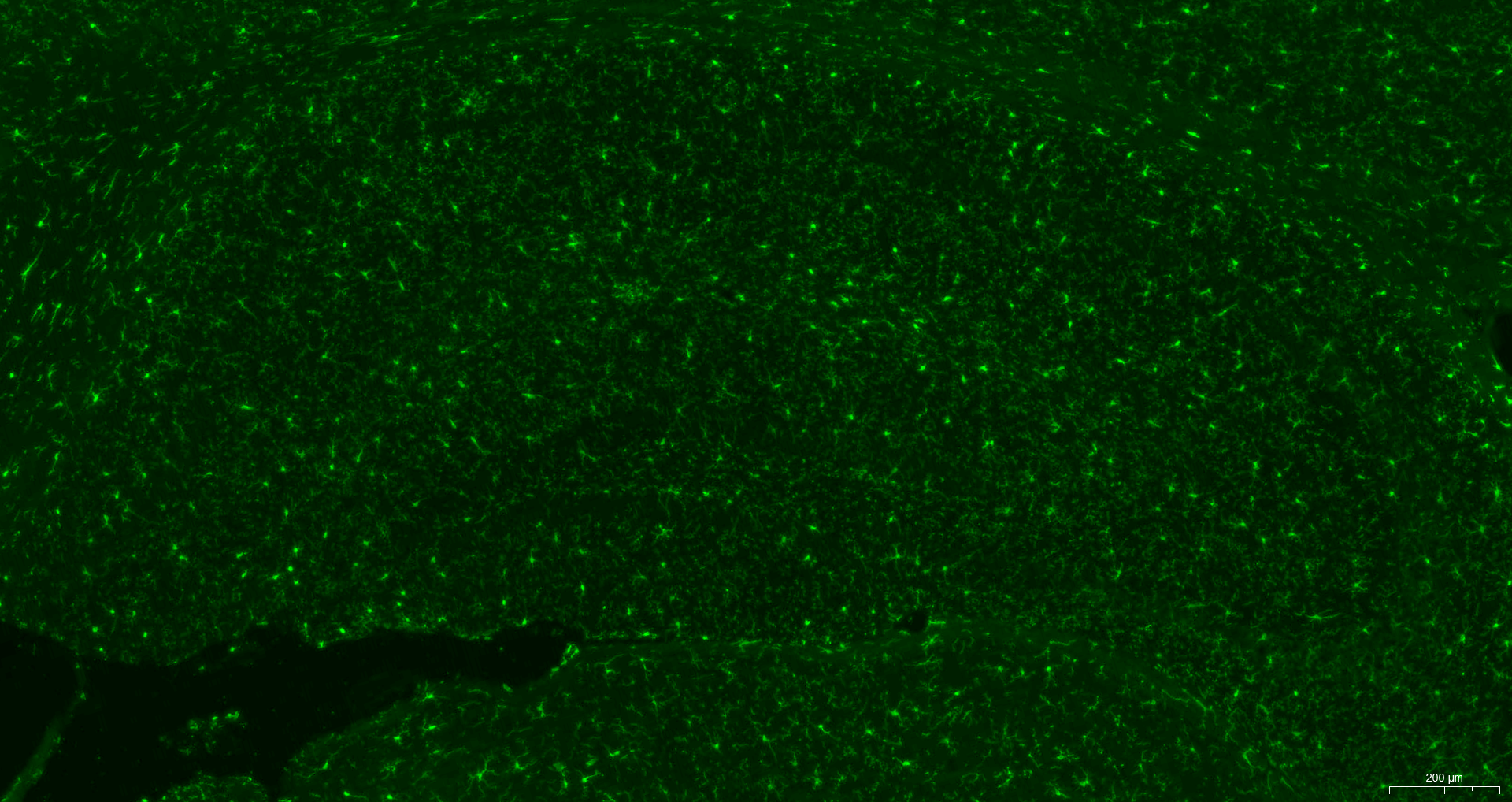

Supplement: Supplemental Information 2 [file peerj-08-10262-s002.zip › raw data2-1/FIG4/║ú┬φ/WT/4-J12_7.0x.png]

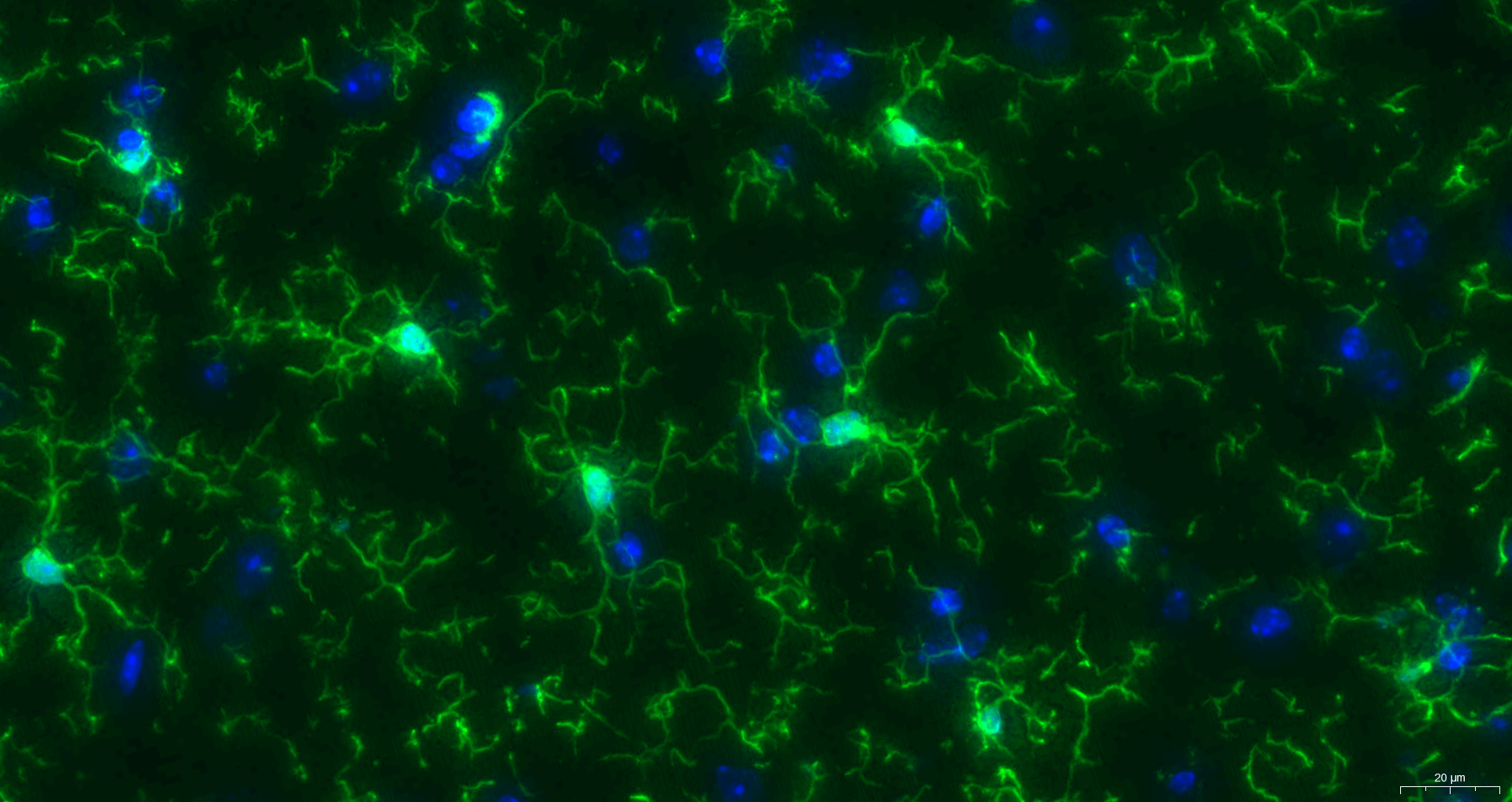

Supplement: Supplemental Information 2 [file peerj-08-10262-s002.zip › raw data2-1/FIG4/║ú┬φ/WT/4-J1_63.0x.png]

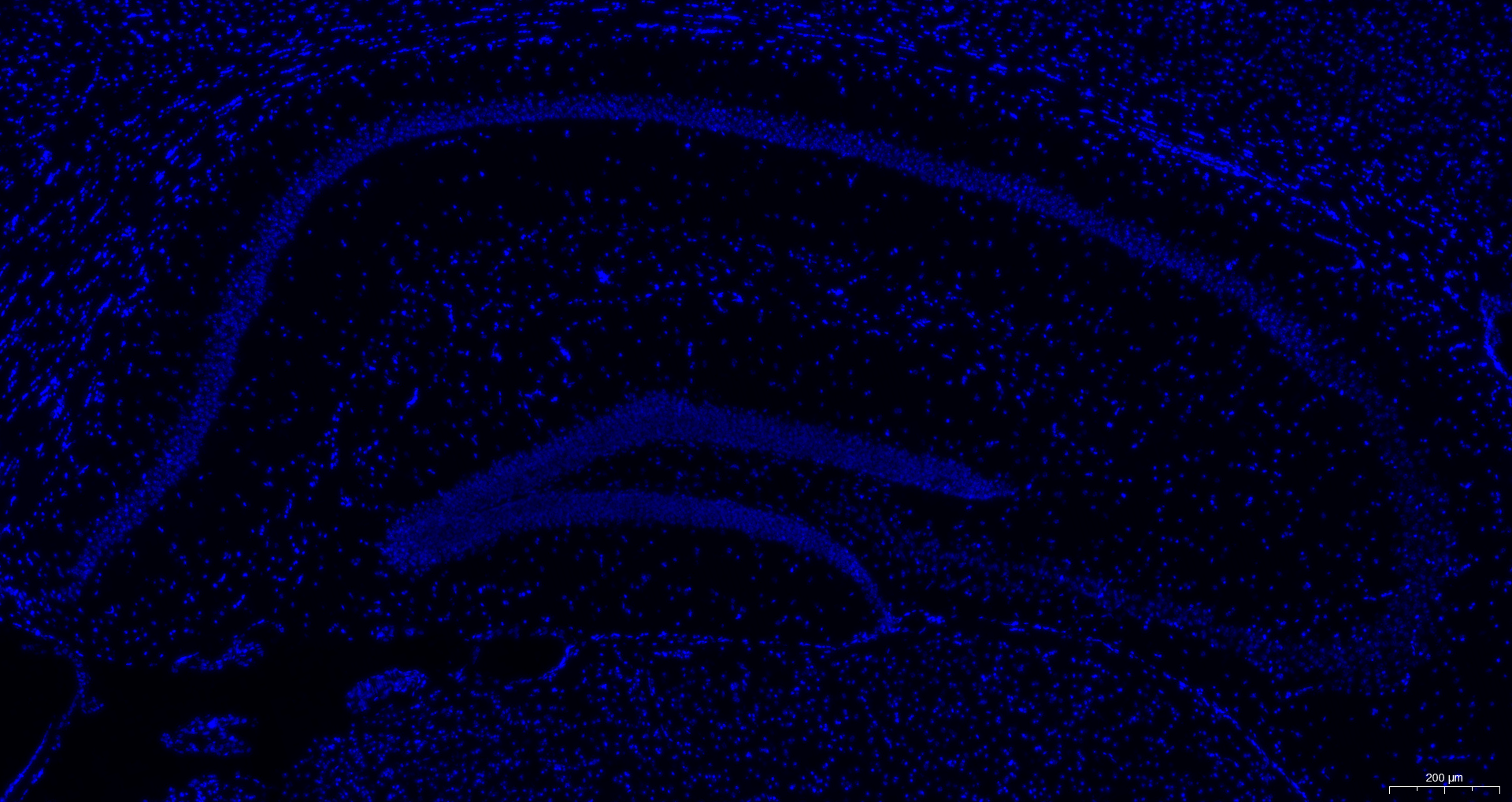

Supplement: Supplemental Information 2 [file peerj-08-10262-s002.zip › raw data2-1/FIG4/║ú┬φ/WT/4-J1_7.0x.png]

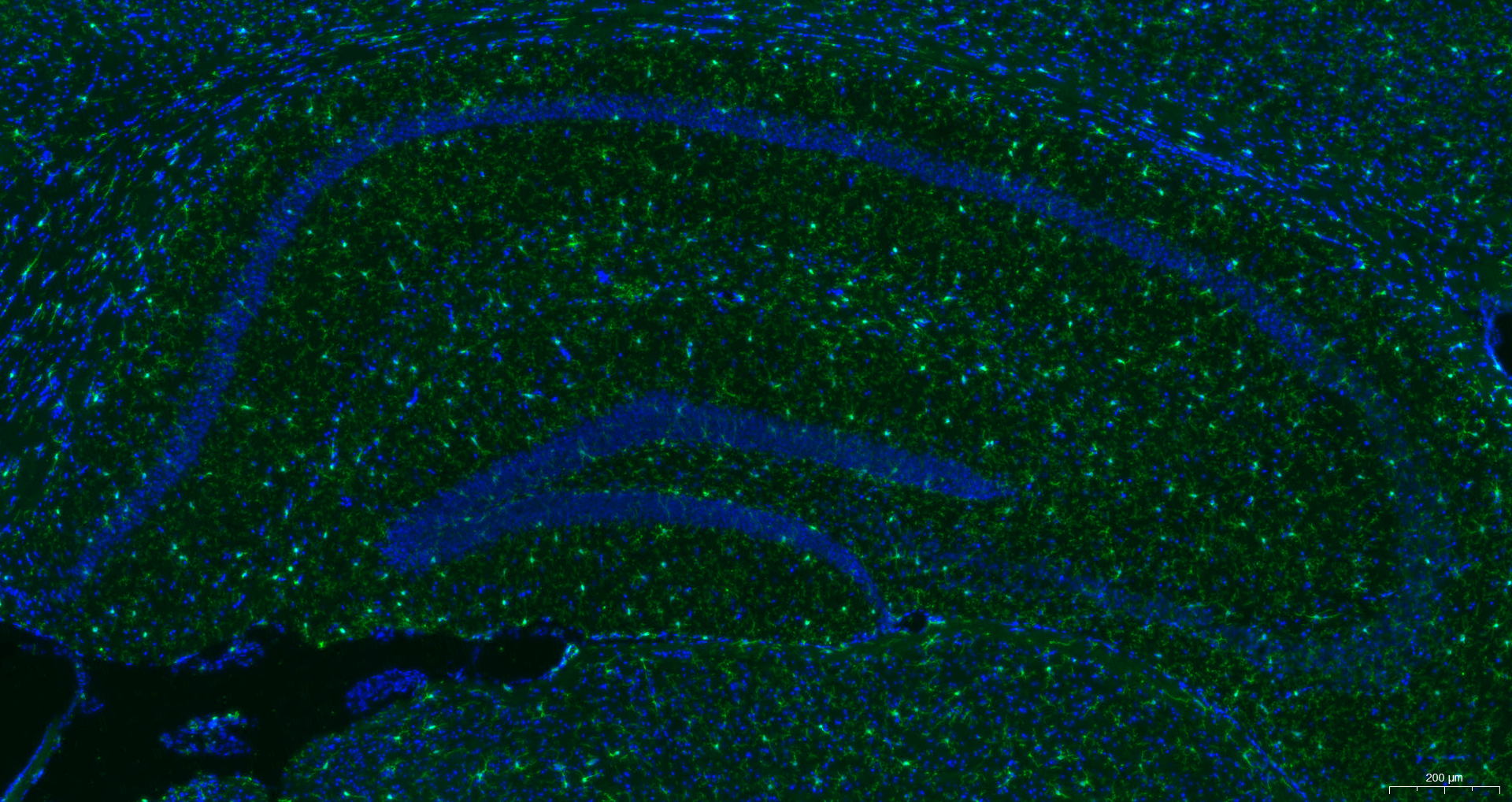

Supplement: Supplemental Information 2 [file peerj-08-10262-s002.zip › raw data2-1/FIG4/║ú┬φ/WT/4-J3_7.0x.png]

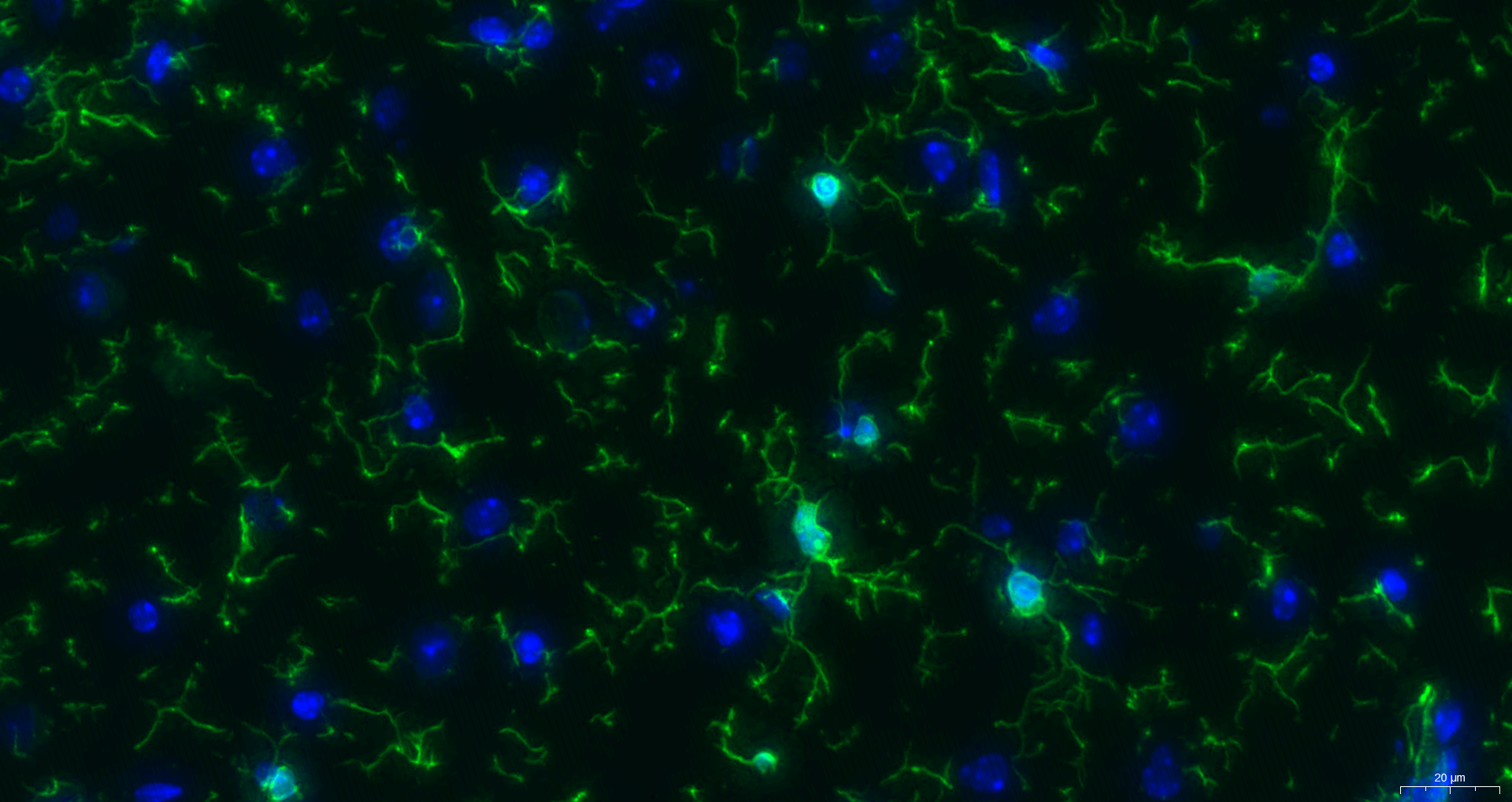

Supplement: Supplemental Information 2 [file peerj-08-10262-s002.zip › raw data2-1/FIG4/║ú┬φ/WT +BI/4-K1_63.0x.png]

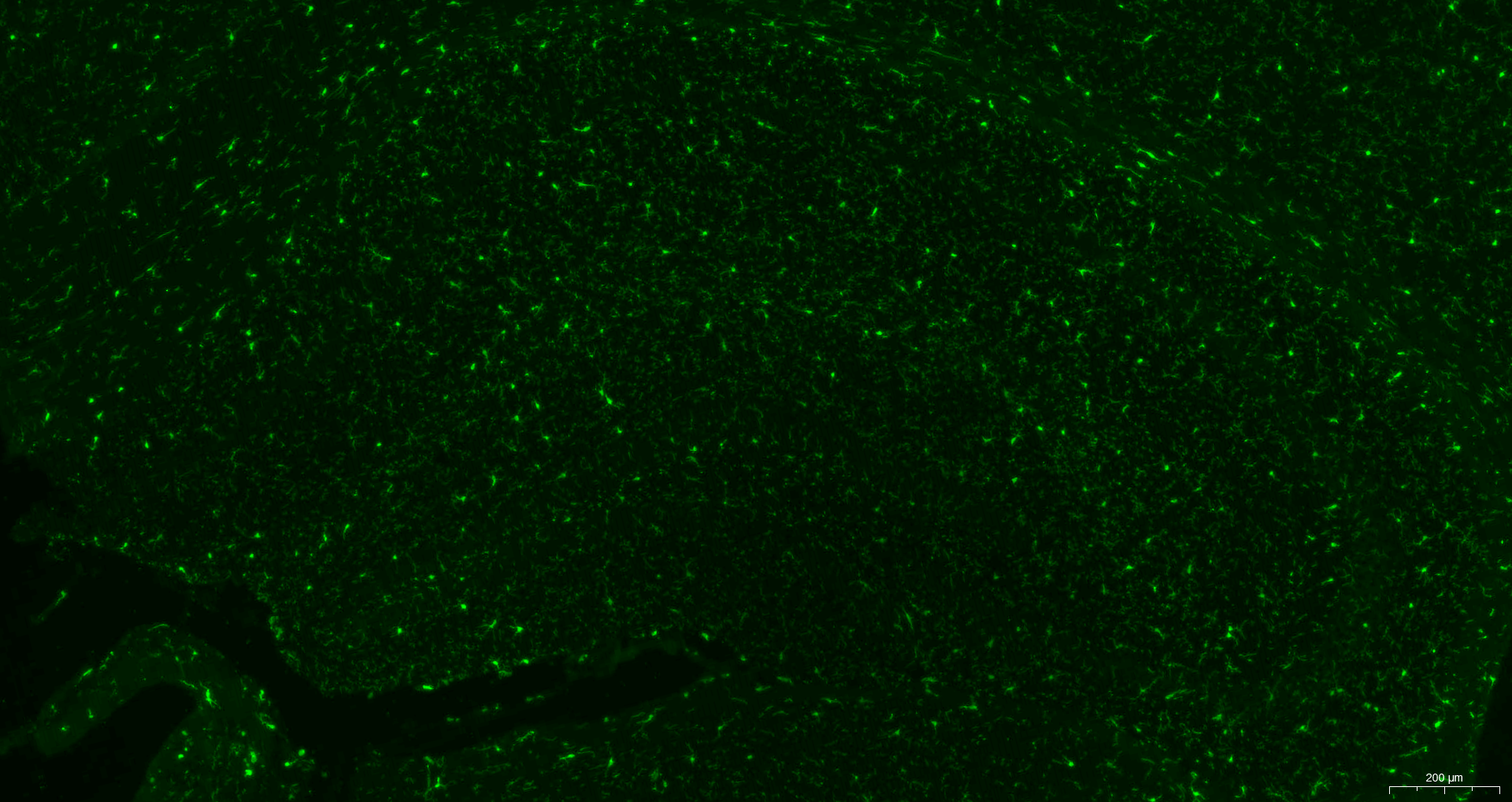

Supplement: Supplemental Information 2 [file peerj-08-10262-s002.zip › raw data2-1/FIG4/║ú┬φ/WT +BI/4-K22_7.0x.png]

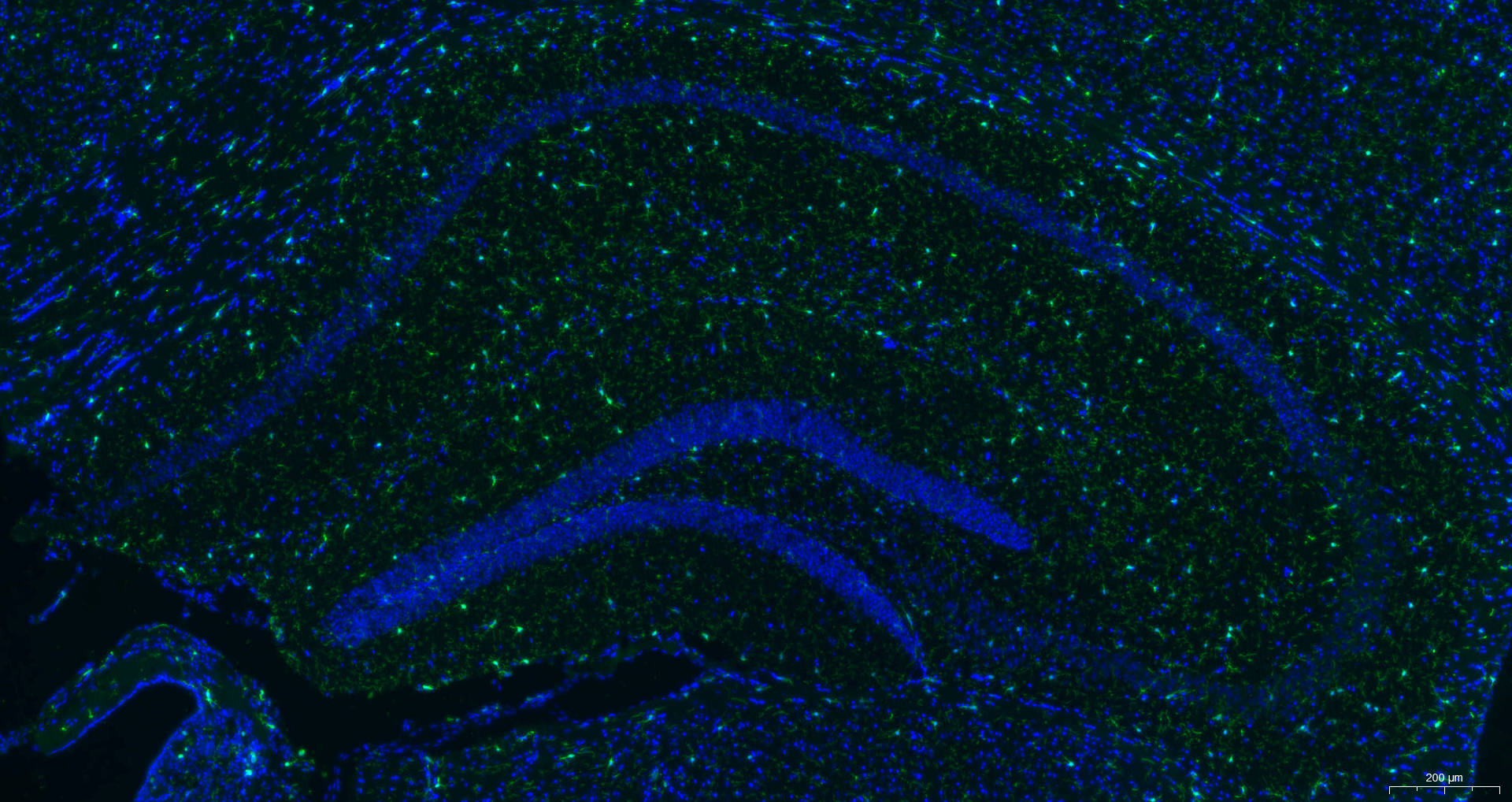

Supplement: Supplemental Information 2 [file peerj-08-10262-s002.zip › raw data2-1/FIG4/║ú┬φ/WT +BI/4-K23_7.0x.png]

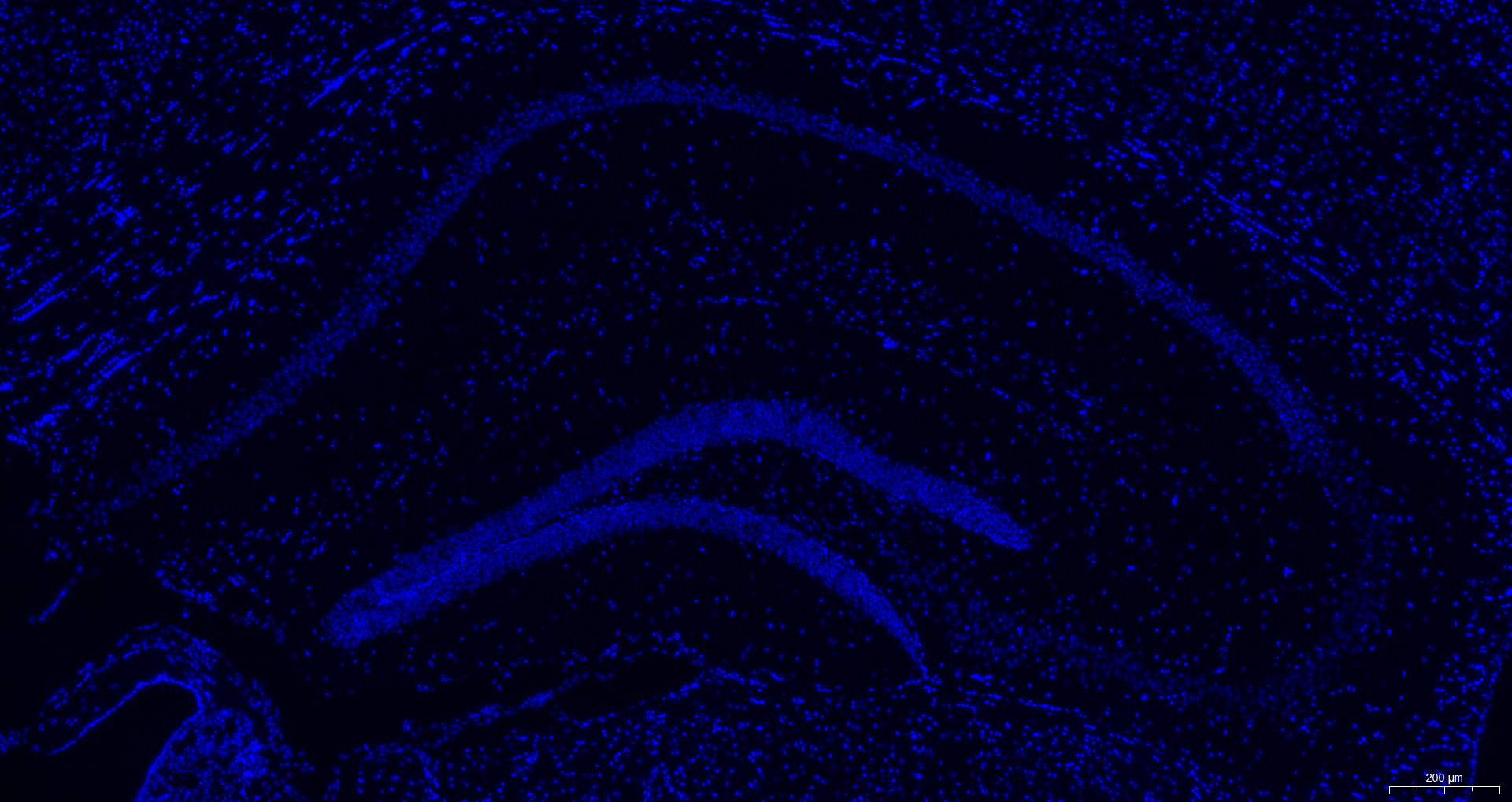

Supplement: Supplemental Information 2 [file peerj-08-10262-s002.zip › raw data2-1/FIG4/║ú┬φ/WT +BI/4-K2_7.0x.png]

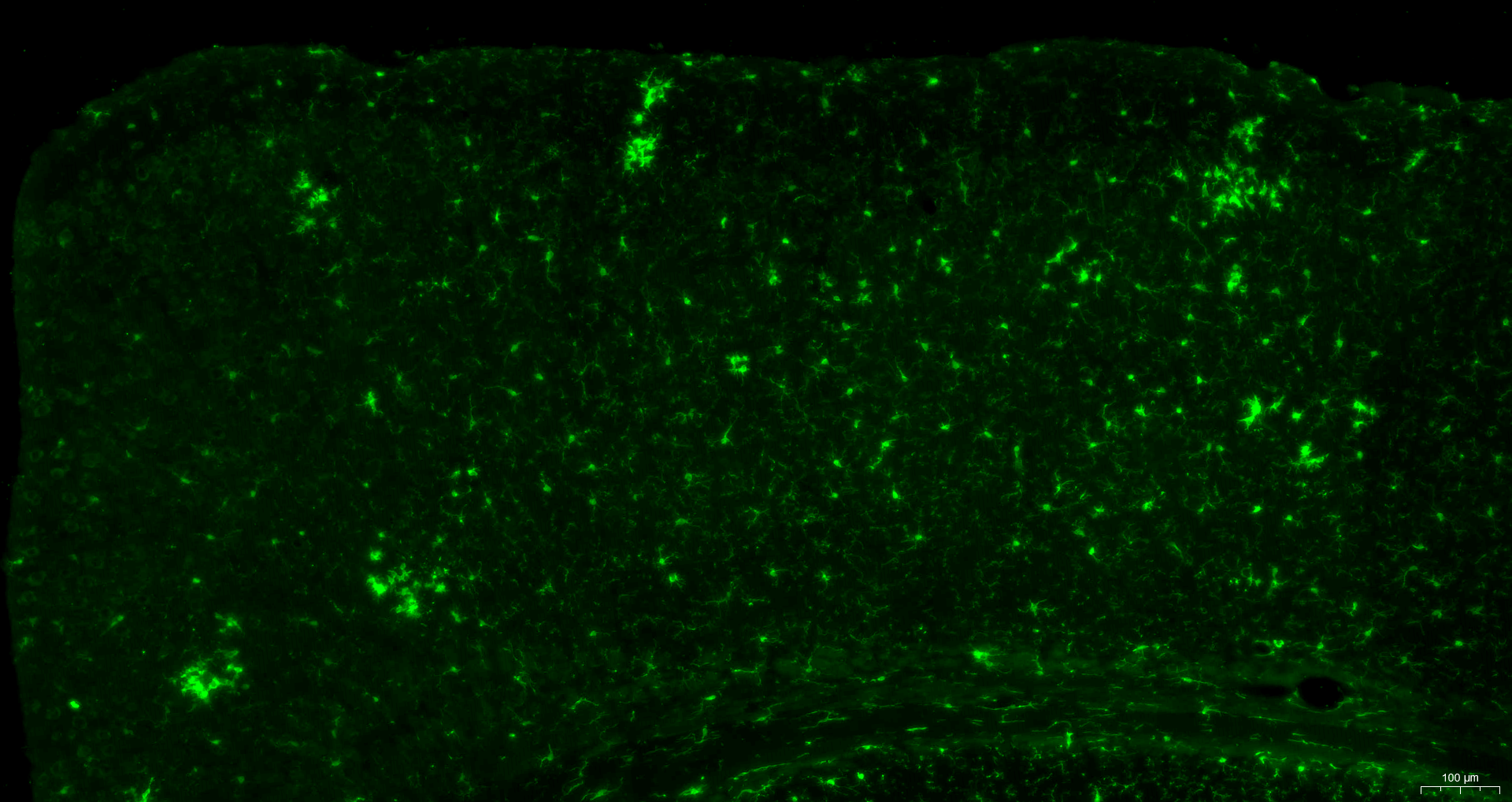

Supplement: Supplemental Information 3 [file peerj-08-10262-s003.zip › raw data2-2/AD/4-L11_10.0x.png]

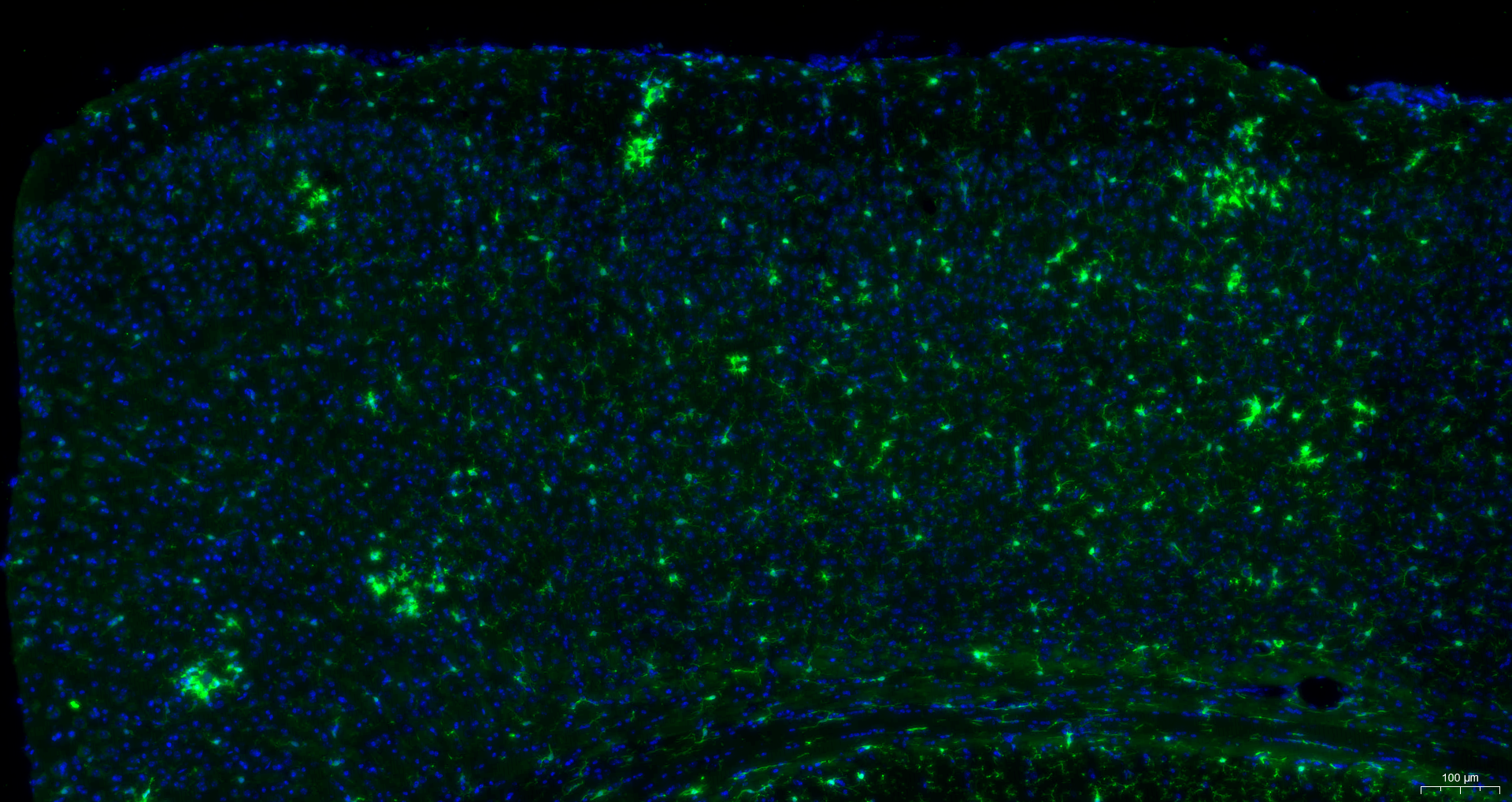

Supplement: Supplemental Information 3 [file peerj-08-10262-s003.zip › raw data2-2/AD/4-L12_10.0x.png]

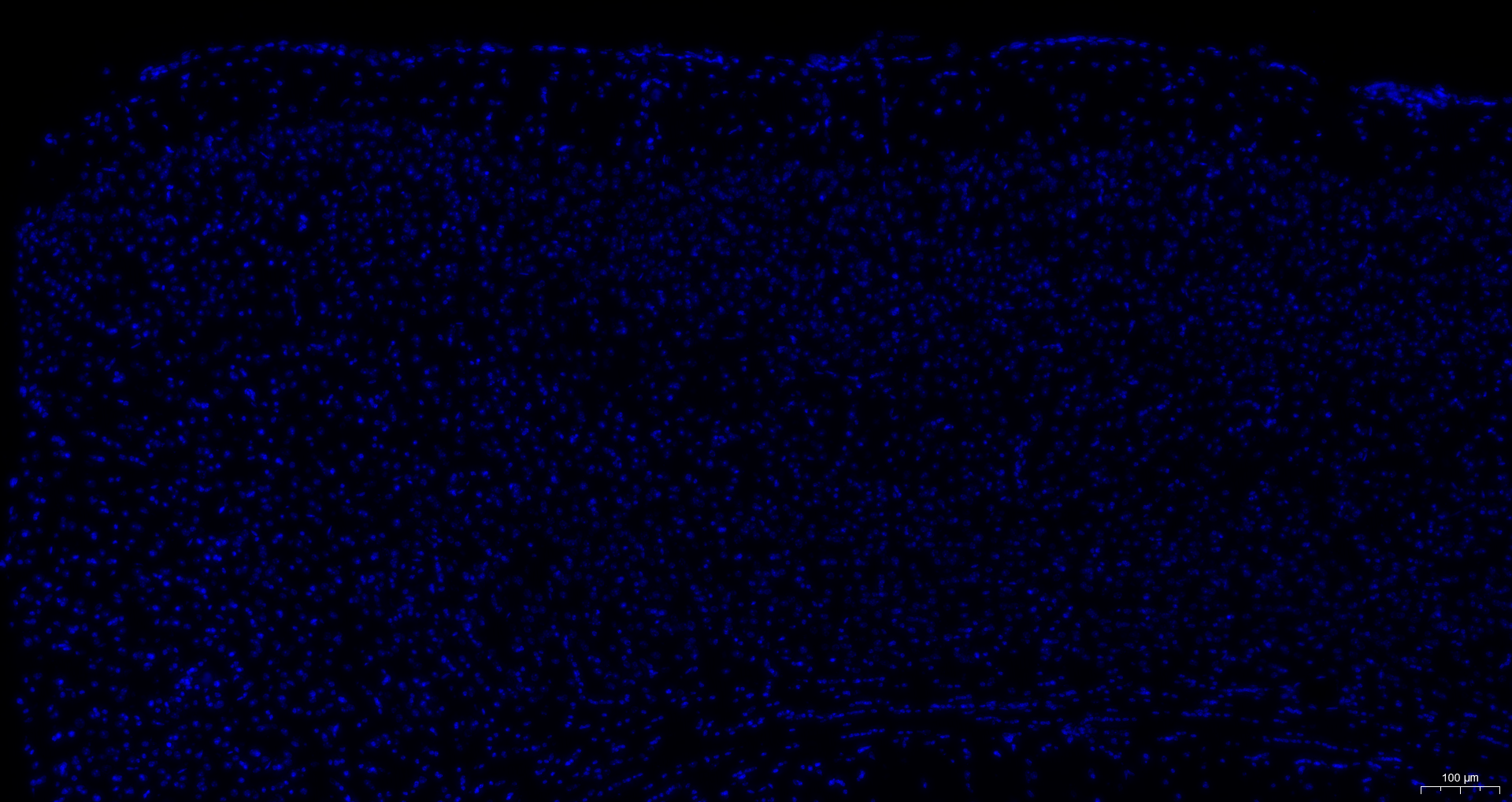

Supplement: Supplemental Information 3 [file peerj-08-10262-s003.zip › raw data2-2/AD/4-L1_10.0x.png]

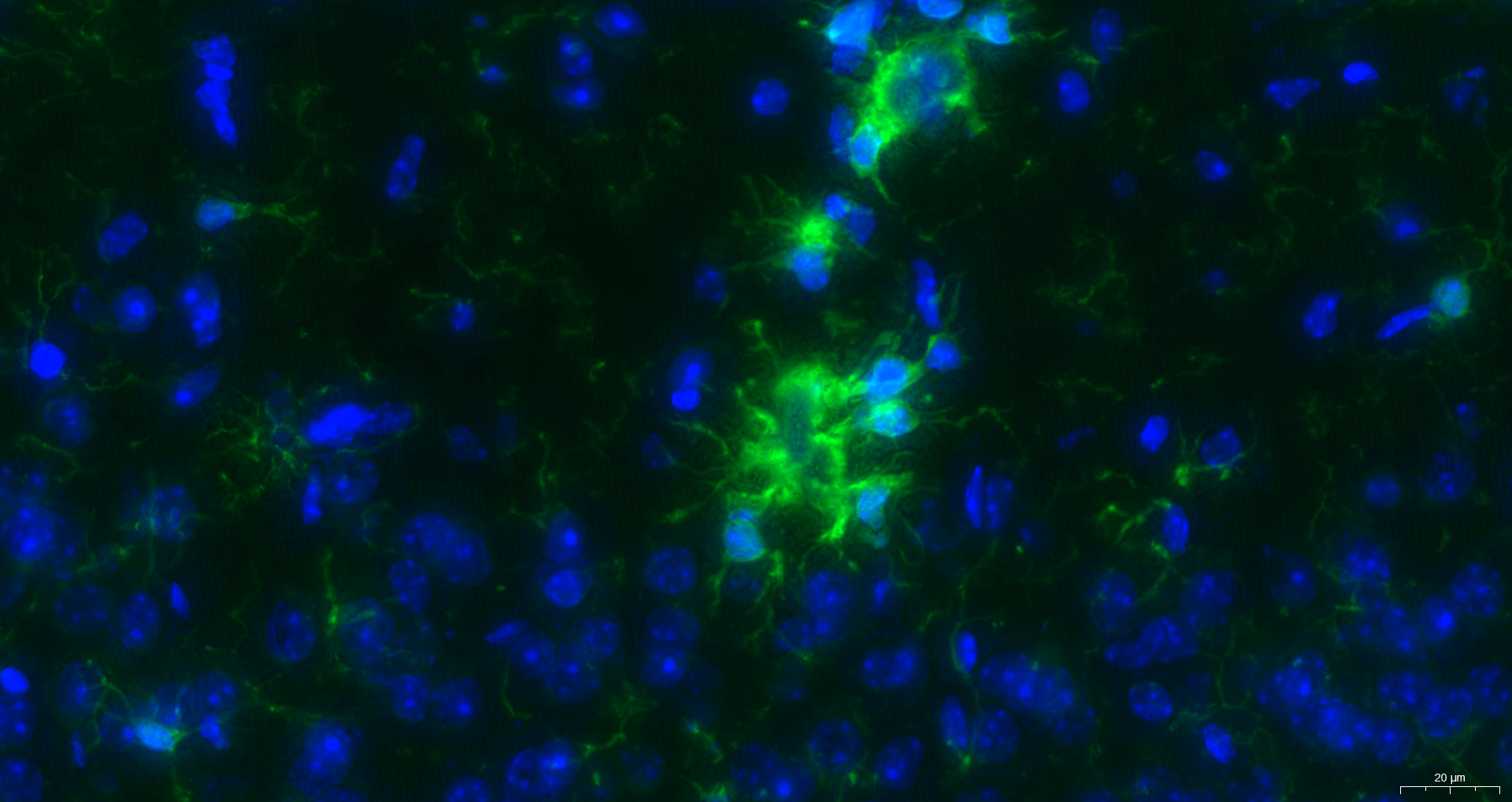

Supplement: Supplemental Information 3 [file peerj-08-10262-s003.zip › raw data2-2/AD/4-L1_63.0x.png]

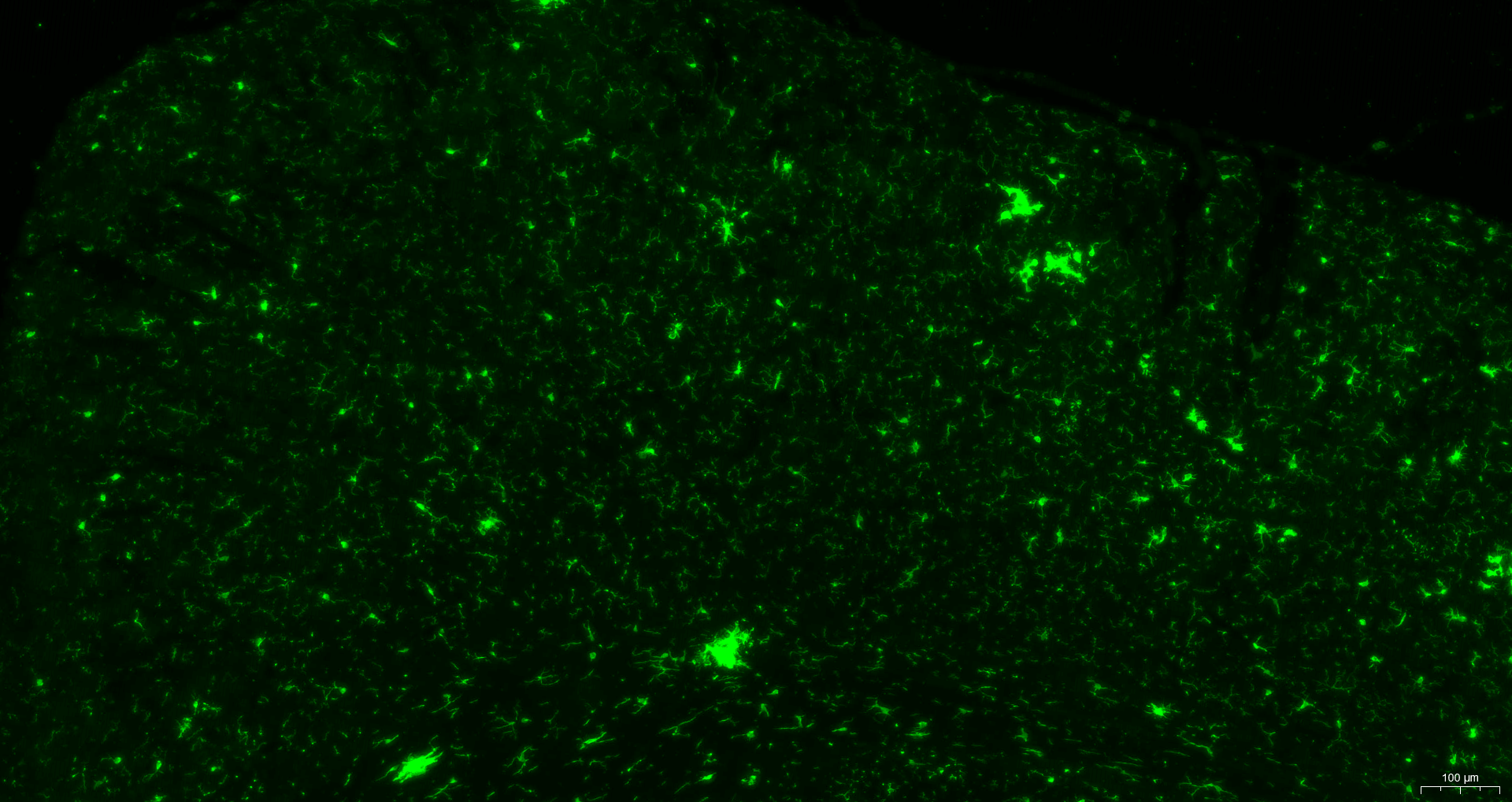

Supplement: Supplemental Information 3 [file peerj-08-10262-s003.zip › raw data2-2/AD+BI/4-M12_10.0x.png]

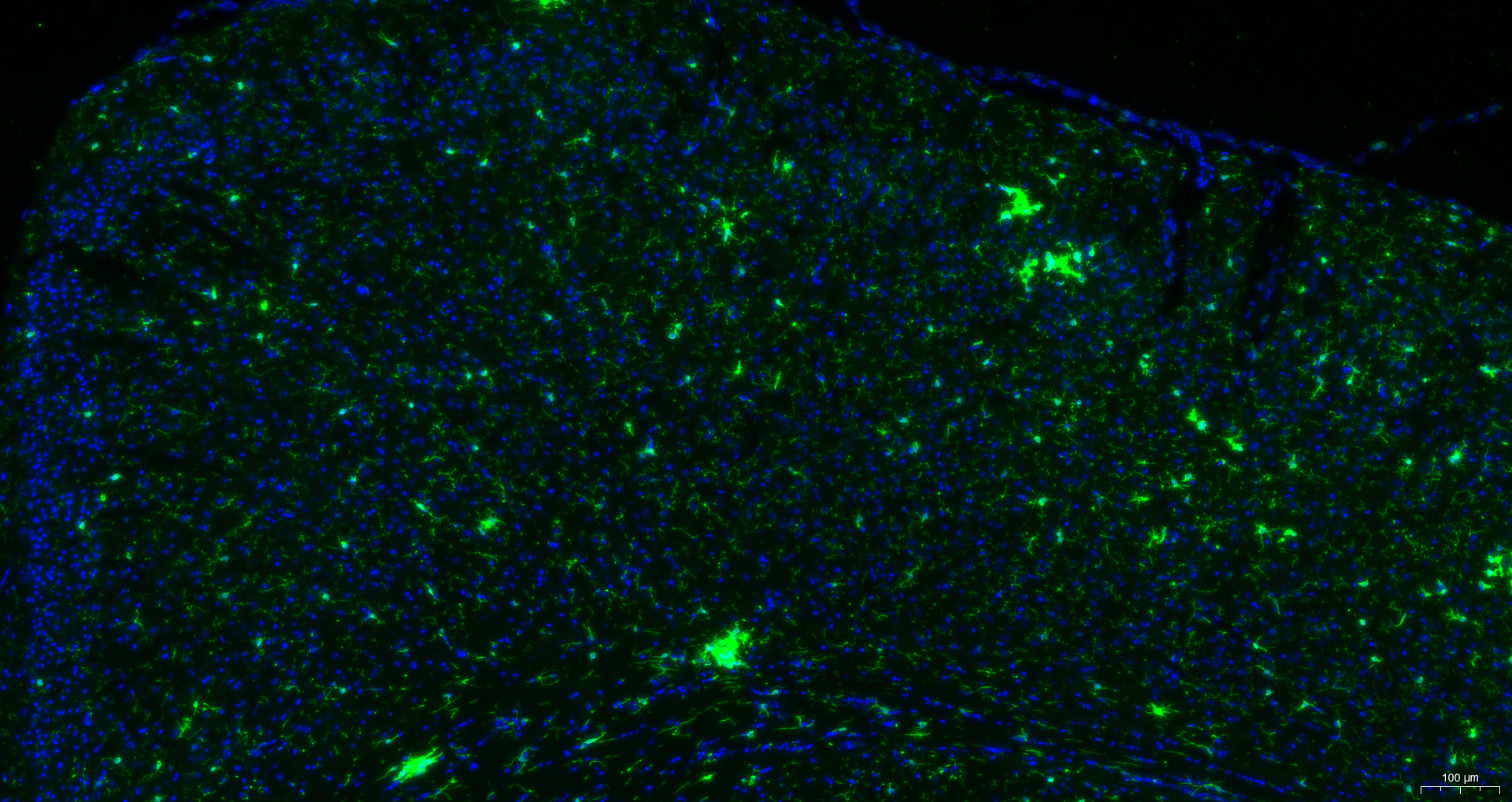

Supplement: Supplemental Information 3 [file peerj-08-10262-s003.zip › raw data2-2/AD+BI/4-M13_10.0x.png]

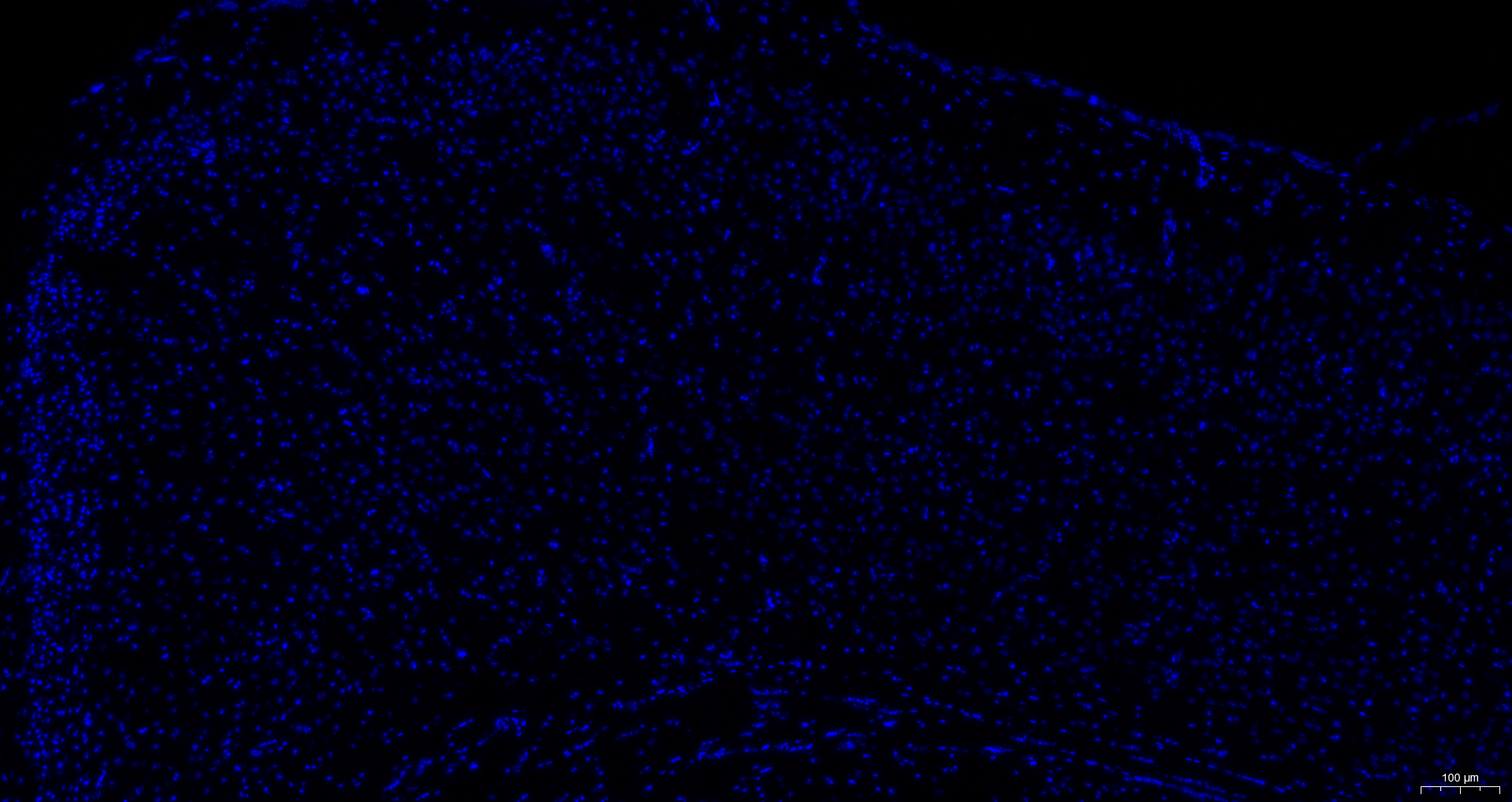

Supplement: Supplemental Information 3 [file peerj-08-10262-s003.zip › raw data2-2/AD+BI/4-M1_10.0x.png]

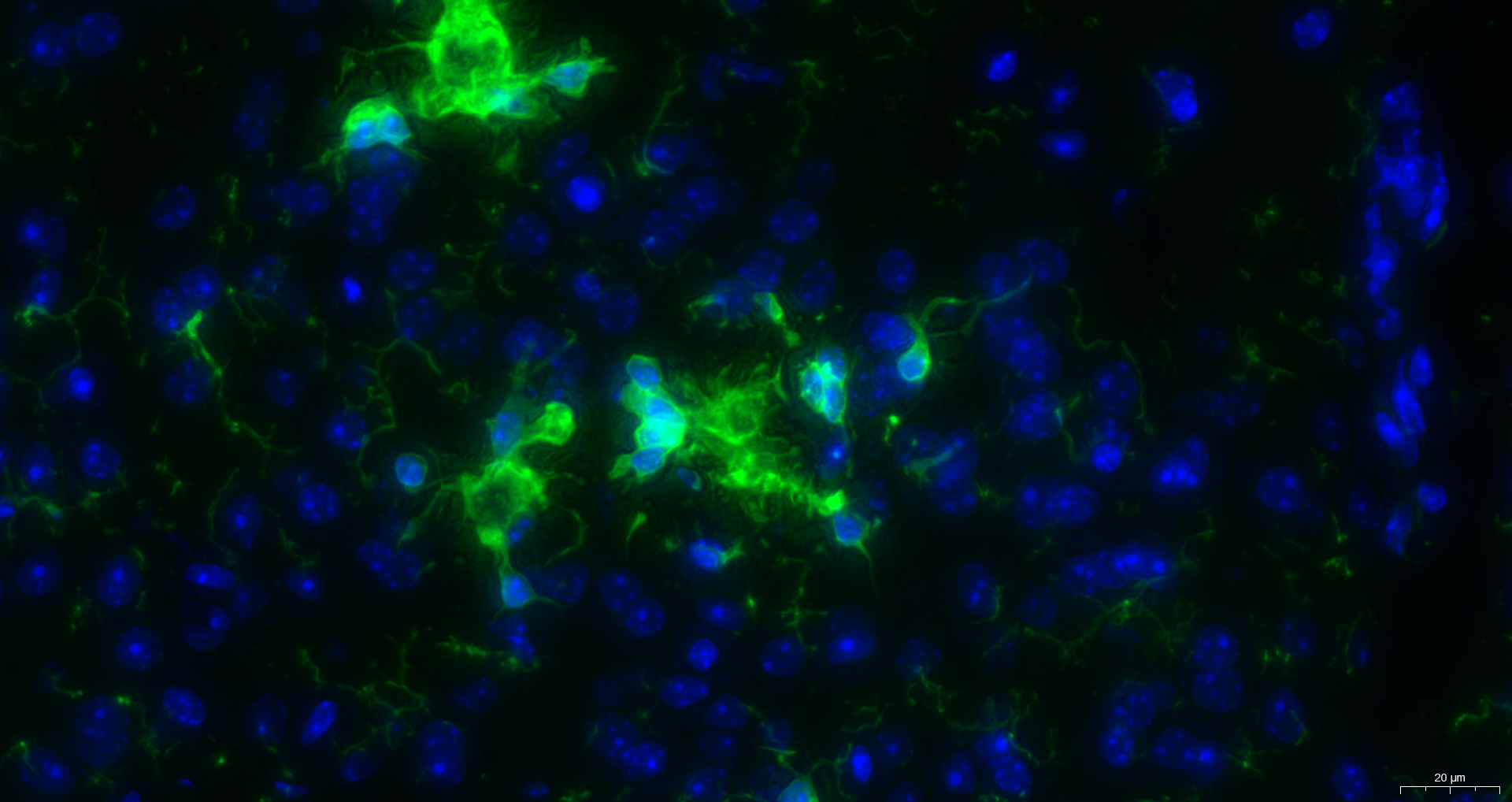

Supplement: Supplemental Information 3 [file peerj-08-10262-s003.zip › raw data2-2/AD+BI/4-M1_63.0x.png]

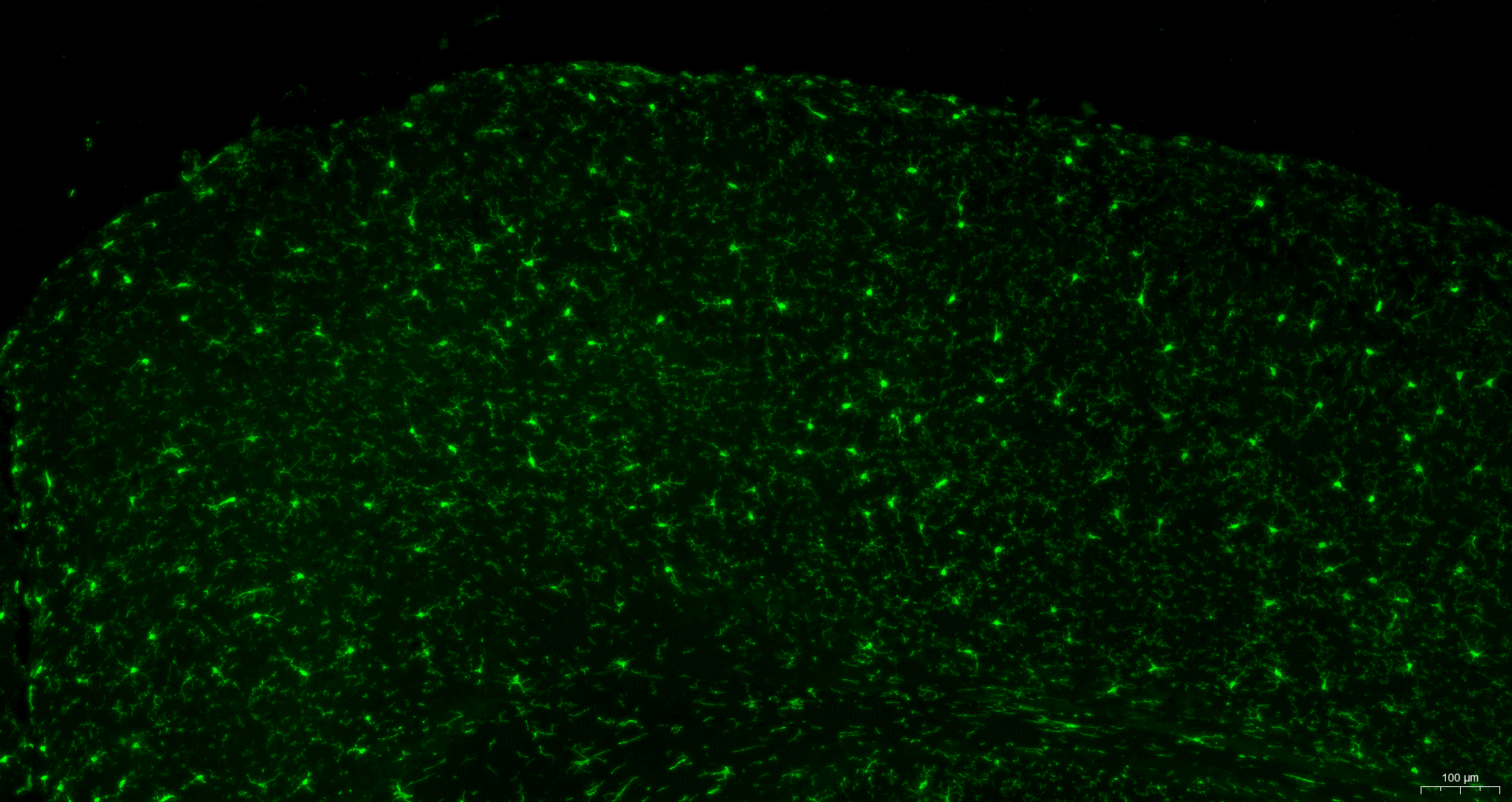

Supplement: Supplemental Information 3 [file peerj-08-10262-s003.zip › raw data2-2/WT/4-J12_10.0x.png]

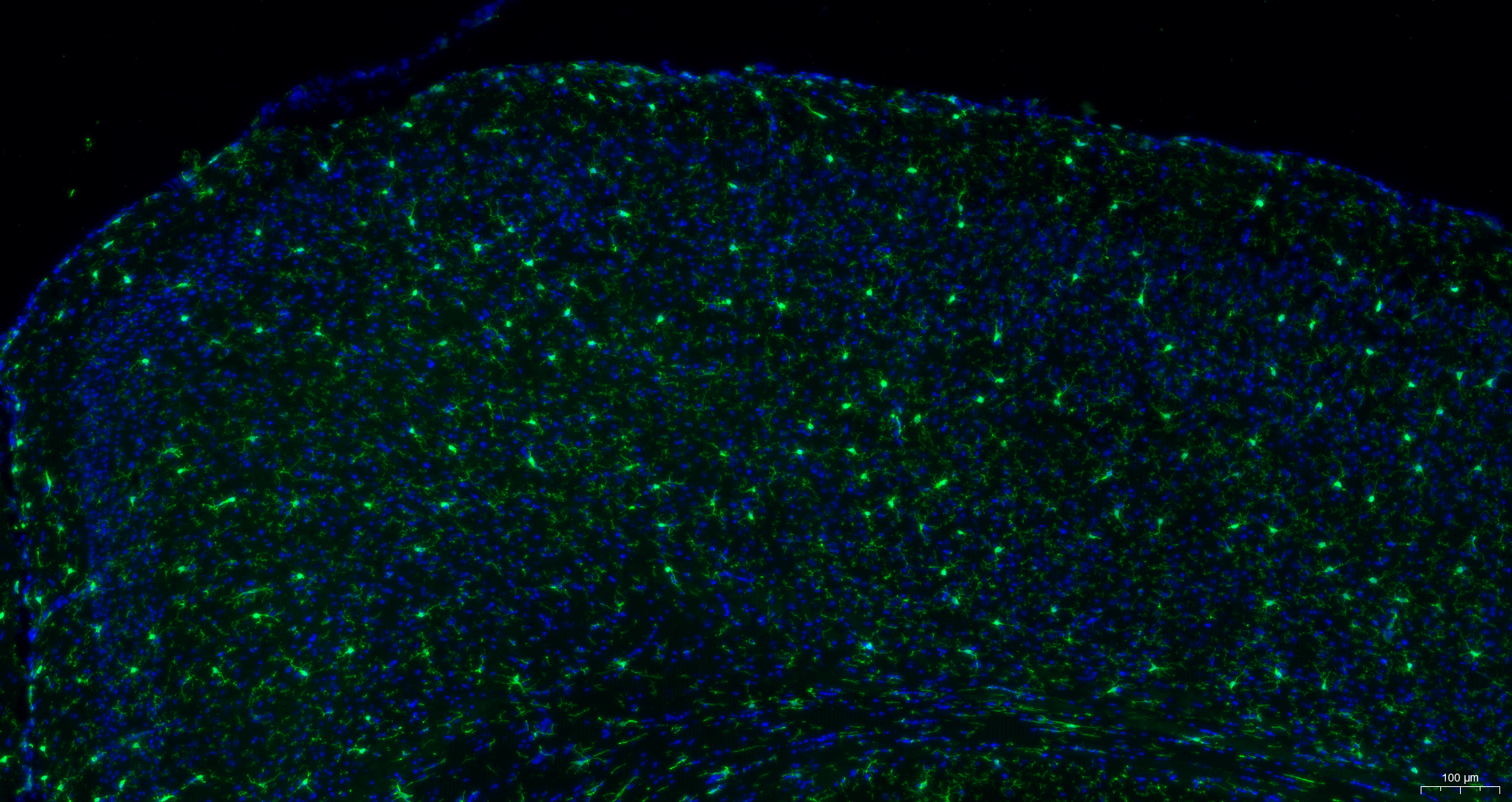

Supplement: Supplemental Information 3 [file peerj-08-10262-s003.zip › raw data2-2/WT/4-J13_10.0x.png]

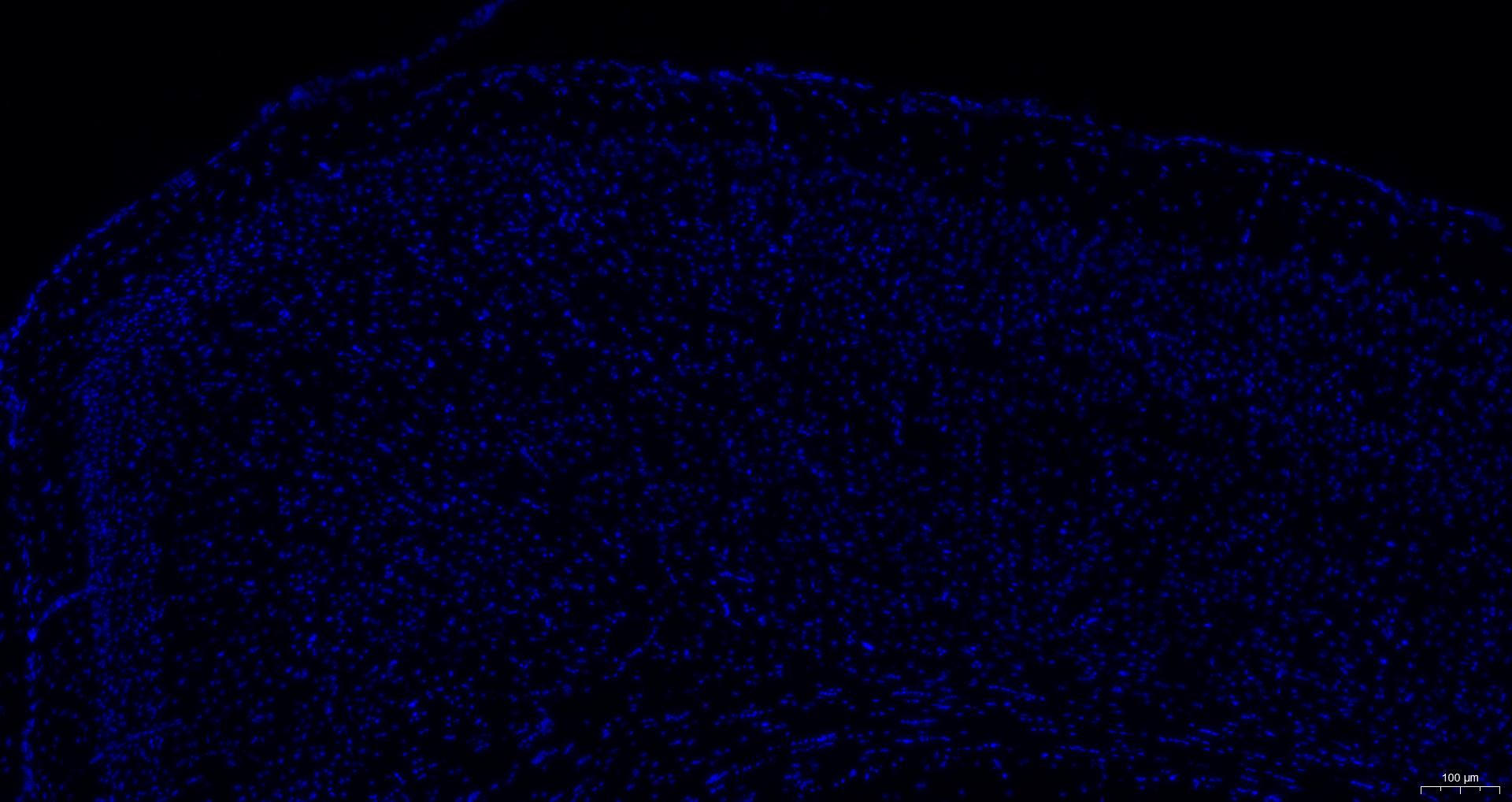

Supplement: Supplemental Information 3 [file peerj-08-10262-s003.zip › raw data2-2/WT/4-J1_10.0x.png]

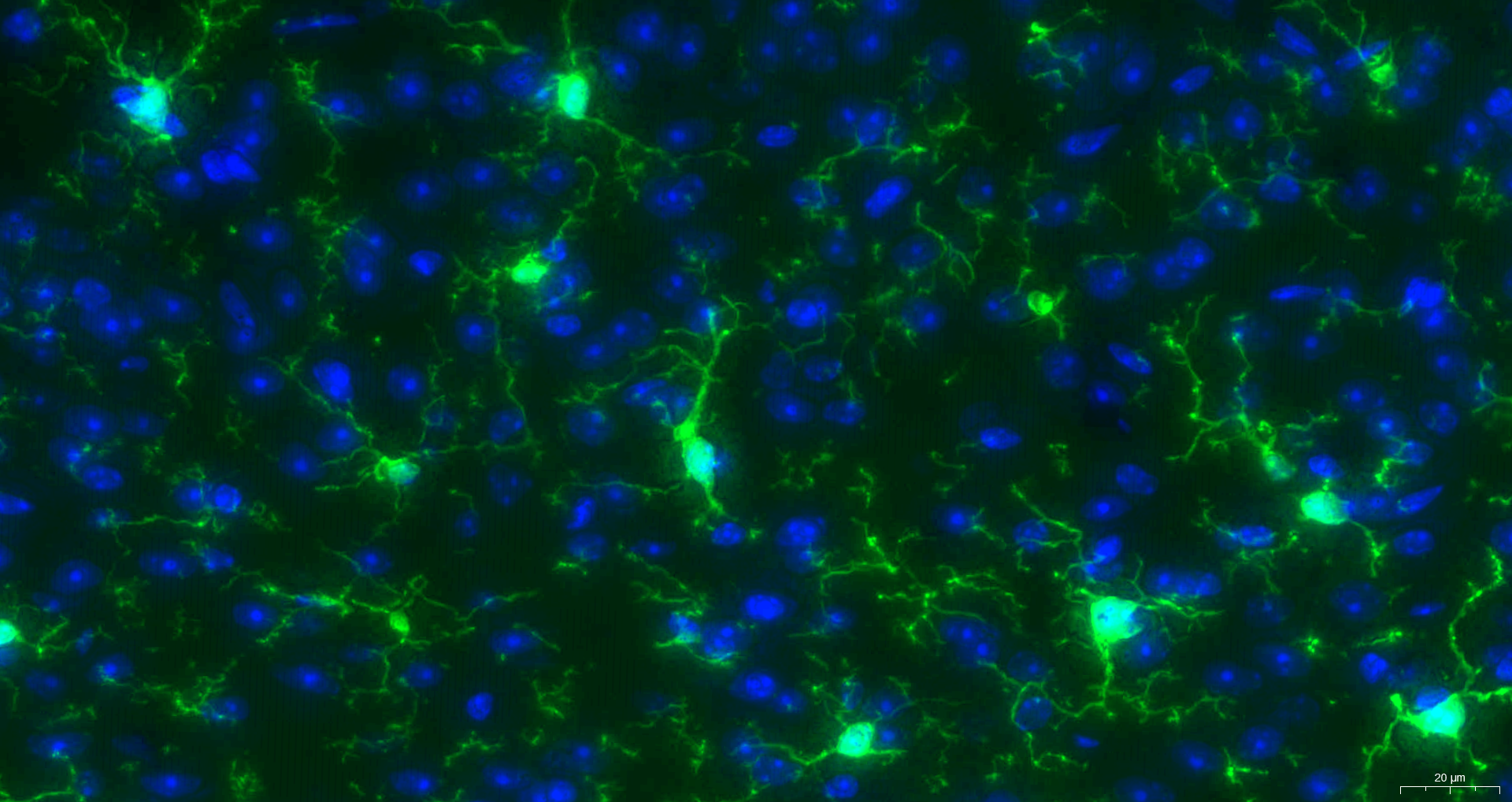

Supplement: Supplemental Information 3 [file peerj-08-10262-s003.zip › raw data2-2/WT/4-J1_63.0x.png]

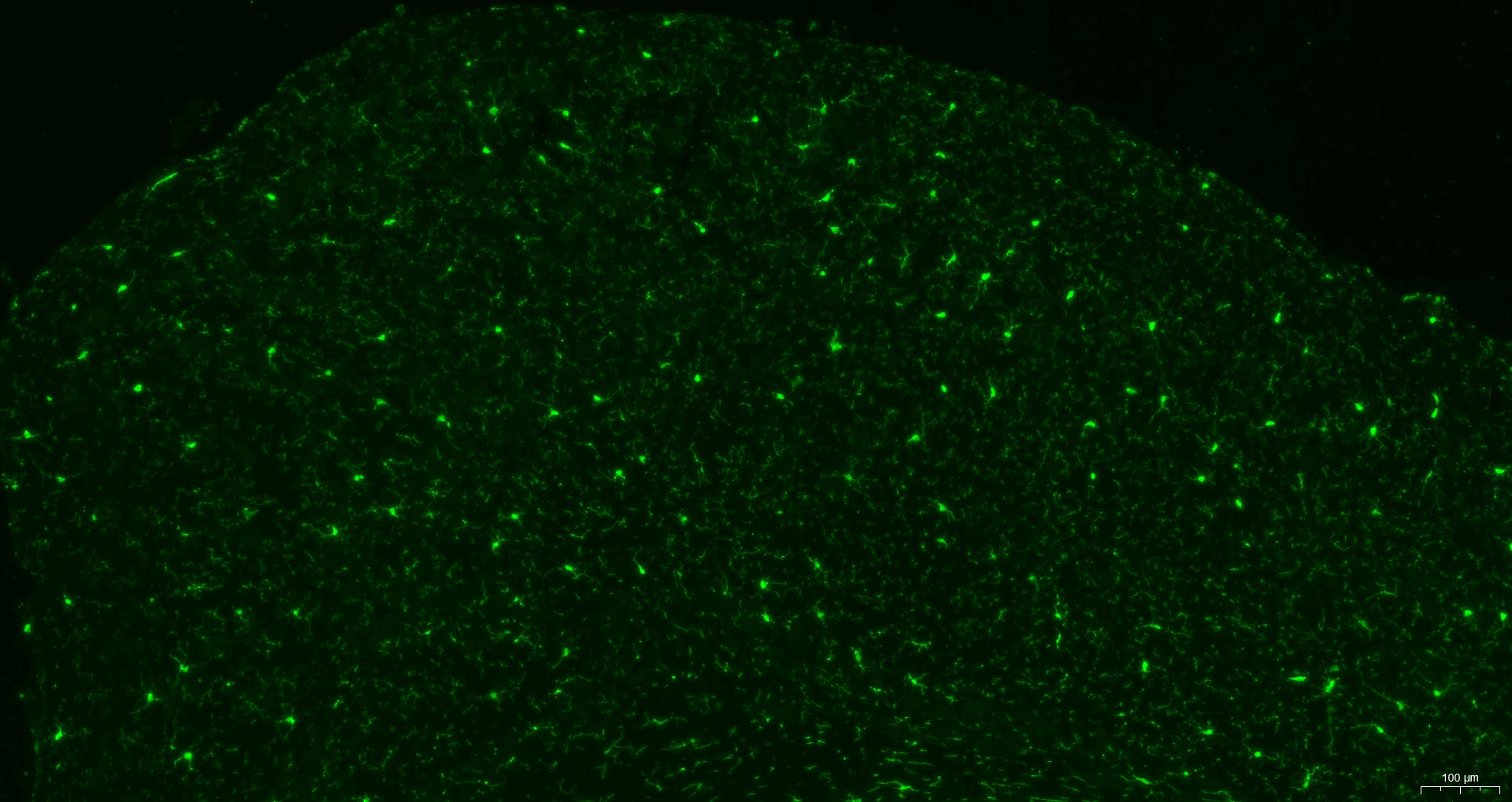

Supplement: Supplemental Information 3 [file peerj-08-10262-s003.zip › raw data2-2/WT +BI/4-K12_10.0x.png]

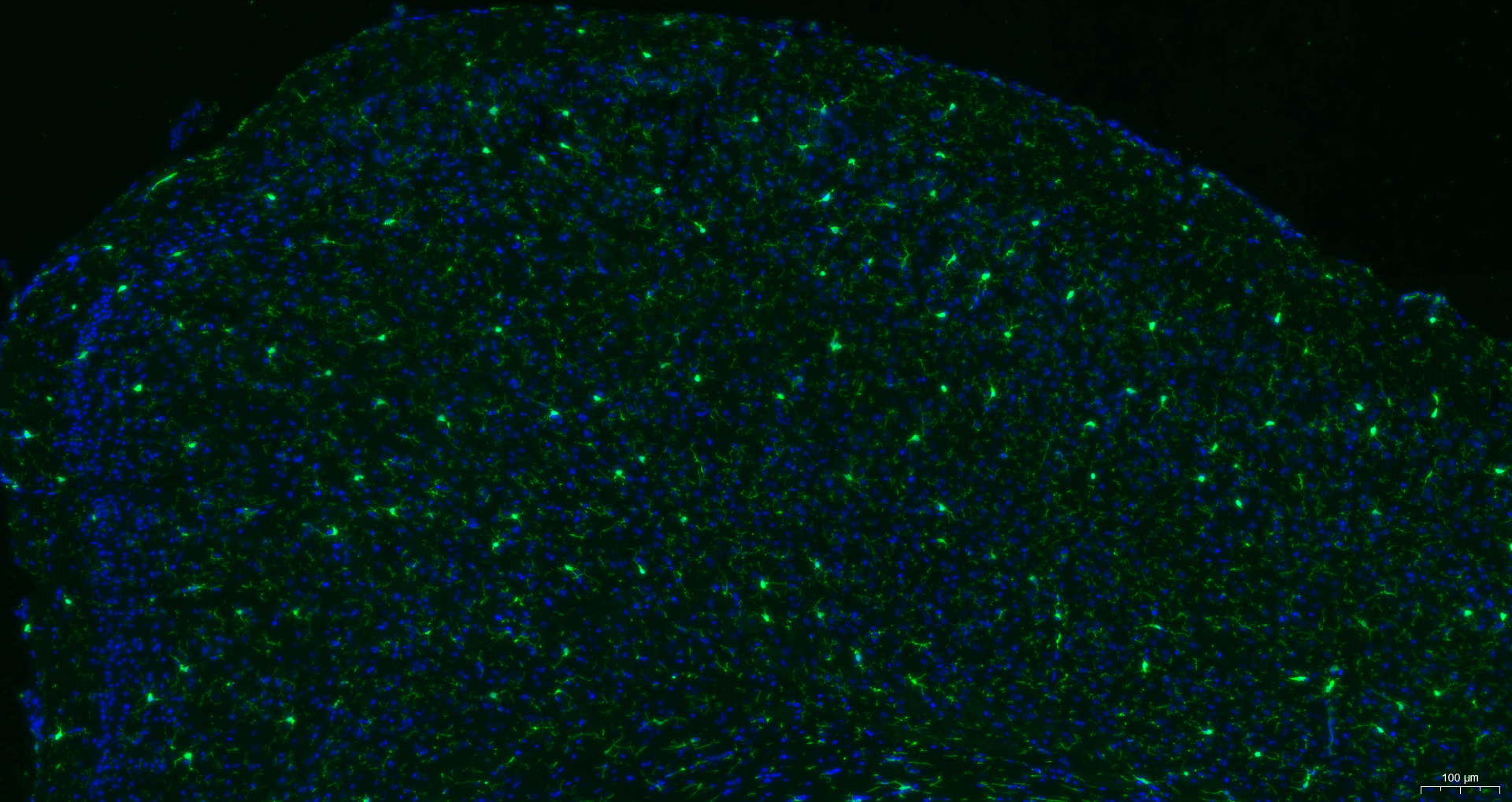

Supplement: Supplemental Information 3 [file peerj-08-10262-s003.zip › raw data2-2/WT +BI/4-K13_10.0x.png]

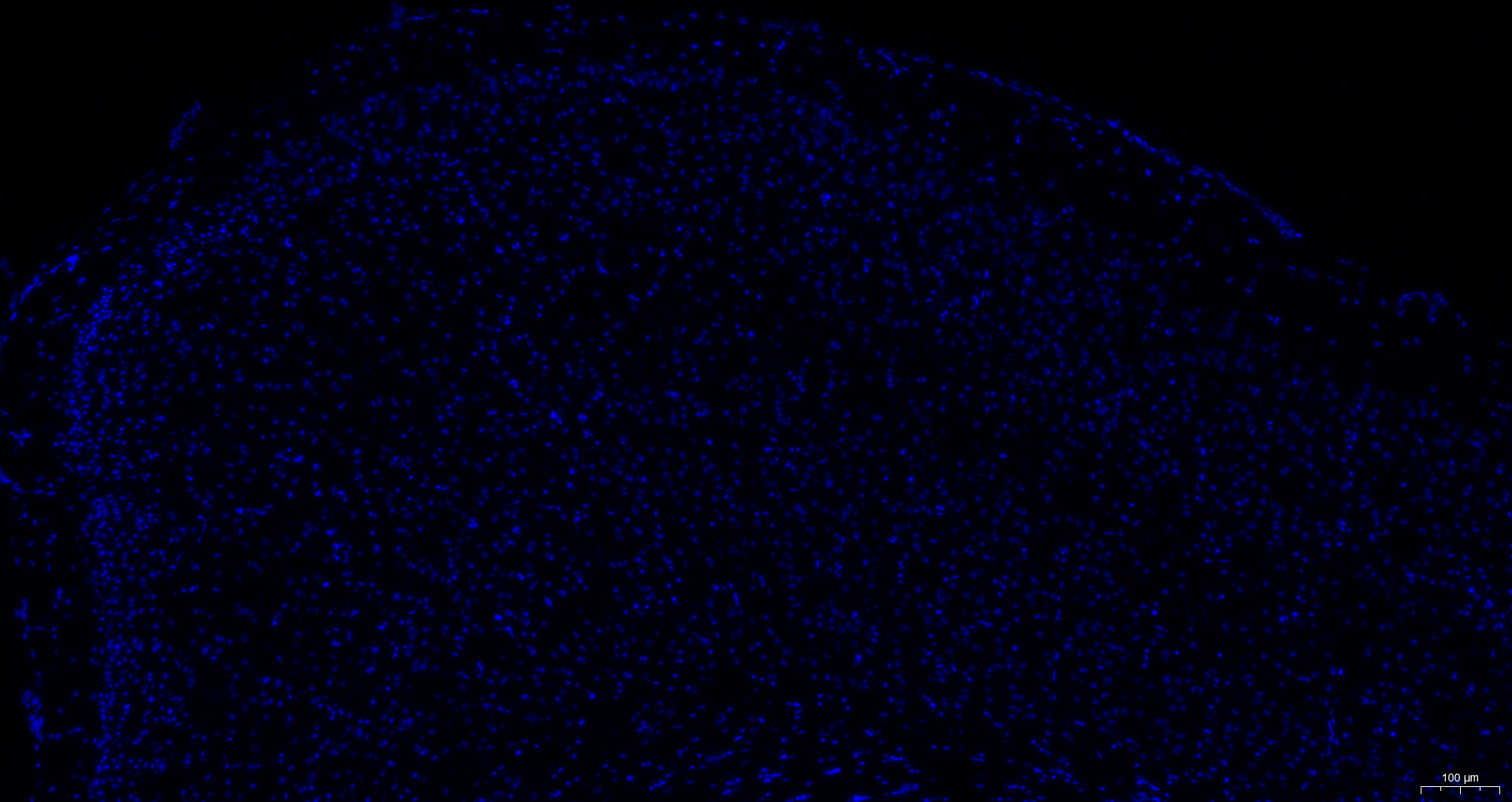

Supplement: Supplemental Information 3 [file peerj-08-10262-s003.zip › raw data2-2/WT +BI/4-K1_10.0x.png]

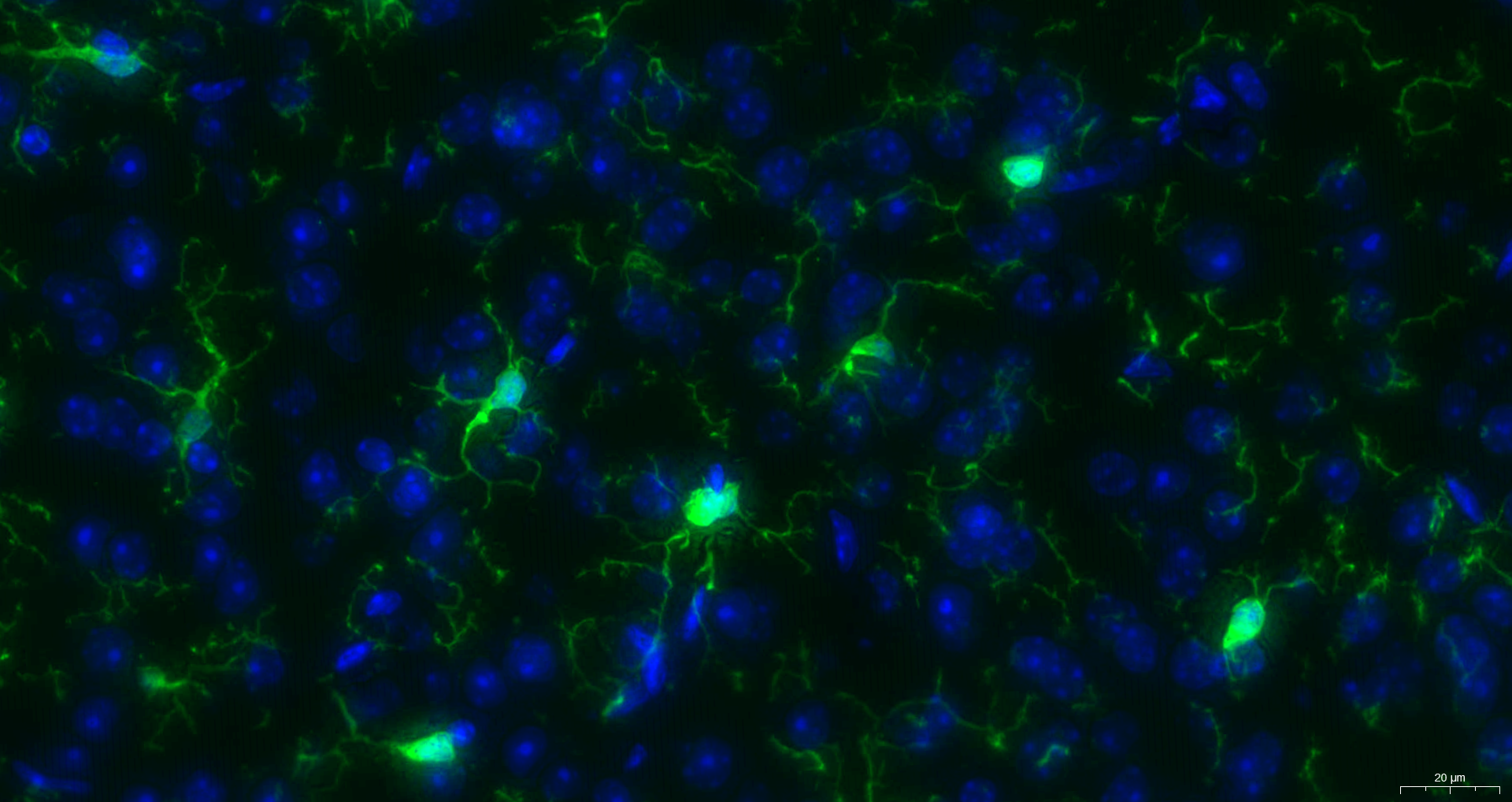

Supplement: Supplemental Information 3 [file peerj-08-10262-s003.zip › raw data2-2/WT +BI/4-K1_63.0x.png]
