# Supplementary material for: Treatment with Bifidobacteria can suppress Aβ accumulation and neuroinflammation in APP/PS1 mice
Source: PeerJ. 2020 Oct 28;8:e10262. doi: 10.7717/peerj.10262 (PMC7602682; doi:10.7717/peerj.10262)
Supplement: Supplemental Information 4 [file peerj-08-10262-s004.doc]

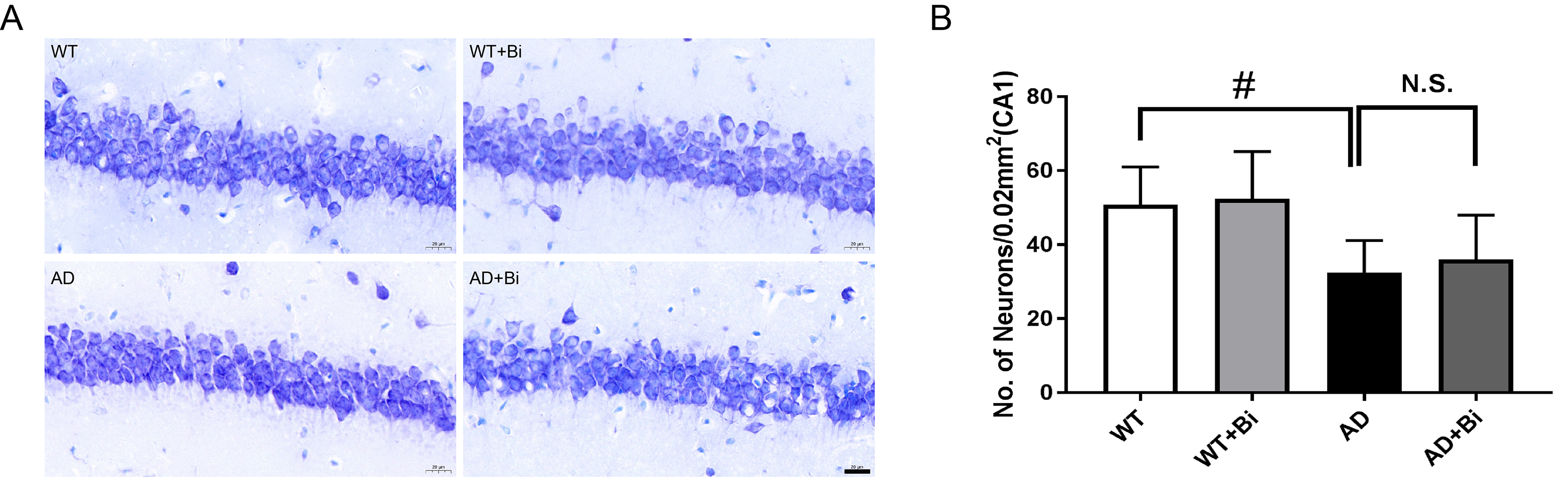


Fig S1 (A).Nissl staining in WT, WT+Bi, AD and AD+Bi group. Scale bar =20 μm. (B). Bar graph representation of estimated neuron numbers in CA1. # compared with WT group, #P < 0.05; n=6.


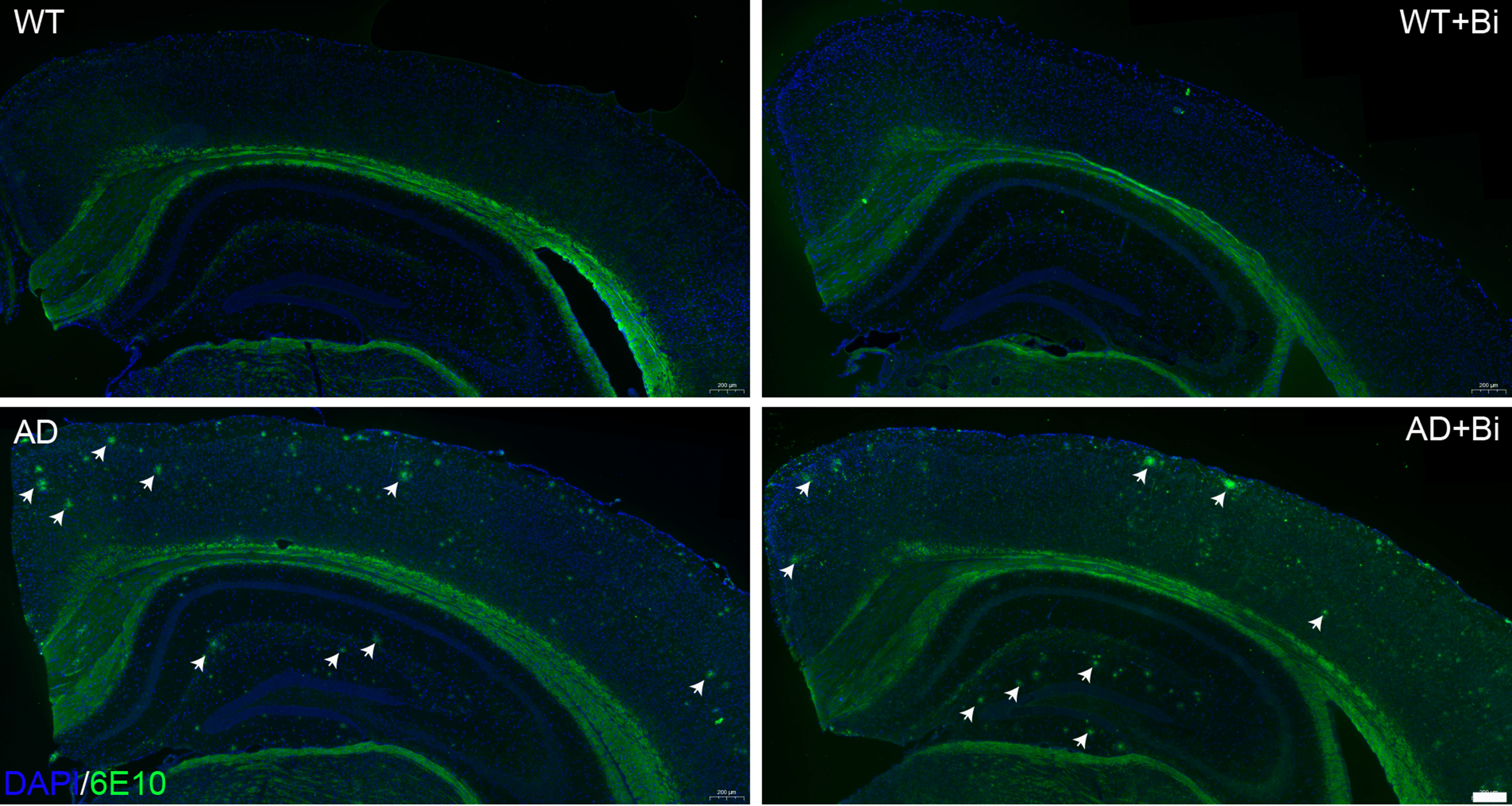


Fig. S2 Aβ plaques staining in WT, WT+Bi, AD and AD+Bi group. Arrows point to Aβ plaques. Scale bar =200 μm.
